# Helping patients help themselves: A systematic review of self-management support strategies in primary health care practice

**DOI:** 10.1371/journal.pone.0220116

**Published:** 2019-08-01

**Authors:** Sarah Dineen-Griffin, Victoria Garcia-Cardenas, Kylie Williams, Shalom I. Benrimoj

**Affiliations:** 1 Graduate School of Health, University of Technology Sydney, Sydney, Australia; 2 Emeritus Professor, University of Sydney, Sydney, Australia; Universite de Bretagne Occidentale, FRANCE

## Abstract

**Background:**

Primary health professionals are well positioned to support the delivery of patient self-management in an evidence-based, structured capacity. A need exists to better understand the active components required for effective self-management support, how these might be delivered within primary care, and the training and system changes that would subsequently be needed.

**Objectives:**

(1) To examine self-management support interventions in primary care on health outcomes for a wide range of diseases compared to usual standard of care; and (2) To identify the effective strategies that facilitate positive clinical and humanistic outcomes in this setting.

**Method:**

A systematic review of randomized controlled trials evaluating self-management support interventions was conducted following the Cochrane handbook & PRISMA guidelines. Published literature was systematically searched from inception to June 2019 in PubMed, Scopus and Web of Science. Eligible studies assessed the effectiveness of individualized interventions with follow-up, delivered face-to-face to adult patients with any condition in primary care, compared with usual standard of care. Matrices were developed that mapped the evidence and components for each intervention. The methodological quality of included studies were appraised.

**Results:**

6,510 records were retrieved. 58 studies were included in the final qualitative synthesis. Findings reveal a structured patient-provider exchange is required in primary care (including a one-on-one patient-provider consultation, ongoing follow up and provision of self-help materials). Interventions should be tailored to patient needs and may include combinations of strategies to improve a patient’s disease or treatment knowledge; independent monitoring of symptoms, encouraging self-treatment through a personalized action plan in response worsening symptoms or exacerbations, psychological coping and stress management strategies, and enhancing responsibility in medication adherence and lifestyle choices. Follow-up may include tailored feedback, monitoring of progress with respect to patient set healthcare goals, or honing problem-solving and decision-making skills. Theoretical models provided a strong base for effective SMS interventions. Positive outcomes for effective SMS included improvements in clinical indicators, health-related quality of life, self-efficacy (confidence to self-manage), disease knowledge or control. An SMS model has been developed which sets the foundation for the design and evaluation of practical strategies for the construct of self-management support interventions in primary healthcare practice.

**Conclusions:**

These findings provide primary care professionals with evidence-based strategies and structure to deliver SMS in practice. For this collaborative partnership approach to be more widely applied, future research should build on these findings for optimal SMS service design and upskilling healthcare providers to effectively support patients in this collaborative process.

## Introduction

Internationally, healthcare systems are challenged with the rising rates of chronic and complex illness and the clinical and economic burden associated represents a major challenge to the optimal provision of healthcare [1]. Health systems need to accommodate changes to meet the increasing need for health services. Evidence suggests that leveraging the potential of people to care for themselves and involving patients in decisions affecting their health is beneficial, particularly on the increasing rates of primary care consultations and health system pressures [2]. A key issue that needs to be addressed is how primary health care professionals (HCPs) can support self-management in an evidence-based, structured way and how self-management processes can be integrated into clinical practice, as models of care evolve to deliver a person-centred approach. Patient participation is suggested to narrow the gap between the dichotomous roles of patient and HCP [3]. Patient participation involves being engaged in the planning of care and exchanging knowledge, setting own goals and carrying out self-management activities [3]. This partnership has been suggested as valuable in the support of the management and control of symptoms, particularly for patients with chronic health conditions [4]. Self-management strategies are increasingly recognized as an essential component of chronic disease management and secondary prevention [5], individually tailored to patient preferences, prior knowledge and circumstances, supporting patient participation in their care [6].

Self-management support (SMS) is viewed in two ways: (1) as a portfolio of techniques and tools that help patients choose healthy behaviours, and (2) as a fundamental transformation of the patient-professional relationship into a collaborative partnership [7]. SMS encompasses more than a didactic, instructional program and goes beyond simple dissemination of information or disease state management. The pivotal objective of SMS is to change behaviour within a collaborative arrangement to produce sustainable effects. This can be achieved by increasing patients’ skills and confidence in managing their disease state through regular assessment of progress and problems, goal setting, and problem-solving support [8]. Simply put, patients and HCPs work to develop tangible and realistic healthcare goals, while HCPs can assist with the development of the skill set necessary to achieve these goals and monitor for improvements in patient health [9]. Lorig and Holman [10] identify a generic set of skills proven successful for effective self-management, including (1) problem-solving; (2) decision-making; (3) resource utilization; (4) forming a patient-health care provider partnership; and (5) taking action. Acquisition of these skills leads to increased self-efficacy. Self-efficacy refers to beliefs in one’s capabilities to execute a behaviour or course of action necessary to reach a desired goal [10, 11].

There is a growing body of evidence that shows supporting people to self-manage their health and care can lead to improvements in clinical and humanistic outcomes [12–18], reducing the economic impact of chronic disease and a means of contributing to the sustainability of the global healthcare system. Supporting people to self-manage has resulted in reduced use of general practitioners, reduced admissions to hospital, significant gains in health status and increased symptom control [19, 20]. Interventions have targeted patients with arthritis [21], asthma [22], chronic heart failure (CHF) [23], chronic obstructive pulmonary disease (COPD) [24], type 2 diabetes mellitus (T2DM) [25, 26], hypertension (HT) [27] and patients on oral anticoagulation [28]. Self-management support interventions vary in the literature with increasing evaluations of peer-led, lay-led, or non-health professional-led, web-based and group-based interventions. For example, the generic Chronic Disease Self-Management Program, a non-health professional group-delivered intervention remains the most widely adopted self-management support program internationally [29].

Primary HCPs are typically an individuals’ first point of contact with the health system [30], and are continuing contacts for people with chronic disease. This opens up substantial opportunities to effect sustainable changes through supporting self-management and delivery of more personalized healthcare services. There is an increasing number and uptake of primary care services which require HCPs to be patient-oriented however none of the education provided appears to include any theoretical framework or evidence-based structure for providers to effectively support self-management and facilitate patient behaviour change. Importantly, HCPs need to acquire the competencies not only to identify the techniques and tools for specific patients but to ensure that patients acquire the skills to self-manage. Kennedy et al. recommends a whole systems approach, which integrates SMS at the level of the patient, HCP, and service organizations, which has proven effective in improving outcomes for patients [31]. Effective implementation is profoundly important to ensure viability and sustainability, and potential scale-up. In some countries, governments have developed health policy and funding alignment for self-management support with the aim of improving health outcomes and alleviating pressures on the wider health system [32].

While the role of primary HCPs in delivering SMS is highlighted in the literature, there remains a gap in research regarding the specific strategies and active components of interventions used by providers resulting in better health outcomes for patients. A need exists to better understand how these might be delivered within primary care, what outcomes can be achieved, and the training and system changes needed as a result. This gap increases the challenge of providing consistent SMS in primary care, and enabling the appropriate evaluation of SMS trials. Therefore, the objective of this systematic review is to summarize the evidence of effectiveness for SMS interventions delivered face-to-face in primary care practice, and identify evidence-based strategies with active components facilitating positive clinical and humanistic patient outcomes.

## Methods

A systematic review of randomized controlled trials evaluating SMS interventions was conducted following the Cochrane Handbook for Systematic Reviews of Interventions. We have reported the review according to PRISMA (Preferred Reporting Items for Systematic reviews and Meta-Analyses) guidelines [33, 34]. Details of the protocol for this systematic review can be found in the PROSPERO international prospective register of systematic reviews database (registration CRD42017062639).

### Search strategy

The research question (using PICO) and search strategy were developed and reviewed by three authors (SDG, VGC, SB) to identify studies for this review. In a preliminary scoping search of databases, we as a group of authors identified ten key papers which were suitable to be included in the review. In the multiple search strategies all authors were involved. We tested and refined our strategies as a group, which ensured reproducibility of key papers within search results and a robust search strategy. The detailed search strategy for different electronic databases can be found in S1 Table. A comprehensive search was undertaken in three databases using PubMed, Scopus and Web of Science and search strategies were refined for each individual database. Multiple databases were searched to adequately identify all literature relevant to the research question. Published literature was systematically searched from inception to June 2019. Neither publication date nor publication type filters were used. Citation searching was also conducted to find articles cited by other publications. Searches of grey literature and reference lists of previous systematic reviews complemented our literature search to ensure all relevant studies were captured. The complete results from all databases were imported and managed in a unique EndNote X9 library upon search completion and saved without duplication.

### Data extraction, management and synthesis

The review team were responsible for assessing the trials’ eligibility using the methods outlined. The lead reviewer (SDG) screened by title and abstract to select relevant publications. A second and third reviewer (VGC, SB) were consulted throughout this process if an article could not be rejected with certainty. Any disagreement among the reviewers throughout this process were resolved by discussion and consensus. All authors (SDG, VGC, KW, SB) agreed on the final texts for inclusion. Full texts were assessed for eligibility according to inclusion and exclusion criteria. Eligible studies were randomized controlled trials (RCTs) and cluster-randomized controlled trials (c-RCTs) assessing SMS interventions with follow-up, delivered by primary HCPs, face-to-face to adult patients with any condition, compared to usual standard of care. The types of interventions included in the review were multicomponent interventions aimed at supporting patient self-management. Jonkman et al’s definition of SMS interventions was applied for the purposes of selection of interventions for inclusion in this review [35]. This definition includes the wide range of components considered for ‘self-management interventions’. Self-management interventions are defined as [35]:

“*Interventions that aim to equip patients with skills to actively participate and take responsibility in the management of their chronic condition*. *This includes knowledge acquisition*, *and a combination of at least two of the following*: *(1) stimulation of independent sign and/or symptom monitoring; (2) medication management; (3) enhancing problem-solving and decision-making skills for treatment or disease management; (4) or changing physical activity*, *dietary and/or smoking behaviour*”.

Excluded studies were: (1) non-randomized controlled study designs; (2) interventions not meeting Jonkman’s definition of self-management support; (3) interventions not delivered face-to-face (i.e. web-based interventions); (4) group-delivered interventions; (5) study populations under 18 years of age; (6) interventions delivered in settings other than primary care; (7) interventions delivered by non-HCPs (i.e. lay, peer-led); (8) studies without usual standard of care as comparator; (9) studies written in a language other than English or Spanish; or (10) non-primary research articles (i.e. literature reviews, study protocols).

Authors kept a record of the number of trials included or excluded from the review at each stage of the assessment process. Multiple papers of the same study were linked together. Study design, setting, methods, participant characteristics, type of intervention, content, duration and intensity of components, follow up, and study findings were extracted using a tailored data extraction form developed for data retrieval using the Cochrane Handbook for Systematic Reviews of Interventions [36] and the Cochrane Effective Practice and Organisation of Care Group (EPOC) data collection form [37] and checklist [38].

Matrices were developed mapping both evidence and active components for each self-management intervention. Outcome indicators were independently extracted, tabulated and grouped using the following categories of outcome measures, including (1) disease specific indicators; (2) self-efficacy; (3) health-related quality of life; (4) functional status and disability; (5) psychological functioning; (6) disease knowledge; (7) behaviours and self-management activities. Components were categorized according to Jonkman’s definition of SMS interventions [35], including strategies for: (1) condition or treatment knowledge acquisition; (2) active stimulation of symptom monitoring; (3) self-treatment through the use of an action plan; (4) enhancing resource utilization; (5) enhancing problem-solving and/ or decision-making skills; (6) enhancing stress management or emotional coping with condition; (7) enhancing physical activity; (8) enhancing dietary intake; (9) enhancing smoking cessation; and (10) medication management or adherence. Given the heterogeneity of the studies regarding participants, varying healthcare setting, strategies and outcome measures, no formal quantitative synthesis or meta-analysis could be conducted.

### Assessment of risk of bias

The methodological quality of studies were appraised using the ‘Suggested risk of bias criteria for EPOC reviews’ tool in accordance with the Cochrane Handbook [39]. Domains of bias included in the final assessment, were: (1) random sequence generation; (2) allocation concealment; (3) similarities on baseline outcome measurements; (4) similarities on baseline characteristics; (5) completeness of outcome data; (6) blinding (participants, personnel); (7) protection against contamination; (8) selective outcome reporting; and (9) other risks of bias. Studies were assessed by domain as 'low risk' or 'high risk' of bias. Domains were ‘unclear risk’ if too few details were available to make an acceptable judgement of ‘high’ or ‘low’ risk. A second and third reviewer (VGC, SB) were consulted throughout this process if decisions could not be made with certainty. Any disagreement among the reviewers throughout this process were resolved by discussion and consensus. Three categories of study quality were identified by study authors according to each study’s methodological characteristics. In high-quality studies, the majority of criteria were fulfilled and done well (low risk of bias in at least six criterion), while in low-quality studies, the majority of criteria were not done or done poorly (high risk of bias in at least five criterion); other situations were considered medium quality [40]. No papers were excluded as a result of quality assessment.

## Results

### Study selection

**Fig 1:**
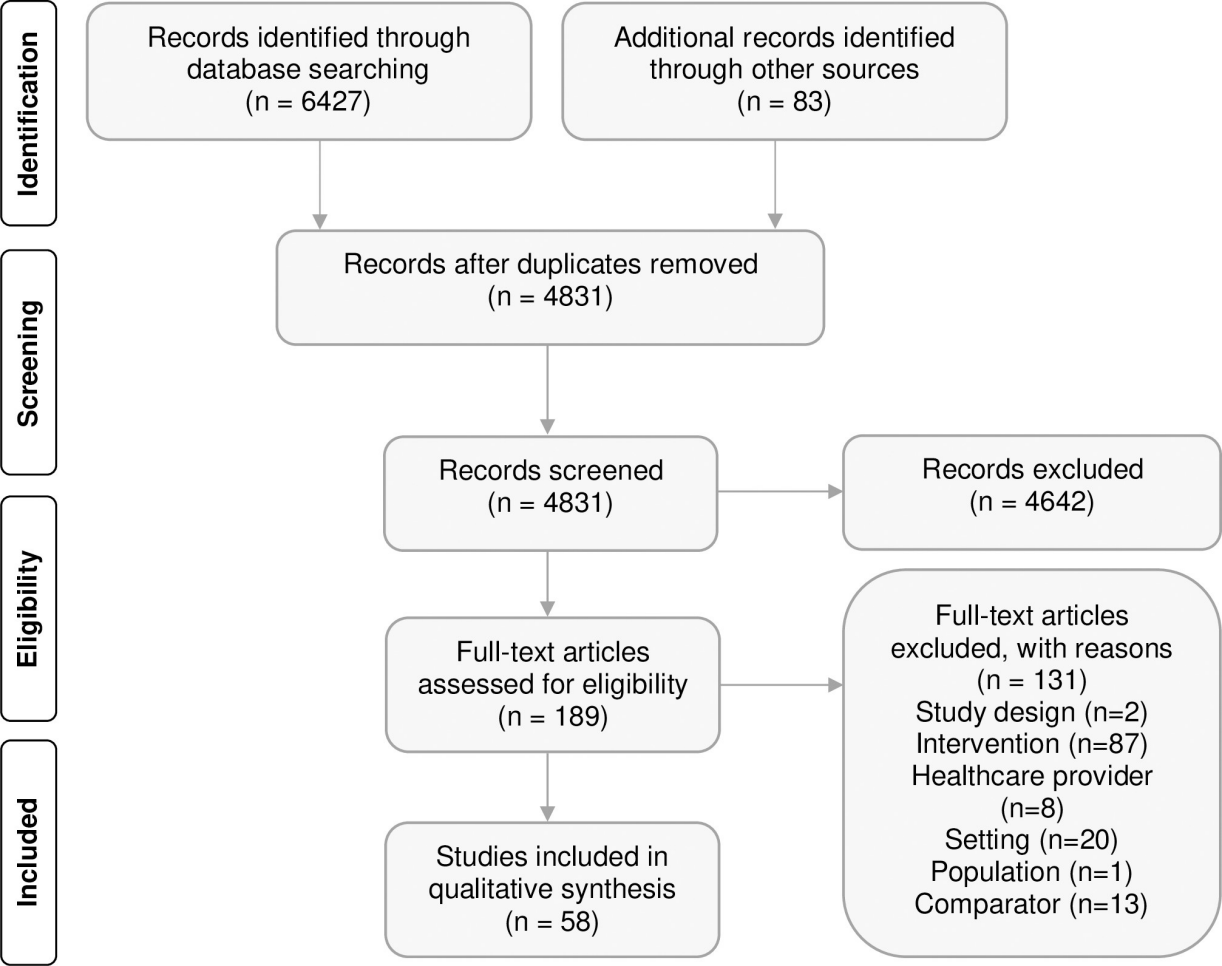
PRISMA diagram of search results and screening.

6,510 citations were retrieved. After the removal of duplicates, 4,831 records were screened by title and abstract. After review of full texts, fifty-eight RCTs/c-RCTs (reported in 80 citations) fulfilled the review criteria and were included in this systematic review (see flow diagram in Fig 1). A completed PRISMA checklist can be found in S2 Table. Descriptive characteristics of individual studies are provided in S3 Table.

### Description of studies

The included studies originated from 18 countries, predominantly the United Kingdom (UK) and the United States (US). The conditions most frequently targeted included T2DM (37.9%; n = 22), COPD (20.7%; n = 12) and depression (13.8%; n = 8) (Table 1). Settings primarily reported were general practice (48.3%; n = 28), primary care clinics (25.9%; n = 15) and community pharmacies (10.3%; n = 6). Interventions were delivered largely by general practitioners or nurses, commonly specialising in areas such as respiratory, diabetes and mental health. SMS interventions in fourteen studies were delivered in primary care teams involving more than one health care professional from different disciplines (24.1%; n = 14).

**Table 1:** Classification of self-management support studies by condition.

Condition	Frequency (N)	Associated references
Diabetes	22	[31, 41–63]
COPD	12	[31, 58, 59, 61–71]
Depression	8	[47, 58, 72–77]
Coronary heart disease	5	[47, 58, 76, 78, 79]
Asthma	5	[59, 62, 63, 80–82]
Osteoarthritis (OA)	4	[58, 83–87]
Low back pain	2	[88, 89]
Irritable bowel syndrome (IBS)	3	[31, 61, 90, 91]
Cardiovascular disease (CVD)	3	[55, 59, 62, 63]
Recurrent binge eating/ binge eating disorder	2	[92, 93]
Unexplained chronic fatigue & chronic fatigue syndrome	2	[94, 95]
Anxiety	2	[73, 74]
Chronic dizziness	1	[96]
Bulimia nervosa	1	[97]
Hypertension	1	[58]
Migraine/ headache	1	[98]
Oral hygiene	1	[99]
Low self-esteem	1	[100]
Psychosocial problems	1	[101]
Schizophrenia	1	[58]
Bipolar	1	[58]
Congestive heart failure	1	[58]
Hyperlipidaemia	1	[62, 63]
Prediabetes	1	[62, 63]

### Study outcomes

Ninety-three different outcome measures were adopted by studies. Clinical outcome measures associated with a particular condition were typically reported (e.g. clinical outcomes such as changes in blood pressure or HbA1c levels). Humanistic outcomes sought to measure physical, social and psychological functioning and changes in health-related quality of life (HRQOL). Others captured changes in self-efficacy. Results were classified by outcome and method of assessment (summarised in S4 Table relative to key findings).

### Impact of interventions on outcomes

The overall impact of interventions on clinical and humanistic outcomes are illustrated in Table 2.

**Table 2:** Evidence of SMS interventions on desired outcomes.

	Disease specific outcomes	Self-efficacy	Quality of life	Physical and social functioning	Psychological functioning	Disease knowledge	Health behaviours	Self-management activities
Adachi et al. [50]	±							
Banasiak et al. [97]	±			+	+			
Barbanel et al. [80]	+							
Barley et al. [76]	NEA	NEA	NEA	NEA	NEA			
Bartels et al. [58]				NS				±
Bischoff et al. [69]	NS	NS	NS					
Broderick et al. [84]	±	±	NS	±	+			
Browning et al. **[54, 102]**	±	NS	NS		+			±
Chalder et al. [95]	+			+				
Cherkin et al. [88]	NS			NS		+	+	
Clark et al. [41, 103]	NEA						NS	+
Clarkson et al. [99, 104]	±	+						
Doucette et al. [45]	NS							±
Dziedzic et al. [85–87]	NS	+		NS	NS			
Efraimsson et al. [66]			+			+	+	
Eikelenboom et al. [59, 105]		NS					NS	
Farmer et al. [48, 106, 107]	NS							
Ferrone et al. [71]	+		+			+		
Fortin et al. [62, 63]				+				+
Freund et al. [101]			±					
Friedberg et al. [94, 108]	+		NS		NS			
Gabbay et al. [43]	±				+			
Gabbay et al. [60]	±		NS		NS			NS
Goudswaard et al. [42]	+							
Grilo et al. [93]	NS				NS	NS		
Heitkemper et al. [91]			+		+			
Hill et al. [67]						+		
Hoffmann et al. [98]	NS	NS	±					
Huang et al. [46, 109]	±							
Ismail et al. [49]	NS							
Jaipakdee et al. [56]	±		+		NS			
Kennedy et al. [31, 61]		NS	NS	NS	NS			NS
McGeoch et al. [64]	NS		NS		NS			±
McLean et al. [82]	+		+			+		
Mehuys et al. [81]	±		NS			NS	±	
Mehuys et al. [52]	+					+		±
Meland et al. [78, 110]	NS	NS					NS	
Mitchell et al. [68, 111]	±	NS			±	+		
Morgan et al. [47, 112]	±				+			
Moss-Morris et al. [90, 113]	+		+		NS			
Murphy et al. [79, 114, 115]	NS			NS				
Olry de Labry Lima et al. [53]	±							
Partapsingh et al. [51]	NS							
Richards et al. [74]			NS		NS			
Rosemann et al. [83]			NS				NS	
Smit et al. [77, 116]		NS						
Striegel-Moore et al. [92, 117]	+		+		+			
Sturt et al. [44]	NS	+			+			
Tiessen et al. [55, 118]	NS						NS	
van Dijk-de Vries et al. [57]	NS	NS	NS	NS	NS	NS		
Von Korff et al. [89]	NS			NS	NS			
Waite et al. [100]					+			
Watkins et al. [72]	+				+			
Watson et al. [70]	NS							
Williams et al. [75]					+			
Wood-Baker et al. [65]	±		NS				NS	
Yardley et al. [96]	+		+	NS	NS			
Zimmermann et al. [73]	NS	+	NS		NS			

(+): positive findings; (±): mixed findings; (NS): non-significant findings; NEA: no evidence available

#### Disease specific outcomes

Forty four RCTs examined the impact of interventions on disease specific outcomes [42–57, 60, 70, 71, 76, 78, 79, 82, 85–87, 102, 106, 107, 109, 110, 112, 114, 115, 118]. Disease specific outcomes were most commonly reported in studies evaluating interventions targeting patients with T2DM (e.g. changes in HbA1c, weight, blood pressure and lipids), COPD (e.g. changes in Peak Expiratory Flow (PEF)), courses of antibiotics, oral corticosteroids and frequency of exacerbations), asthma (e.g. PEF, symptoms, inhalation technique, number of exacerbations and nocturnal awakenings), binge eating disorders (e.g. frequency of episodes and purging) and osteoarthritis (OA) (e.g. pain intensity, level of fatigue and use of pain medication). Seventeen studies targeting diabetes reported mean changes in HbA1c, with seven reporting significant improvements in the intervention compared to usual care [42, 46, 47, 50, 52, 53, 56, 109, 112]. Goudswaard et al. [42] reported a decrease in HbA1c at six weeks by 0.7% more (95% CI 0.1, 1.4) in those receiving the intervention when compared with control. The intervention evaluated by Adachi et al. [50] for patients with T2DM resulted in a 0.7% decrease in HbA1c at six months in the intervention group (n = 100) compared with a 0.2% decrease in the control group (n = 93) (difference −0.5%, 95% CI: -0.2%, −0.8%; p = 0.004).

Three RCTs reported on the level of asthma control and symptoms [80–82]. Mehuys et al. measured the level of asthma control using the Asthma Control Test (ACT), a clinically validated measure [81]. While mean ACT scores did not change from baseline for both study groups, a subgroup analysis of patients having insufficiently controlled asthma at baseline showed the intervention group had significantly increased ACT scores after six months (mean ACT change from baseline in the intervention group was +2.3 and +0.3 in the control group (mean difference 2.0, 95% CI: 0.1, 3.9; p = 0.038). The need for rescue medication was reduced in both groups from baseline, however a significantly higher reduction in the intervention group (-0.56 and -0.57 inhalations per day at three and six-month follow-up, respectively) was reported against control (-0.03 and -0.43 inhalations per day at three and six-month follow-up, respectively; p = 0.012) [81]. Six studies reported on COPD-specific outcomes [64, 65, 68–71, 111]. McGeoch et al. [64] reported no significant change in St. George’s Respiratory Questionnaire (SGRQ) as the primary outcome measure. The intervention also showed no effect on self-reported outcomes including the frequency of use of antibiotic courses and oral corticosteroids over 12 months [64].

Interventions targeting eating disorders were evaluated in four RCTs [41, 92, 93, 97, 103, 117]. Banasiak et al. [97] explored primary outcome measures of eating pathology derived from the Eating Disorder Examination Questionnaire (EDE-Q). Intention-to-treat (ITT) analyses revealed significant improvements in psychological symptoms at the end of the intervention compared with control, reduction in mean frequency of binge-eating episodes by 60% in intervention and 6% in control, and remission from all binge-eating and compensatory behaviours in 28% of the intervention and 11% of control. Treatment gains were maintained at three and six-month follow-up [97].

An intervention targeting patients with OA measured primary outcomes of pain intensity, physical functioning, self-efficacy, psychological distress, use of pain coping strategies, catastrophizing and HRQOL [84]. ITT analyses were performed on primary outcomes at baseline, post-treatment, 6 and 12 month follow-up which yielded significant group differences, indicating improvement in pain intensity (F(3,233) = 2.75, p = 0.044), physical functioning (F(3,233) = 3.11, p = 0.027), psychological distress (F(3,233) = 2.83, p = 0.039), use of pain coping strategies (F(3,233) = 4.97, p = 0.002), and self-efficacy (F(3,232) = 10.59, p< 0.001) in intervention, compared with control. All outcomes, except for self-efficacy, were maintained at 12-month follow-up while effects on self-efficacy degraded over time [84].

#### Health-related quality of life

Twenty-four RCTs examined the impact of interventions on HRQOL [31, 54, 56, 57, 60, 61, 64–66, 69, 71, 73, 74, 76, 81–84, 90–92, 94, 96, 98, 101, 102, 108, 113, 117]. The method of assessment varied and included general HRQOL questionnaires such as the SF-12 survey questionnaire and EuroQoL EQ-5D questionnaire. Disease specific QOL measures were also identified including the Arthritis Impact Measurement Scales Short Form questionnaire (AIMS2-SF) [119], Irritable Bowel Syndrome Quality of Life Questionnaire (IBSQOL) [120], Audit of Diabetes Dependent Quality of Life (ADDQOL) [121] and the standardised Asthma Quality of Life Questionnaire (AQLQ) [122]. Eight studies reported significant improvements in HRQOL [56, 66, 71, 82, 90–92, 96, 113, 117]. Efraimsson et al. [66] evaluated the effects of COPD self-management delivered at a nurse-led primary health care clinic. HRQOL, measured using the SGRQ, was improved by an average value of 8.2 units (from 30.6) in the intervention group, whereas no change was noted in control. Differences between groups were clinically relevant and statistically significant (p = 0.00030) [66]. Heitkemper et al. [91] examined the effect of an IBS SMS intervention on HRQOL using the Irritable Bowel Syndrome Quality of Life questionnaire (IBSQOL), a 30-item questionnaire. Compared to usual care, participants receiving the intervention demonstrated statistically significant improvements in QOL, increasing by 10.6 units, 12.8 units and 12.2 units at nine weeks, six and twelve-months, respectively. Changes persisted at 12-month follow-up (p<0.001) [91].

#### Physical, psychological or social functioning

Physical, mental or social functioning were measured in 25 RCTs [31, 43, 44, 47, 54, 56–58, 60–64, 68, 72–76, 79, 84–97, 100, 102, 108, 111–115, 117]. Psychological symptoms and social functioning using the CORE-OM scale [123] were measured in three studies [74, 75, 100]. Psychological functioning was measured using the Beck Depression Inventory (BDI) and Beck Depression Inventory-II (BDI-II) scale [124] in eight studies [72, 75, 84, 92–94, 97, 100, 108, 117]. Williams et al. [75] reported lower mean BDI-II scores in the intervention group at four months (2.6 to 7.9; mean difference 5.3 points, p<0.001). At twelve-month follow-up, there were also significantly higher proportions of participants achieving a 50% reduction in BDI-II in the intervention arm compared to control [75]. The Problem Areas in Diabetes Scale (PAID), a brief self-report scale [125], was used to evaluate diabetes-related distress. Sturt et al. [44] reported a reduction by 4.5 points in mean PAID scores at follow-up (95% CI: −8.1, −1.0), indicating lowered diabetes-related distress after a nurse-delivered intervention compared with control (p = 0.012), however this difference was considered a small effect [44]. Physical functioning was assessed with the SF-36PF scale [126] by Friedberg et al. [94, 108] evaluating a chronic fatigue self-management intervention. No significant changes in scores by time, treatment group, or diagnostic group were revealed (p>0.05) [94, 108].

#### Patient self-efficacy

Self-efficacy was assessed using a number of validated instruments including the General Self Efficacy Scale (GSES-12) [127], Diabetes Management Self Efficacy Scale (DMSE) [128] and the Arthritis Self Efficacy Scale (an eight item scale measuring patients’ perceived ability to perform specific behaviours aimed at controlling arthritis pain and disability) [129], the COPD self-efficacy scale (CSES) [130], among others. Self-management and patient enablement were measured by the Patient Enablement Instrument (PEI) [87]. Changes in perceived self-efficacy were reported in 14 studies [31, 44, 54, 57, 68, 69, 73, 76–78, 84, 87, 98, 99, 102, 104, 110, 111, 116]. Sturt et al. showed self-efficacy scores were 11.2 points higher on the DMSE (95% CI: 4.4, 18.0) in the intervention group compared with the control group following a structured intervention delivered by practice nurses in the UK (p = 0.0014) [44]. Broderick et al. [84] reported significant improvement in self-efficacy (F(3,232) = 10.59, p = 0.001) following a nurse-practitioner delivered intervention for OA patients, however this was not maintained at 12-month follow up (p = 0.158). Seven RCTs reported non-significant improvements in self-efficacy [54, 57, 59, 68, 69, 77, 78, 98, 102, 105, 110, 111, 116]. Bischoff et al. found no statistically significant changes in CSES scores at 24 months [69]. Smit et al. [77, 116] assessed self-efficacy in controlling depressive symptoms and preventing future episodes, using the Depression Self-Efficacy Scale (DSES) [131]. No statistically significant differences between groups were revealed at 12-month follow-up [77, 116]. Eikelenboom et al. reported no significant difference in PAM-13 scores (measure of patient activation [132]) between control and intervention arms at six-month follow-up [59, 105].

#### Self-management behaviours

Behaviours commonly measured were diet, physical activity, medication adherence and smoking. Five studies reported on level of physical activity [41, 59, 83, 88, 103, 105]. A range of measures included the International Physical Activity Questionnaire short form (IPAQ-SF) [133], Rapid Assessment of Physical Activity questionnaire (RAPA) [134] and The Physician-based Assessment and Counselling for Physical Activity (PACE) questionnaire [135]. No significant between group differences were reported for physical activity in 4 RCTs [41, 59, 65, 83, 103, 105]. There was evidence in one study to suggest self-reported exercise participation was higher 1-week post-intervention (p<0.001) however differences were no longer significant at seven-week follow-up [88]. Self-care activities within 7 days were measured in 4 RCTs [41, 52, 54, 60, 102, 103] using the Summary of Diabetes Self-Care Activities (SDSCA) questionnaire, a brief self-report instrument for measuring levels of self-management in diabetes (‘general diet’, ‘specific diet’, ‘physical exercise’, ‘foot care’ and ‘smoking’) [136]. Mehuys et al. reported significant improvements in self-management activities in the domains of ‘specific diet’ (+0.5 day/week, p = 0.008), ‘physical exercise’ (+0.4 day/week, p = 0.006), and ‘foot care’ (+1.0 day/week, p<0.001) for intervention patients. There were significant between-study group differences in the domains ‘physical exercise’ (p = 0.045) and ‘foot care’ (p<0.001), however the between-group difference for ‘specific diet’ were non-significant [52].

#### Disease knowledge

Nine studies reported disease knowledge as an outcome [52, 66–68, 71, 81, 82, 88, 93, 111]. Two RCTs [67, 68, 111], measured COPD disease knowledge using the Bristol COPD Knowledge Questionnaire (BCKQ) [137]. Hill et al. reported the results of the BCKQ for each domain in both groups. Compared with baseline measures, the total Bristol COPD knowledge Questionnaire score increased from 27.6 ± 8.7 to 36.5 ± 7.7 points (p<0.001) in the intervention group, and unchanged in the control group (29.6 ± 7.9 to 30.2 ± 7.2; p = 0.51) [67].

### Intervention components and theoretical underpinnings

Each of the studies described interventions including multiple core components (see S5 Table for full component breakdown). Providing knowledge about the condition or treatment (100%; n = 58), enhancing patients role in making lifestyle changes (71.9%; n = 41), development of a self-management or action plan (45.6%; n = 26), keeping logs of self-monitoring (43.9%; n = 25), strategies for psychological coping with conditions (43.9%; n = 25), enhancing problem-solving and/or decision-making skills (42.1%; n = 24) and medication adherence or management (36.8%; n = 21) were most prominently detected (Table 3). Interventions targeting heart disease, irritable bowel disease (IBD) and asthma reported the highest number of self-management components. Self-treatment through the use of an action plan, enhancing medication adherence and smoking cessation components were frequently seen in studies evaluating interventions targeting COPD. Similarly, SMS components targeting T2DM commonly included strategies to stimulate symptom monitoring, making positive lifestyle improvements with physical activity or dietary improvements. In contrast, interventions for depression included components focusing on patients’ role in managing stress, problem-solving and strategies for coping with conditions.

**Table 3:** Frequency of self-management components of included interventions.

Components	Number of studies in which this strategy is mentioned N (%)
Providing knowledge about condition or treatment	58 (100.0)
Stimulation of physical activity	27 (47.4)
Enhancing problem-solving and/ or decision-making skills	27 (47.4)
Self-treatment through use of self-management or action plan	26 (45.6)
Active stimulation of symptom monitoring	25 (43.9)
Emotional coping with condition or stress management	25 (43.9)
Enhancing dietary intake	24 (42.1)
Medication management or adherence	21 (36.8)
Encouraging use of other health services or support resources	13 (22.8)
Enhancing smoking cessation	13 (22.8)

Overall, sixteen studies explicitly reported a theoretical framework underpinning the intervention (28.1%; n = 16) including Cognitive Behavioural Theory (17.5%; n = 10) [58, 74, 75, 84, 90–94, 100], Social Cognitive Theory (3.5%; n = 2) [79, 104], Prochaska and DiClementes’ Transtheoretical model of the Stages of Change (3.5%; n = 2) [51, 55, 66, 82], Social Learning Theory (1.8%, n = 1) [44], Normalization Process Theory [31] and Implementation Intention Theory (1.8%; n = 1) [104]. Intervention fidelity was reported in 21 studies (27.6%; n = 16).

#### Training of primary care provider to deliver SMS

70.7% (n = 41) of studies included upskilling of HCPs to deliver the intervention. Training aimed at enhancing aspects of patient self-efficacy including mastery achievements, positive learning, adjustment to stress, verbal encouragement and outcome expectations. Intervention approaches were underpinned by the use of core communication skills to build trust and rapport in the patient-provider relationship, and as such providers were trained in areas including active listening, non-verbal communication, reflection, empathy and affirmation. Studies reported the provision of HCP resources to support self-management, e.g. written material or manuals, feedback on care reports, video demonstrations or case studies, and tools to assess patient support needs and priorities (PRISMS).

### Interventions reporting positive findings for clinical and humanistic measures

Thirteen RCTs targeting a range of conditions including asthma, T2DM, COPD, recurrent binge eating, chronic fatigue, major depression, low self-esteem, IBS and depression reported positive findings for all clinical and humanistic outcome measures (Table 4) [42, 66, 67, 72, 75, 80, 91, 92, 95, 100, 117].

**Table 4:** RCTs showing positive findings for all outcome measures.

	Transfer of information	Self-treatment through use of an action plan	Active stimulation of symptom monitoring	Stress or psychological management	Enhancing problem solving/ decision-making	Resource utilization	Enhancing physical activity	Enhancing dietary intake	Enhancing smoking cessation	Enhancing medication adherence
Barbanel et al. [80]	✓	✓	✓	✘	✓	✘	✘	✘	✓	✓
Chalder et al. [95]	✓	✘	✓	✘	✓	✘	✘	✘	✘	✘
Efraimsson et al. [66]	✓	✓	✘	✘	✓	✘	✓	✓	✓	✓
Ferrone et al. [71]	✓	✓	✘	✓	✓	✘	✓	✓	✓	✓
Fortin et al. [62, 63]	✓	✘	✘	✘	✘	✘	✓	✓	✓	✘
Goudswaard et al. [42]	✓	✘	✓	✘	✘	✘	✓	✓	✘	✓
Heitkemper et al. [91]	✓	✓	✓	✓	✓	✘	✘	✓	✘	✘
Hill et al. [67]	✓	✘	✘	✘	✘	✘	✓	✘	✓	✓
McLean et al. [82]	✓	✓	✓	✘	✓	✘	✘	✘	✘	✓
Striegel-Moore et al. [92, 117]	✓	✘	✓	✓	✓	✓	✘	✓	✘	✘
Waite et al. [100]	✓	✘	✘	✓	✓	✘	✘	✘	✘	✘
Watkins et al. [72]	✓	✘	✘	✓	✓	✘	✘	✘	✘	✘
Williams et al. [75]	✓	✘	✘	✓	✓	✘	✘	✘	✘	✘
**Total**	**13**	**5**	**6**	**6**	**7**	**1**	**5**	**6**	**5**	**6**

Summary: (**✓**) component present; (✘): component absent/ unclear/ not specified

A mean of five self-management components (SD 1.7) were included in effective interventions. Elements most frequently reported to enhance the patient’s role in self-management included information provision (100.0%; n = 13), enhancing problem-solving or decision-making skills (76.9%; n = 10), active stimulation of symptom monitoring (46.2%; n = 6), medication management or adherence (46,2%; n = 6), strategies for stress or psychological management of condition (46.2%; n = 6) or enhancing dietary intake (46.2%; n = 6). The total duration of interventions ranged from 4 to 52 weeks. Initial consultations were on average 62 minutes (SD 13.8). Follow-up was delivered face-to-face in 11 interventions (84.6%; n = 11), and two studies reported telephone follow up (15.4%; n = 2). Studies reported mean of five follow-up sessions (SD 3.6) on average, ranging from 1 to 12 sessions. Mean duration of follow up sessions were 57 minutes (SD 18.5). Individuals were provided self-help support materials or resources in majority of interventions (92.3%; n = 12). Accompanying patient materials provided in addition to face-to-face sessions included manuals, information or educational booklets to work through at home, personalized treatment or action plans, devices and diaries for self-monitoring, goal setting forms or individualized dietary plans. Six RCTs incorporated a theoretical underpinning in their intervention: cognitive behavioral theory (30.8%; n = 4) and Prochaska and DiClementes’ transtheoretical model of the stages of change (15.4%; n = 2). Five integrated cognitive behavioral therapy (CBT) into their intervention (38.5%; n = 5).

Barbanel et al. [80] and Goudswaard et al. [42] targeted asthma and T2DM respectively and produced positive improvements in clinical outcomes. The SMS intervention evaluated by Barbanel et al. [80] examined the impact of a self-management program delivered by community pharmacists on asthma control. Intervention participants received self-management support from the pharmacist with weekly telephone follow-up for 3 months. This included a review of inhaler technique, skills including monitoring of peak flow, and a personalized action plan for worsening symptoms or exacerbations. Symptom scores improved in the intervention group and marginally worsened in the control group to 20.3 (4.2) and 28.1 (3.5), respectively (p<0.001; adjusted difference = 7.0 (95% CI: 4.4, 9.5). Goudswaard et al. [42] evaluated long-term effects of nurse-delivered self-management education in type 2 diabetics. The intervention focused on medication adherence, enhancing physical exercise, dietary intake and self-monitoring blood glucose at home. Six sessions were provided at intervals of 3–6 weeks, resulting in contact time of approximately 2.5 hours with HCPs over 6 months. HbA1c levels improved from 8.2% to 7.2% in the intervention group and 8.8% to 8.4% in usual care at 6 weeks, however this result was not sustained at 18 months [42].

Efraimsson et al. [66] examined effects of nurse-led COPD intervention. Patients received education on self-care ability to cope with disease and treatment. Patients were scheduled for two visits with nurses lasting 60 minutes during a 5-month period. A statistically significant increase was noted in the intervention group on QOL, the proportion of patients who ceased smoking, and patients’ knowledge about COPD at 3–5 month follow up, compared with usual care. Heitkemper et al. [91] examined an intervention delivered to women with IBS. Women in the intervention received eight weekly 1-hour individual sessions. The intervention included education, dietary counselling, symptom monitoring, relaxation training and cognitive-behavioral strategies including anger management, cognitive restructuring, assertiveness and social skills training [91]. Hill et al. [67] examined an intervention in people with COPD. Intervention participants attended two one-to-one 60-minute sessions, focusing on enhancing self-efficacy. Sessions were accompanied by a written manual adapted from the "Living Well with COPD" program. COPD knowledge increased from 27.6 (+/- 8.7) to 36.5 (+/- 7.7) in the intervention group, which was greater than any difference seen in the control group. Waite et al. [100] examined an individualized intervention for patients with low self-esteem. This included goal setting, learning skills to re-evaluate anxious and self-critical thoughts and beliefs through cognitive techniques. All participants were given a three-part self-help workbook in addition to individual treatment sessions. The intervention showed significantly better functioning than control on measures of overall functioning and depression and had fewer psychiatric diagnoses at the end of treatment. All treatment gains were maintained at follow-up assessment. Williams et al. [75] evaluated a guided self-help intervention for depression in primary care. The first appointment focused on an introduction to the use of the self-help materials. Three additional face-to-face support sessions of approximately 40 minutes were provided on a weekly or fortnightly basis. Mean Beck Depression Inventory (BDI-II) scores were lower in the intervention group at 4 months by 5.3 points, compared with control (2.6 to 7.9, p = 0.001). There were also significantly higher proportions of intervention participants achieving a 50% reduction in BDI-II scores at 4 and 12 months.

McLean et al. [82] involved a pharmacist-delivered intervention for asthma self-management. The intervention involved education surrounding the basic concepts of disease, medications, trigger identification and avoidance, and an asthma action plan. Patients were taught to use a peak flow meter, spacer devices, calendars/diaries were provided and asked to record peak expiratory flow rates (PEFRs) regularly for the course of the study period. Patients received appointments of approximately one hour in length with a pharmacist in a private counselling area every two to three weeks for at least three appointments, and then follow-up appointments at least quarterly for 12 months [82]. Symptom scores decreased by 50% (p<0.05) and peak flow readings increased by 11% (p = 0.0002) for intervention patients, compared to those receiving usual care. Chalder et al. [95] evaluated the efficacy of a self‐help booklet and advice delivered by a nurse in reducing chronic fatigue in adult patients. The intervention reiterated self-monitoring and maintaining symptom diaries. Basic cognitive techniques such as identifying and challenging unhelpful thoughts were also introduced. The self‐help group showed significantly greater improvements in fatigue (p = 0.01) and psychological distress (p<0.01) than controls. Striegel-Moore et al. [92, 117] evaluated cognitive behavioural guided self-help for the treatment of recurrent binge eating. Intervention participants received 8 sessions over 12 weeks. The primary focus of this intervention was on developing a regular pattern of moderate eating using self-monitoring and problem-solving. The main outcome, abstinence from binge eating differed significantly between the groups: the initial improvement in abstinence from baseline was greater for the intervention group than usual care (p<0.001). Watkins et al. [72] evaluated guided self-help concreteness training as an intervention for major depression. During the initial session of the self-help intervention, psycho-education and training exercises were provided. During the follow-up telephone sessions, feedback, guidance and encouragement was provided to ensure accurate use of exercises, and progress monitored. The intervention resulted in significantly fewer depressive symptoms post-treatment, relative to treatment as usual (ITT, p = 0.006, effect size d for change in Hamilton Rating Scale for Depression (HAMD) = 0.76; PP, p<0.0001, d = 1.06).

### Quality risk of bias assessment of individual studies

**Fig 2:**
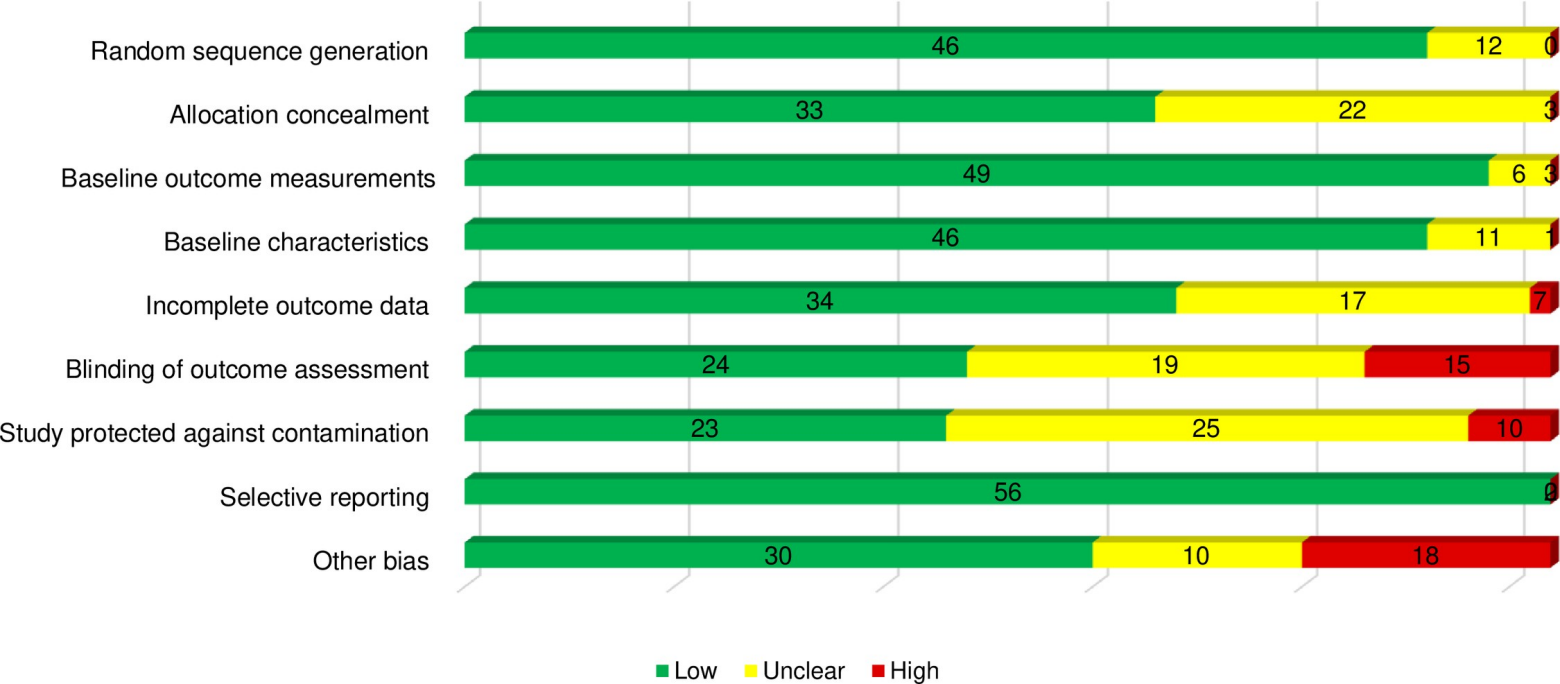
Risk of bias graph.

The overall methodological quality was considered high (lower risk of bias) in 41.4% of studies (n = 24 RCTs), and of medium quality in 58.6% of studies (n = 34 RCTs). The domains considered lowest risk of bias were selective reporting (96.6%; n = 56), baseline outcome measures (84.5%; n = 49), random sequence generation (79.3%; n = 46) and baseline characteristics (79.3%; n = 46). The domains with higher risk of bias were ‘blinding of outcome assessment’ (25.9% of studies; n = 15). Reporting bias was judged low for more than 95% of studies. Half of studies (51.7%; n = 30) presented low risk for the domain ‘other bias’. Reasons for other risk of bias included not meeting recruitment targets for assumed power. Fig 2 shows aggregate appraisal of risk of bias of included studies and visual representation of each domain.

## Discussion

This systematic review has synthesized evidence from 58 randomized controlled trials examining the effectiveness of primary HCP delivered self-management support interventions for adult patients, with any condition, compared to usual standard of care. We describe effective SMS interventions and have highlighted their active elements, identified trends in combinations of intervention strategies, range of outcomes measured and the magnitude of effect size. This review demonstrates that SMS interventions delivered face-to-face by primary HCPs, which are multicomponent and tailored to explicitly enhance patient self-management skill set can lead to improvements in clinical and humanistic outcomes. The various tools and strategies that provide a structure to interventions delivered face-to-face include adapting interventions according to patients’ readiness to change, action planning and goal setting by collaboratively breaking down individual health goals into small achievable actions. The effectiveness of multicomponent SMS interventions is not surprising. But it raises the question of how to focus efforts on the best combination of active components within interventions. The variation in context, outcome measures, training methodology used across the 58 studies, in addition to the high degree of autonomy given to providers, deem the evaluation of SMS interventions more difficult.

Ninety-three different outcome measures were adopted to demonstrate evaluated impact of the various interventions and presumably were selected to reflect expected outcomes or processes of self-management. These include different measures of health-related quality of life, overall functioning, self-efficacy, health behaviours, disease knowledge, symptoms and disease control. Disease specific clinical indicators were mostly included as primary outcomes, and QoL indicators generally served as secondary or ancillary outcomes to primary outcome criteria. Generic HRQOL measures varied across different types of diseases, interventions and groups (i.e. EQ5D, SF-12), and specific HRQOL disease measures were also utilized. (i.e. IBSQOL questionnaire was used to measure changes in HRQOL for IBS patients). Further examination of studies producing positive improvements in HRQOL revealed use of disease specific measures (i.e. Ferrone et al. [71] reported positive changes in HRQOL using the Clinical COPD Questionnaire (CCQ)—a 10-item, health-related quality of life questionnaire). Interestingly, studies using more generic HRQOL measures (i.e. EQ5D, SF scales) mostly reported insignificant differences in their interventions. S4 Table provides a summary of the various instruments used in studies.

Our findings reveal a structured patient-provider exchange is required in primary care (including a one-on-one patient-provider consultation, ongoing follow up and provision of self-help materials). A systematic and tailored patient-primary care provider exchange is needed to provide individuals with the portfolio of techniques and tools to effectively self-manage. Various combinations of strategies were used to achieve this and adapted to the individuals’ condition, health literacy, skills and confidence in managing their own health. Strategies containing several interacting components and varying dimensions of complexity produce favourable effects when tailored to the individual. No one intervention solution is suitable for all patient groups and the selection of combinations of strategies should support patients’ needs relevant to both primary care and HCP. The strategy of enhancing the patient’s decision-making skills or ability to problem-solve was reported in the highest percentage of studies (53.8%) with positive results, after knowledge acquisition. Active stimulation of symptom monitoring (46.2%) and having specific, clear and accepted treatment or healthcare goals was also commonly identified. This involved setting measurable, clear and accepted treatment or healthcare goals on a per patient basis with a specific action or self-management plan detailing these. Tailored, written information and care plans that are mutually agreed upon have previously been identified as helpful [138]. Strategies to improve responsibility in medication adherence and lifestyle choices were also reported within effective interventions.

Interestingly, strategies for stress or psychological coping of conditions (46.2%) were commonly identified in effective interventions. Changing the patient’s cognitive approach to their illness was commonly incorporated into the intervention to deal with the physical and emotional symptoms resulting from a chronic illness. Effective interventions integrated cognitive behavioral therapy (CBT) into the intervention in 40% of studies. Multiple cognitive strategies were raised, such as identifying and challenging unhelpful thoughts [95], relaxation training and cognitive-behavioral strategies including anger management, cognitive restructuring, assertiveness and social skills training [91]. A 2014 systematic review of qualitative literature identified patients often express difficulties in dealing with the physical and emotional symptoms of their chronic conditions [138]. As such, undesirable physical and emotional symptoms and impaired physical functioning can directly prevent patients from carrying out normal daily activities, including tasks required to appropriately and successfully self-manage [139–141]. Self-management of chronic conditions should therefore be examined not only from the clinical perspective, but also the patient perspective with a focus on humanistic outcomes. Importantly, the theory of SMS drawn for effective studies included Cognitive Behavioral Theory and Prochaska and DiClementes’ transtheoretical model of the stages of change. Follow-up by HCPs included tailored feedback, monitoring of progress with respect to patient set healthcare goals, or honing problem-solving and decision-making skills. Self-help tools and assistance with locating resources were commonly provided during the patient-provider exchange.

The scope of the terms ‘self-management’, ‘self-management support’ and ‘self-management support interventions’ in literature and the large heterogeneity in terminology has repeatedly been highlighted in previous systematic reviews and meta-analyses [27, 142–144]. This is a key limitation, as very broad or very narrow definitions of what constitutes “self-management support” have been applied. Lorig and Holman [10] previously underlined the need to explore interventions beyond the label of self-management to define if interventions actually address the necessary support strategies required to change behaviour. Subtle variations in self-management definitions can result in substantial differences in selected studies. Using Jonkman’s operational definition [35] to define our interventions has shown highly important in distinguishing self-management interventions from other types of interventions (ie. patient education or disease management) without being too restrictive. The definition clearly defines the elements or strategies that constitute a self-management support intervention, with the pivotal objective of changing behaviour. This has guided the selection of studies on which our review conclusions have been based.

A notable gap identified in the literature was a lack of focus on multimorbidity. This is understood to pose challenges for self-management, as many individuals have more than one health condition [138]. The effects of multimorbidity on a person are not always linear. Interestingly enough, some studies have found that patients with multimorbidity consider themselves better at self-management because they had already developed skills such as self-monitoring and self-advocacy [145].

**Fig 3:**
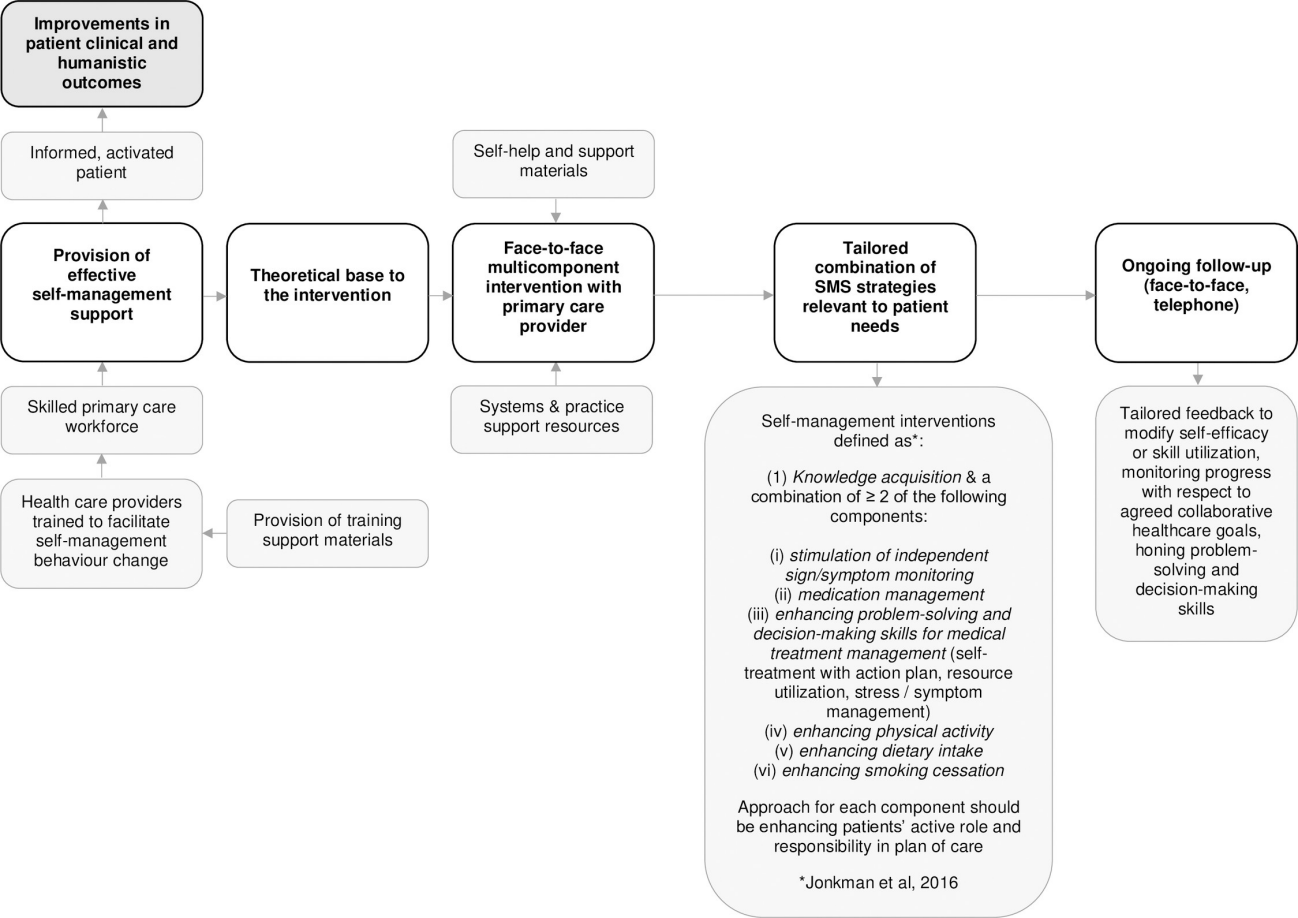
Elements for a practical approach for HCPs in supporting face-to-face SMS multi-component strategies, individually tailored for patients in primary care. Modelled on the definition of self-management interventions by Jonkman et al. 2016 [35].

In acknowledging that SMS is a multidimensional topic, we aimed to create a broader picture of the landscape of SMS in primary care. This was achieved by evaluating the patterns of intervention components comprehensively across all conditions, by not limiting our research to a clinical condition, or specific intervention strategies. Although including different clinical conditions in the review may be considered as a drawback due the potential heterogeneity induced, in our research, there was a clear distinction of strategies across the conditions studied. Findings from this review add further detail to this body of knowledge, while providing HCPs with a number of evidence-based strategies that can be utilized in practice. These findings pave the way to explore further SMS strategies targeting patient’s behaviour change, effective patterns of strategies, and develop a more evidence-based model for optimum SMS service design. Primary care providers (e.g. general practitioners, nurses, pharmacists) can play a foundational role in supporting patient self-management, especially for people with multiple chronic conditions. Fig 3 sets the foundation for an evidence-based SMS primary care model for face-to-face interventions, allowing for a more efficient and effective process to evaluate and implement SMS interventions in primary care.

For this collaborative partnership approach to be more widely applied, there should be a strong focus on upskilling primary care providers to deliver SMS strategies in health care, which are both integrated and coordinated to improve the patient-provider encounter in practice [5]. The total duration of the intervention and the correlation of intervention duration with the number of strategies delivered are important aspects when considering the sustainability within primary care. Policy and funding alignment will also be a major determinant for future sustainability. Therefore, we must determine where the best compromise in SMS interventions lie for cost-effective and resource-limited approaches. Future high-quality evaluations of consistent interventions will be of value to practitioners, policy-makers and researchers in terms of collecting clinical, humanistic and economic outcome measures to generate a robust evidence base of primary care providers impact in the area. This will also allow determination of ineffective combinations of strategies.

Future research efforts should continue to expand on this landscape to (1) examine the patterns of strategies within effective multicomponent interventions for various conditions; (2) examine the weighting of each strategy (ie. determine intervention components which are more or less effective) within effective multicomponent interventions; (3) determine if certain types of patient populations could be targeted most effectively by certain combinations of strategies; (4) develop a core SMS outcome set in primary care; (5) examine the patient’s ability to self-manage over time as well as aiming to achieve the goal of long-term sustainability for improved self-management; and (6) determine training requirements for the upskilling of health care providers for sustained patient behaviour change.

Furthermore, sustainability of improved SMS first requires an understanding of the implementation of SMS enhancing interventions [146]. Sustainability can be challenging if not embedded into everyday clinical practice [31], and achieving the potential of primary care as a platform to effectively deliver SMS and achieve the stated outcomes means overcoming known barriers, such as limited time, skills and confidence among health professionals [31, 147]. We know changes in health care professional practice requires exhaustive planning and testing to increase the probability that they are successfully and sustainably implemented. The adoption of Intervention Mapping has been widely used in health care settings to plan changes in the behaviour and practice of health care professionals, and should be applied to ensure SMS interventions are both effective and successfully implemented in practice [148].

There are limitations to this review. A number of studies did not report sufficient detail to their interventions which hampered the assessment of possible effective combinations of strategies being evaluated. The methodological quality domains of the included trials were in a lot of cases unclear, with a lack of poor description of the study methodology and intervention fidelity in evaluations. This was mitigated by contacting authors for further relevant information, searching for study protocols or further examining supplementary data online. With the growing recognition of the importance of assessing treatment fidelity in multicomponent interventions [149–151] (ie. compliance to treatment protocols by HCPs, or compliance to treatment by patients), it is important to note most trials (72%) did not include this in their design and few provided data on treatment fidelity to the intervention. Only 38% of effective interventions reported an assessment of intervention fidelity. The methodological quality domains of the included trials were in a lot of cases unclear. Four high-quality studies provided positive evidence that SMS interventions delivered in primary care dominate usual standard of care, by improving patients’ clinical outcomes, HRQOL or psychological functioning [72, 91, 92, 100]. Similar trends have been found in existing literature in several contexts that self-management is essential to optimizing clinical and humanistic outcomes for patients with chronic conditions [13, 15, 18, 152–155].

Although multiple databases were extensively searched using clear, specific and appropriate terms, the search may not have yielded all published relevant studies given the ambiguity of what constitutes “self-management support” and the variation in terminology for “self-management” identified in the literature. Unsurprisingly, with the rising burden of chronic disease, the nomenclature of “self-management” has become more prevalent in both published and grey literature. We recognize the use of different search terms and definitions to guide the development of the search strategy may lead to variation in the identification of studies, and affect a review's conclusions. This is identified as a limitation of our review. Search terms were sourced from previous systematic reviews, primary studies and grey literature. Our search included general terms for “self-management” and was not limited to specific illnesses or outcomes.

Systematic reviews are at risk for bias from a number of sources [156]. We sought to reduce potential sources of bias within the inclusion and synthesis of studies. One of our main goals was developing inclusion criteria to minimize ambiguity and reduce bias in study selection decisions. We have defined our inclusion and exclusion criteria by PICO clearly and have documented and reported all decisions made in the study selection process for transparency. Since we restricted our review to face-to-face interventions, there may be other SMS interventions that may be effective that are not covered by this review. We decided to categorize the comparator as usual standard of care and understand the definition of usual standard of care may vary by country or healthcare system.

## Conclusion

In conclusion, this review highlights core components of successful interventions showing positive clinical and/or humanistic outcomes. Whilst it was difficult to directly correlate individual strategies to outcomes and effectiveness, there was a clear distinction of strategies across the conditions studied. This review provides encouraging groundwork for the design and evaluation of practical strategies for evidence-based practice and the construction of self-management support processes in primary healthcare practice. This review may assist in determining the breadth and focus of the support primary care professionals provide. Application of a theoretical perspective provides a strong base for the development of SMS interventions. The developed model sets the foundation for the design and evaluation of practical strategies for the construct of self-management support in primary healthcare practice. These results may be used to justify additional research investigating self-management interventions delivered in the primary care setting. In response, primary care providers can begin to deeply reflect on current practice and become involved in a dialogue to improve self-management support. Critically, these results should stimulate informed discussion for the future delivery of self-management support in primary care and the requirements for upskilling healthcare providers to effectively support patients in this collaborative process.

## Supporting information

S1 Data Database. (XLS)

S1 Table Search strategy. (PDF)

S2 Table PRISMA checklist. (PDF)

S3 Table Descriptive characteristics of included studies. (PDF)

S4 Table Summary of findings and extracted outcomes. (PDF)

S5 Table Mapping of intervention components. (PDF)
