# Supplementary material for: Helping patients help themselves: A systematic review of self-management support strategies in primary health care practice
Source: PLoS One. 2019 Aug 1;14(8):e0220116. doi: 10.1371/journal.pone.0220116 (PMC6675068; doi:10.1371/journal.pone.0220116)
Supplement: S1 Table — (PDF) [file pone.0220116.s002.pdf]

S1 Table. Search strategy

Database	Search query
**PubMed**	("Self Efficacy"[Mesh] OR "Self Concept"[Mesh] OR "Self Care"[Mesh] OR "Self Efficacy"[Title/Abstract] OR "Self-Management"[Title/Abstract] OR "Self Care" [Title/Abstract] OR "Self Report"[Title/Abstract] OR "Self Medication"[Title/Abstract] OR "Self Concept"[Title/Abstract])<br>AND<br>("Primary Health Care"[Mesh] OR "Health Personnel"[Mesh] OR "Collaborative Care"[Title/Abstract] OR "Primary Care"[Title/Abstract] OR "Primary Health Care"[Title/Abstract] OR "Community Care"[Title/Abstract] OR "Primary Medical Care"[Title/Abstract] OR "Community Health Services" [Mesh])<br>AND<br>("Cluster Randomised"[Title/Abstract] OR "Controlled Trial"[Title/Abstract] OR "Randomized Controlled Trial"[Title/Abstract] OR "Randomised Controlled Trial"[Title/Abstract] OR "Clinical Trial"[Title/Abstract] OR "Clinical Study"[Title/Abstract] OR "Intervention Study"[Title/Abstract] OR "Randomized"[Title/Abstract] OR "Randomised"[Title/Abstract])<br>AND<br>("Patient Education as Topic"[Mesh] OR "Cognitive Therapy"[Mesh] OR "Patient Care Planning"[Mesh] OR "intervention" [Title/Abstract])
**Scopus**	KEY ("Community Care" OR "Patient Care" OR "Pharmacy" OR "General Practice" OR "Primary Medical Care")<br>AND<br>KEY ("Self Medication" OR "Self Concept" OR "Self Care" OR "Self-Management" OR "Self Report")<br>AND<br>KEY ("Health Program" OR "Patient Education" OR "Health Education" OR "Health program" OR "Health Service" OR "Health Care Management")<br>AND<br>KEY ("Randomized Controlled Trial" OR "Clinical Trial")
**Web of Science**	TS=("Self Efficacy" OR "Self-Management" OR "Self Care" OR "Self Report" OR "Self Medication" OR "Self Concept")<br>AND<br>TS=("Collaborative Care" OR "Primary Care" OR "Primary Health Care" OR "Community Care" OR "Primary Medical Care")<br>AND<br>TS =("Cluster Randomised" OR "Controlled Trial" OR "Randomized Controlled Trial" OR "Randomised Controlled Trial" OR "Clinical Trial" OR "Clinical Study" OR "Intervention Study" OR "Randomized" OR "Randomised"<br>AND<br>TS=("Patient Education" OR "Cognitive Therapy" OR "Patient Care Planning" OR "Intervention")
