# Supplementary material for: Helping patients help themselves: A systematic review of self-management support strategies in primary health care practice
Source: PLoS One. 2019 Aug 1;14(8):e0220116. doi: 10.1371/journal.pone.0220116 (PMC6675068; doi:10.1371/journal.pone.0220116)
Supplement: S3 Table — (PDF) [file pone.0220116.s004.pdf]

S3 Table. Descriptive characteristics of included studies

Author	Title, year, country	Study design, sample size	Condition	Intervention and control-group description	Healthcare professional	Study follow up (mode, time point)	Outcomes assessed
**Adachi et al. (1)**	Effects of lifestyle education program for type 2 diabetes patients in clinics: a cluster randomized controlled trial<br>2013, Japan	c-RCT<br>20 practices<br>193 participants<br>(IG: 100; CG: 93)	Type 2 diabetes (T2DM)	IG: The intervention group received structured individual-based lifestyle education that encouraged the reduction in energy intake at dinner and an increase in vegetable intake at breakfast and lunch. Support for self-management of glycemic control, such as by diet, exercise, and stress management, was provided in 3 or 4 sessions with trained registered dieticians during the study period. The program for the IG was structured in four steps: "Basic information on glycemic control", "Actions for glycemic control", "Daily activities for glycemic control", and "Management of stress for glycemic control". An assessment sheet, which was developed by consulting evidence-based practice guidelines for treatment of diabetes in Japan, was used. Patients decided on one or two short-term goals for glycemic control to be achieved in the next month based on the results of the FFQW82 and advice by registered dieticians. Sedentary participants were encouraged to increase basal physical activity. A gradual increase in physical activity was recommended.<br>CG: Usual care	Dietician	Face-to-face<br>3 or 4 sessions delivered in 6 months	Clinical outcomes:<br>Change in HbA1c levels<br>Fasting plasma glucose<br>Lipid profile<br>Blood pressure<br>Body mass index<br>Energy<br>Nutrient intakes<br>Humanistic outcomes:<br>NA
**Banasiak et al. (2)**	Guided self-help for bulimia nervosa in primary care: a randomized controlled trial<br>2005, Australia	RCT<br>109 participants<br>(IG: 54; CG: 55)	Bulimia Nervosa	IG: The intervention group received direction and support from a general practitioner (GP) over a 17-week period while working through a manual by Cooper (1995), 'Bulimia Nervosa and Binge-Eating: A Guide to Recovery'. Part 1 presents psychoeducational information about bulimia nervosa and Part 2 presents a six step, sequential, self-treatment program which offers cognitive behavioral strategies and advice to assist patients to overcome their eating problem. Each participant	General practitioner	Face-to-face<br>Weekly for the first 4 weeks, fortnightly for the following 6 weeks and then every 3 weeks for the remaining 6	Clinical outcomes:<br>Frequency over the past 28 days of episodes of: objective binge eating, subjective binge eating, vomiting, laxative, diuretic and enema misuse

				received an initial 30 to 60-minute session with the GP, provided the manual and an outline of guided self-help, treatment rationale and goals and advised to work through the program at their own pace. The GP provided 9 treatment sessions in the course of normal clinical practice lasting 20-30 minutes each. Guidance sessions were weekly for the first 4 weeks, fortnightly for the following 6 weeks and then every 3 weeks for the remaining 6 weeks. Each session followed the general format (1) assessing and monitoring progress by reviewing homework (2) discussing and resolving identified difficulties (3) jointly setting homework.<br>CG: Delayed treatment control		weeks (total 9 sessions)	Body dissatisfaction<br>Body size through direct measurements of weight and height<br>Humanistic outcomes:<br>Psychological functioning<br>General functioning<br>Attitudes towards treatment
**Barbanel et al. (3)**	Can a self-management program delivered by a community pharmacist improve asthma control? A randomized trial<br>2003, UK	RCT<br>24 participants<br>(IG: 12; CG: 12)	Asthma	IG: Participants received a review of their inhaler technique and personal education from the pharmacist addressing the following topics: basic pathophysiology of asthma; recognition and avoidance of triggers; inhaler technique; self-management skills including monitoring of peak flow or symptoms; action in response to worsening symptoms; how to access emergency care appropriately; smoking cessation if relevant. Participants also received written personalized credit card self-management plans and educational leaflets. Self-management decision-making was based on peak expiratory flow readings if the participant could use and interpret readings from a peak flow meter; otherwise advice was based on symptoms. For self-management plans, the instructions were for patients to: 1. Double their inhaled corticosteroid dose if their peak expiratory flow rate (PEFR) was 70-80% of best or they were waking at night with symptoms 2. Contact their doctor to arrange a course of oral corticosteroid treatment if their PEFR was 50-70% or breathlessness was increasing 3. Call their doctor urgently if their PEFR was below 50% of best or symptoms continued to worsen.	Pharmacist	Telephone<br>Weekly for 12 weeks	Clinical outcomes:<br>Asthma symptoms (using the North of England asthma symptoms scale)

				CG: Usual care			
**Barley et al. (4)**	The UPBEAT nurse-delivered personalized care intervention for people with coronary heart disease who report current chest pain and depression: a randomized controlled pilot study<br>2014, UK	RCT<br>81 participants<br>(IG: 41; CG: 40)	Coronary Heart Disease<br>Chest pain & Depression	IG: A nurse conducted a standardized, face-to-face, bio-psychosocial assessment (including physical and mental health, difficulties with current treatment regimens, problems with daily activities and social problems). Patients were helped to identify up to three problems which they consider contribute to their depression and wanted to address. The nurse-case managers provided information, signposted patients to existing resources (e.g. leisure centers, social clubs, Improving Access to Psychological Therapy (IAPT) services) and use evidence-based behaviour change techniques to help patients set and achieve goals. The underlying intention of the intervention was to increase the patient's self-efficacy to achieve desired goals (as opposed to goals determined by others such as symptom management or reduction of cardiac risk factors). Details of the assessment and action plan are recorded in a 'personalized health plan', which the patient held.<br>CG: Treatment as usual	Nurse	Telephone<br>Weekly then variable according to patients need	Clinical outcomes:<br>Mood<br>Chest pain<br>Humanistic outcomes:<br>Functional status<br>Wellbeing and psychological process variables
**Bartels et al. (5)**	Integrated IMR for Psychiatric and General Medical Illness for Adults Aged 50 or Older with Serious Mental Illness<br>2014, United States	RCT<br>71 participants<br>(IG: 36; CG: 35)	Schizophrenia spectrum, bipolar disorder, or major depression PLUS diabetes, chronic obstructive pulmonary disease (COPD), congestive heart failure, ischemic heart disease, hypertension, hyperlipidemia,	IG: The I-IMR program integrated components of conventional IMR related to psychiatric illness self-management with strategies for self-management of general medical illness. The psychiatric component included psychoeducation about illness and treatment, cognitive-behavioral approaches to increase medication adherence, training in relapse prevention, instruction about coping skills to manage persistent symptoms, and social skills training. The general medical illness component consisted of an individually tailored curriculum that applied the same skills and strategies for self-management of psychiatric illness to the self-management of general medical illness. In addition, a nurse health care manager facilitated coordination of necessary preventive and ongoing health care. A primary care nurse was embedded one day per week at each mental health centre to coordinate health care	Social worker<br>Nurse	Face-to-face<br>Weekly (social worker) for 8 months<br>Fortnightly (nurse) for 8 months	Clinical outcomes:<br>NA<br>Humanistic outcomes:<br>Self-management of psychiatric and general medical illness

			or osteoarthritis	appointments, medication adjustments, and transfer of information and to provide counselling on self-management and lifestyle changes for chronic health conditions. Participants met with the nurse health care manager twice per month to discuss progress and obstacles in meeting general medical and mental health goals.<br>CG: Usual care			
**Bischoff et al. (6)**	Comprehensive self-management and routine monitoring in chronic obstructive pulmonary disease patients in general practice: randomized controlled trial.<br>2012, The Netherlands	RCT<br>165 participants<br>(IG (1): 55; IG (2): 55; CG: 55)	COPD	IG: A comprehensive self-management program as an adjunct to usual care, consisting of four tailored sessions with ongoing telephone support by a practice nurse; routine monitoring as an adjunct to usual care, consisting of 2-4 structured consultations a year with a practice nurse; or usual care alone (contacts with the general practitioner at the patients' own initiative). Patients in the self-management group received a translated and modified version of the Canadian self-management program "Living well with COPD." The self-management program consisted of paper modules and a written exacerbation action plan. Topics covered in the modules were COPD disease knowledge, respiratory drugs, breathing techniques, managing exacerbations, maintaining a healthy lifestyle, managing stress and anxiety (optional), and home exercise (optional). The individualized written exacerbation action plan covered early recognition of and prompt action in the course of an exacerbation. Actions included increase in bronchodilator use; initiation of standing prescriptions for prednisolone, antibiotics (if applicable), or both; or contacting the practice nurse or general practitioner. The practice nurse of each participating practice acted as case manager and applied the program to the individual patient in two to four sessions of approximately one hour each, scheduled in four to six consecutive weeks. The sessions took place in the general practice. The number of sessions depended on the patient's needs, but it was at least two. Subsequently, the nurse called	Nurse	Face-to-face<br>60 minutes; 2-4 sessions over 4-6 weeks<br>Telephone<br>6 sessions	Clinical outcomes:<br>Chronic respiratory questionnaire domain scores Frequency and patients' management of exacerbations<br>Humanistic outcomes:<br>Change in COPD specific quality of life (QOL) (Chronic respiratory questionnaire total score) Self-efficacy (COPD self-efficacy scale)

				each patient six times during the rest of the study period to reinforce self-management skills. The nurse was available for advice during business hours. Before the study, all nurses were trained in how to apply the self-management program. In addition, all nurses were observed at least once by a respiratory nurse who was a member of the study group and experienced in the self-management program. The respiratory nurse also coached the practice nurses by using a message board on a secured web-based application during the rest of the follow-up.<br><br>CG: Usual care			
**Broderick et al. (7)**	Nurse practitioners can effectively deliver pain coping skills training to osteoarthritis patients with chronic pain: A randomized, controlled trial<br><br>2014, United States	RCT<br><br>256 participants<br><br>(IG: 129; CG: 127)	Osteoarthritis	IG: Patients in the Pain Coping Skills Training (PCST) treatment condition received 10 sessions of individual PCST, which was designed to promote the use of cognitive-behavioral pain management coping skills. PCST interventions teach patients cognitive and behavioral skills to manage their pain and enhance their perception of pain control. Four broad coping skills were taught across the ten 30- to 45-minute sessions: relaxation response, attention diversion techniques, altering activity and rest patterns as a way of increasing activity level, and reducing negative pain-related thoughts and emotions. The sessions were outlined in detail in a treatment manual and followed a format of review of home practice assigned at the last session, instruction in a new coping skill, guided practice in that skill, and a home practice assignment. Homework assignments are an integral component of PCST, followed by review and problem-solving in the subsequent session. Consistent with the goal of testing the effectiveness of nurse practitioners (NPs) delivering PCST in the patients' doctors' offices, all treatment sessions were conducted in the clinics or by telephone (phone sessions). Up to 4 sessions could be conducted via telephone with some discretion on the part of the NP and patient. The first 3 sessions and the last session had to be conducted in person. Patients were	Nurse practitioner	Face-to-face<br><br>Weekly (total 10 sessions)<br><br>Telephone<br><br>Up to 4 sessions	Clinical outcomes:<br><br>Pain intensity<br>Fatigue<br>Use of pain medication<br><br>Humanistic outcomes:<br><br>Physical functioning<br>Psychological distress<br>Self-efficacy<br>Catastrophizing<br>Use of coping strategies<br>Health-related quality of life (HRQOL)<br>Social functioning<br>Health satisfaction

				provided with a treatment binder divided into sections for each session. These sections included handouts and logs to record home practice of the skill and reviewed by the NP at each session. Treatment sessions with a patient were stopped if they were not completed within 20 weeks of randomization.<br><br>CG: Usual care			
**Browning et al. (8, 9)**	Management of type 2 diabetes in China: The Happy Life Club, a pragmatic cluster randomized controlled trial using health coaches<br><br>2016, China	c-RCT<br><br>41 practices<br><br>668 participants<br><br>(IG: 372; CG: 296)	T2DM	IG: Intervention group participants received a combination of telephone and face-to-face health coaching in addition to usual care. Health coaches aimed to assist participants in achieving the treatment targets as outlined in the Chinese Guideline for Diabetes Prevention and Management, with the primary goal of treatment of HbA1c of less than 7.0%. An intervention manual that utilized existing local guidelines and recommendations (e.g. Dietary Guidelines for Chinese Residents) was used to guide health coaches. The initial step in each health coaching session was to set the agenda for the session with the participant. This was achieved by asking the participant to identify the most productive place to start the conversation, that is, 'What would be helpful to talk about today?' Once the participant identified a key issue for discussion, health coaches utilized their complex set of motivational interviewing (MI) skills by assessing current behaviour in relation to the issue and determined where the participant was in the change process. Health coaches then guided the conversation with the ultimate aim of strengthening the participants' own motivation and commitment for change. By stepping out of the expert role into a more guiding, collaborative role, health coaches engaged the person in the process of making significant, lasting changes in their own life.<br><br>CG: Usual care	General practitioner<br><br>Nurse<br><br>Psychologist	Face-to-face<br><br>2 sessions monthly for 3 months, then 1 session monthly until 12 months<br><br>Telephone<br><br>2 calls monthly for 3 months, then 1 call monthly until 12 months	Clinical outcomes:<br><br>HbA1c<br>Systolic and diastolic blood pressure<br>Weight<br>Body mass index<br>Waist and hip circumference<br>Fasting plasma glucose Total cholesterol; triglyceride; high-density lipoprotein; low-density lipoprotein<br><br>Humanistic outcomes:<br><br>Psychological distress<br>QOL<br>Diabetes self-care activities<br>Diabetes management self-efficacy
**Chalder et al.**<br><br>**(10)**	Self-help treatment of chronic fatigue in the community: A	RCT	Chronic fatigue	IG: The experimental group was given a self-help booklet. The nurse spent between 10 and 15 minutes discussing its contents, focusing on information that	Research Nurse	Face-to-face	Clinical outcomes:<br><br>Fatigue symptoms

	randomized controlled trial.<br>1997, United Kingdom	150 participants<br>(IG: 70; CG: 80)		seemed pertinent to the individual. All participants were asked to return to the practice for follow-up three months after recruitment. The booklet was divided into three sections. Part 1 provided general information about fatigue and outlined different factors which contribute to both onset and maintenance of fatigue. Part 2 described the importance of self-monitoring and how diary-keeping helps to build a clear picture of fatigue in relation to activities. Part 3, the largest section, described a variety of cognitive and behavioral techniques for overcoming fatigue. The booklet indicated that fatigue may be associated both with doing too much and with doing too little, and the emphasis was on achieving a balance between the two. As sleep problems are common in people who complain of fatigue a section on how to improve sleep was included. Basic cognitive techniques such as identifying and challenging unhelpful thoughts were introduced.<br>CG: No treatment control		3-month follow up	Humanistic outcomes:<br>Mental health (12 item General Health Questionnaire)
**Cherkin et al. (11)**	Pitfalls of patient education: Limited success of a program for back pain in primary care<br>1996, United States	RCT<br>293 participants<br>(IG (1): 95; IG (2): 102; CG: 97)	Low back pain	IG (1): Usual care plus an educational booklet.<br>IG (2): Usual care plus a 15-minute educational session and an educational booklet 'Back in Action: A Guide to Understanding Your Low Back Pain and Learning What You Can Do About It'. The nurse answered subjects' questions, reviewed the booklets table of contents, set exercise goals, using an exercise log to monitor progress, and emphasized key points of the booklet.<br>CG: Usual care	Registered nurse	Telephone<br>1-3 days after baseline assessment	Clinical outcomes:<br>Symptom relief<br>Humanistic outcomes:<br>Perceived knowledge<br>Functional status<br>Satisfaction with care<br>Participation in exercise
**Clark et al. (12, 13)**	Effects of a tailored lifestyle self-management intervention in	RCT<br>100 participants	T2DM	IG: The key features of the intervention were assessment, patient participation in goal setting, selecting personalized strategies to overcome barriers and follow-up including evaluation and problem solving. Following the principles of MI, a personalized	Psychologist	Telephone<br>1,3,7 weeks	Clinical outcomes:<br>Estimated daily grams of fat<br>Binge eating

	patients with Type 2 diabetes<br><br>2004, UK	(IG: NA; CG: NA)		program was formulated in which management goals for lifestyle change were negotiated; specific intervention strategies to increase self-efficacy and decrease barriers to change were developed. The patient received a copy of the goal setting form and an appointment made for a follow up telephone call in 1 week to monitor progress. Before patients left they received booklets, which were specially prepared for the intervention reinforcing essentials of healthy eating and the importance of increasing physical activity.<br><br>CG: Usual care			severity among obese persons<br><br>Humanistic outcomes:<br><br>Self-care activities over the past 7 days<br>Current physical activity status<br>Number of days and amount of time spent in leisure time<br>Household and work-related physical activity over the past 7 days<br>Barriers to healthy eating and physical activity<br>Confidence in performing physical activity and diet behaviours<br>QOL (SF-12 survey)<br>Self-esteem
**Clarkson et al. (14, 15)**	How to influence patient oral hygiene behaviour effectively<br><br>2010, UK	c-RCT<br><br>87 practices<br><br>478 participants<br><br>(IG: 234; CG: 244)	Oral hygiene	IG: A powered toothbrush and behavioral advice on timing, method and duration of tooth brushing was framed to target oral self-efficacy and action plans to influence oral hygiene behaviour and therefore clinical outcomes. The content and the delivery of the intervention were standardized as a series of steps taking approximately 5 minutes. This included (1) recommendations for brushing twice a day, for 2 minutes, using an electric toothbrush, fluoride toothpaste. (2) Tooth-brushing technique on model of	Dentist	Face-to-face<br><br>At 8 weeks	Clinical outcomes:<br><br>Percentage of surfaces with plaque and showing gingival bleeding on gentle probing

				mouth (3) Dentist assessment of technique and corrects if required (4) Dentist elicited action plan.<br><br>CG: Routine care			Humanistic outcomes:<br><br>Timing, duration and method<br>Oral hygiene self-efficacy
**Doucette et al. (16)**	Community pharmacist-provided extended diabetes care<br><br>2009, United States	RCT<br><br>78 participants<br><br>(IG: 36; CG: 42)	T2DM	IG: Pharmacists role in the intervention involved a 5-step process of care: gathering information from patients and other sources, evaluating the information, formulating a plan, implementing the plan, monitoring the plan, and following up with the patient and physician to ensure optimal outcomes. Pharmacists were instructed to assess clinical parameters such as HbA1c, LDL and blood pressure and use these values to educate patients and recommend drug therapy changes. Pharmacists assessed self-care activities and made recommendations when appropriate. During the first visit, the study protocol recommended that pharmacists take a patient history, create a medication list, assess clinical markers, review medications and self-care behaviours and identify drug therapy problems. Subsequent visits were intended to allow pharmacists to follow up on previous problems, identify new problems, reassess clinical parameters such as blood glucose and blood pressure and discuss self-care activities. After the visits, pharmacists faxed a one-page progress note to patient's physicians describing the content of the visit.<br><br>CG: Usual care	Pharmacist<br><br>General practitioner	Face-to-face<br><br>3-monthly (up to 4 sessions)	Clinical outcomes:<br><br>Changes in HbA1c; LDL; blood pressure<br><br>Humanistic outcomes:<br><br>Self-report of self-care activities
**Dziedzic et al. (17-19)**	Implementing core NICE guidelines for osteoarthritis in primary care with a model consultation (MOSAICS): a cluster randomized controlled trial	c-RCT<br><br>8 practices<br><br>525 participants<br><br>(IG: 234; CG: 288)	OA	IG: Practices delivered the MOAC which consisted of: an enhanced GP consultation to make, give and explain the diagnosis, and provide initial care for older adults presenting with peripheral joint pain; an OA Guidebook offered by the GP to patients to support OA self-management; advice on analgesia; and up to four follow-up practice nurse consultations to guide patients in self-management for OA with advice on	General practitioner<br><br>Nurses	Face-to-face<br><br>4 (at 2 weeks, then three visits in three months)	Clinical outcomes:<br><br>Measures of pain (peripheral joint pain intensity, OMERACT/OARSI responder criteria)

	2018, United Kingdom			weight management if required, general exercise, and physical activity, with goal-setting as appropriate. Briefly, the intervention followed the Whole Systems Informing Self-Management Engagement (WISE) model for guided self-management including provision of patient information (the OA guidebook), care responsive to patient needs, and good access to follow-up care (practice nurse consultations). The timing of the first appointment with the practice nurse was planned for a minimum of 2 weeks after the initial GP consultation. This gave patients time to read the guidebook and try those self-management strategies they felt were suitable. In the first consultation the practice nurse was asked to refer to the guidebook as a resource to answer questions and clarify issues, ascertain the advice from the GP consultation, negotiate and agree appropriate goals, discuss the need for pain relief and opportunities for healthy eating, physical activity and exercise as appropriate. The timing of up to three follow-up visits with the nurse was agreed between the patient and the practice nurse, but was scheduled to be delivered within 3 months following the GP consultation. The follow-up practice nurse consultations were tailored to the patient's individual needs and could focus on, for example, reviewing the self-management plan, demonstrating exercises (Arthritis Research UK Exercises for Arthritis leaflet), giving advice as to how this could be maintained longer-term or making any necessary referrals to the broader multidisciplinary team. The practice nurse consultations were supported by a specifically tailored Case Report Form (available on request) and a nurse toolkit that included advice leaflets to give to patients (content of the toolkit available on request).<br><br>CG: Usual care			Humanistic outcomes:<br><br>SF-12 physical component score (PCS); self-management and patient enablement; self-efficacy; physical activity; Global assessment of change; SF-12 mental health
**Efraimsson et al. (20)**	Effects of COPD self-care management education at a nurse-	RCT	COPD	IG: Patients in the intervention group received education with an emphasis on self-care ability and how to support the individual based on their unique	Nurse	Face-to-face	Clinical outcomes:<br><br>NA

	led primary health care clinic<br>2008, Sweden	52 participants<br>(IG: 26; CG: 26)		requirements and abilities to cope with disease and treatment. The educational visits were based on motivational dialogue, tailored for each patient based on the severity of illness, age, intellectual capacity and lifestyle, with the following main components: (1) Description of the anatomy and physiology of the airways and the effects of COPD (2) Measurement of respiratory function (spirometry) and explanation of the outcome to the patient (3) Optimization of pharmacological treatment and control of inhalation technique (4) Instructions on the coughing technique to prevent infections and exacerbations (5) Motivational dialogue on smoking cessation (6) Instructions on how to deal with acute exacerbations (7) Measurements of oxygen saturation before and after exertion (8) Assessment and instruction of breathing technique and relation (9) Dialogue on physical activity and exercise (10) Dietary counselling (11) Psychosocial counselling and support (12) Counselling on infection prevention (13) Individual treatment plan in collaboration with the patient.<br>CG: Standard care	General Practitioner<br>Dietician, social worker, physiotherapist, OT were at times consulted	4 sessions between 3-5 months	Humanistic outcomes:<br>QOL (St George's respiratory questionnaire)<br>Knowledge about COPD and smoking habits
**Eikelenboom et al. (21, 22)**	Effectiveness of personalized support for self-management in primary care: a cluster randomized controlled trial<br>2016, The Netherlands	c-RCT<br>15 practices<br>644 participants<br>(IG: 296; CG: 348)	Diabetes	IG: The intervention consisted of screening patients with the SeMaS questionnaire, producing a graphic profile with abilities or barriers for self-management. Patients received tailored feedback. Practice nurses were trained in using the profile to enhance self-management of the patient and provide personalized self-management support. The use of individual care plans and self-management interventions was stimulated. SeMas assesses: perceived burden of disease, self-efficacy, locus of control, social support, coping, anxiety and depression. To guide the type of support, it contains items about computer skills, functioning in groups, and willingness to perform self-care. A 1-page graphic profile of the results was provided to support the patient and health professional in counselling on self-management and make the results of SeMas easy to use. This included	Nurse	Not specified	Clinical outcomes:<br>Patient measures for lifestyle factors (exercise, nutrition, smoking)<br>Humanistic outcomes:<br>Level of patient activation (PAM-13)

				education/ psychoeducation on how to cope with barriers, providing information about the condition, lifestyle, self-monitoring and providing an individual care plan.<br><br>CG: Usual care			
**Farmer et al. (23-25)**	Impact of self-monitoring of blood glucose in the management of patients with non-insulin treated diabetes: open parallel group randomized trial<br><br>2007, UK	RCT<br><br>48 practices<br><br>453 participants<br><br>(IG (1): 150; IG (2): 151; CG: 152)	T2DM	IG (1) Less intensive monitoring: continued to use the goal setting and review techniques introduced at the assessment visit. In addition, they were given a blood glucose meter. They were asked to record three values daily on two days during the week (one after fasting and the other two before meals or two hours after meals) and to aim for glucose levels of 4-6mmol/L after fasting and levels of 6-8mmol/L two hours after meals. They were advised by the nurse to consider contacting their doctor if readings were consistently high (>15mmol/L) or low (<4mmol/L). They were not given information about how to interpret their blood glucose readings. Separate diaries were used to record identified goals and activity to record blood glucose results.<br><br>IG (2) More intensive monitoring: continued to use goal setting and review and were also given a blood glucose meter. They were also given training and support in timing, interpreting and using the results of their blood glucose test to enhance motivation and to maintain adherence to diet, physical activity and drug regimens. They were encouraged to experiment with monitoring to explore the effect of specific activities, such as exercise on their blood glucose level and to reflect on abnormal values in an attempt to identify what might have contributed to them. A single diary was used to record goals, activities and blood glucose results.<br><br>CG: Standardized usual care	Nurse	Face-to-face<br><br>1,3,6,9 months	Clinical outcomes:<br><br>HbA1c<br>Blood pressure<br>Weight<br>Total cholesterol level<br>Ratio of total cholesterol to high density lipoprotein cholesterol<br>Body mass index<br><br>Humanistic outcomes:<br><br>NA
**Ferrone et al. (26)**	The impact of integrated disease management in high-	RCT<br><br>168 participants	COPD	IG: Intervention subjects received on-site spirometry, case management, education, and skills training, including self-management education by a certified respiratory educator (CRE) at baseline (1 h), 3 months	Certified respiratory educator	Face-to-face; telephone	Clinical outcomes:<br><br>FEV1; number of exacerbations

	risk COPD patients in primary care<br><br>2019, Canada	(IG: 84; CG: 84)		post-enrollment (45 min), and either a telephone contact or in-person visit at 6 and 9 months (15–30 min). All visits occurred in the primary care practice where the individual normally received care. The CREs involved were all regulated healthcare professionals whose scope of practice included patient counseling and who have successfully completed a Canadian Network for Respiratory Care approved respiratory educator program. The CREs that were COPD certified for this project were experienced asthma educators who provided services in an established primary care asthma program. During patient encounters, CREs were supported by a scalable electronic point of service system (POSS) developed for the project that guided them through the standardized evidence-based interventions and recorded all care elements delivered. The IDM intervention identified patient-specific goals and emphasized shared decision making. The specific elements of IDM are categorized under case management, education, and skills training. The final management plan for each in-person visit was confirmed by the primary care physician during a 5–7 min encounter immediately following the CRE evaluation.<br><br>CG: Usual care		At 3 months (45 min), and either a telephone contact or in-person visit at 6 and 9 months (15–30 min)	Humanistic outcomes: COPD specific QOL; COPD knowledge
**Fortin et al. (27, 28)**	Integration of chronic disease prevention and management services into primary care: a pragmatic randomized controlled trial (PR1MaC)<br><br>2016, Canada	RCT<br><br>305 participants<br><br>(IG: 144; CG: 161)	Diabetes, cardiovascular disease, COPD, asthma, tobacco smoking, obesity, hyperlipidemia, prediabetes, sedentary lifestyle or any combination of these	IG: The principles guiding the intervention were based on self-management support and health education, a patient-centered approach, motivational approach and interprofessional collaboration. For each patient, the intervention started with a preliminary clinical evaluation by a trained nurse. then designed an individualized intervention plan in collaboration with the patient that could include encounters with 1 or more CDPM professionals in the following disciplines: nursing, physical activity, nutrition, respiratory therapy and smoking cessation therapy. The intervention plan was based on the patient's objectives as identified at the first encounter but could be further adapted by any professional in each	Nurse	Face-to-face<br><br>(2 sessions, interval not specified)	Clinical outcomes:<br><br>NA<br><br>Humanistic outcomes:<br><br>Self-management; self-efficacy, health-related quality of life, psychological distress; lifestyle factors

				discipline involved. Interventions were done by CDPM professionals who were recruited and trained by the research team and travelled from 1 organization to the other to deliver the services.<br>CG: Delayed treatment control			
**Freund et al. (29)**	The effect of preventive consultations on young adults with psychosocial problems: a randomized trial<br>2012, Denmark	RCT<br>495 participants<br>(IG: 240; CG: 255)	Psychosocial problems	IG: GPs were recommended to skim the BQ and then start by asking the following questions 'How was it like to complete the questionnaire?' and 'What do you prefer to discuss?' Completing the BQ was supposed to facilitate insight into the relationship between social life, health, lifestyle, own reaction on stressors and resources and barriers for gaining control and changing behaviour. At the end of the consultation, the GP and the patient together made a written report of their general impression of the consultation, general health, resources, network and lifestyle. The patient chose one or two goals. Goals setting, time schedule and specific resources and barriers for reaching the goals were discussed and shortly described in the three-page report. Needs for other interventions were discussed.<br>CG: Usual care	General practitioner	Telephone<br>At 3 months	Clinical outcomes:<br>Blood pressure<br>Body mass index<br>Blood glucose level<br>Cholesterol<br>Humanistic outcomes:<br>Change in HRQOL after 1 year (SF-12 survey)
**Friedberg et al. (30, 31)**	Chronic fatigue self-management in primary care: a randomized trial<br>2013, United States	RCT<br>111 participants<br>(IG: 37; CG (1): 38; CG (2): 36)	Unexplained chronic fatigue & chronic fatigue syndrome	IG: This two-session nurse conducted individual self-management intervention was based on a modified version of an efficacious 12-session cognitive-behavioral treatment program for chronic fatigue syndrome and a self-help book. A 61-page self-management booklet provided to these participants contained material discussed and assigned in therapy sessions for the three-month self-management period. Session 1: This session educated the participant about (1) diagnosis and possible causal factors in chronic fatigue (2) stress factors and behaviours that play a role in disturbed sleep patterns, post-exertion symptoms, and push-crash activity cycles. Persistent fatigue was explained as a symptom associated with doing too much or too little. Optimal self-management was intended to achieve a	Nurse	Face-to-face<br>At 2 weeks	Clinical outcomes:<br>Fatigue impact on functioning (Fatigue Severity Scale)<br>Humanistic outcomes:<br>NA

				healthy balance between mental and physical exertion and periods of rest. Assignments included the self-management booklet and a daily web diary to identify baseline activities, symptoms, and stress levels. Session 2: Scheduled two weeks after session 1, this session identified unhelpful behaviours and beliefs about the illness followed by development of more useful cognitive and behavioral coping strategies. With information gathered from the week 1 web diary, the scheduling of home-based activities, rest/sleep assignments, and cognitive coping skills was individualized for each participant. Walking, if included, was intended as a voluntary leisure activity, rather than a fitness regimen. For instance, a relatively low functioning individual might be assigned a regular sleep /wake schedule and gradual low effort walking to increase tolerance of physical activity. A higher functioning participant might respond more favorably to pacing of activity and low effort pleasant activities. The final topic was post intervention planning for maintenance of new self-management skills which included recognizing and managing early symptoms of setbacks before they affected functioning.<br><br>CG (1): Attention control. To control for therapist attention, homework assignments, and other non-specific effects, a two-session attention control condition was incorporated into this study. This condition included (1) in-session emotional support and (2) home-based self-monitoring of symptoms, affect and stress as recorded in web diaries. The two face-to-face sessions in this condition were separated by two weeks.<br><br>CG (2): Usual care			
**Gabbay et al. (32)**	Nurse case management improves blood pressure, emotional distress and diabetes	RCT<br><br>332 participants	Diabetes	IG: The nurse implemented specific diabetes management algorithms under the supervision of the patient's primary care physician. Goals were based on ADA recommendations: BP < 130/80mmHg, LDL < 100, HbA1c < 7%, quarterly HbA1C measurement, bi-	Registered Nurse	Face-to-face<br><br>4-monthly	Clinical outcomes:<br><br>Changes in blood pressure, HbA1c, and lipids

	complication screening<br><br>2006, United States	(IG: 150; CG: 182)		annual lipid measurement, yearly ophthalmological and monofilament exam, micro albumin/ creatinine ratio, flu vaccine, appropriate Pneumovax immunization, certified diabetes nurse educator and dietician visits. The nurse case manager used behavioral goal setting, established individualized care plan, provided patient self-management education and surveillance of patients, including phone calls to patients, referred patients to a diabetes nurse educator or a dietician where appropriate, ordered protocol driver laboratory tests, tracked the outcomes using the computerized data registry and made therapeutic recommendations based on ADA diabetes guidelines with approval from the primary care provider. An initial 45 to 60-minute baseline visit provided an opportunity for patient assessment and development of an individualized care plan to focus on specific shortcomings of clinical parameters and to establish patient centered behavioral goals.<br><br>CG: Usual care			Complication screening process measures<br><br>Humanistic outcomes:<br><br>Diabetes-related distress
**Gabbay et al. (33)**	Diabetes nurse case management and motivational interviewing for change (DYNAMIC): Results of a 2-year randomized controlled pragmatic trial<br><br>2013, United States	RCT<br><br>545 participants<br><br>(IG: 232; CG: 313)	T2DM	IG: The visits typically included a review of patients' clinical laboratory test results, health-related lifestyle behaviour relevant to managing T2DM and medication adherence. The nurses also checked whether the patient was due for complications screening and reminded them of follow-up specialist visits when they were due. Referrals to a certified diabetes nurse educator or a dietician were done where appropriate. Finally, nurses prompted the physicians for medication titrations when necessary. These were done via e-mail, in person, or phone call, depending on the PCP's preference.<br><br>CG: Routine care	Nurse	Face-to-face<br><br>2,6,12 weeks, 6,12 months, and at least every 6 months<br><br>Telephone; email<br>Variable	Clinical outcomes:<br><br>Blood pressure<br>HbA1c<br>Lipids<br>Depression symptom scores (Centre for Epidemiologic Studies Depression (CES-D) scale)<br><br>Humanistic outcomes:<br><br>Diabetes-related distress (Problem Areas in Diabetes scale)<br>Treatment

							satisfaction (Diabetes Treatment Satisfaction Questionnaire)<br>Self-care activities (Summary of Diabetes Self-Care Activities)<br>QOL (Audit of Diabetes Dependent Quality of Life)
**Goudswaard et al. (34)**	Long-term effects of self-management education for patients with Type 2 diabetes taking maximal oral hypoglycemic therapy: a randomized trial in primary care<br><br>2004, The Netherlands	RCT<br><br>54 participants<br><br>(IG: 25; CG: 29)	T2DM	IG: The educational program focused on: general information on diabetes; reinforcing compliance with actual medication; importance of physical exercise and losing body weight; and nutritional advice. All patients were also taught how to control their blood glucose at home on a regular basis for which they were given a blood glucose meter and strips.<br><br>CG: Usual care	Nurse	Face-to-face<br><br>3-6 weekly (total 6 sessions)	Clinical outcomes:<br><br>HbA1c<br>Weight<br><br>Humanistic outcomes:<br><br>NA
**Grilo et al. (35)**	Self-help for binge eating disorder in primary care: a randomized controlled trial with ethnically and racially diverse obese patients<br><br>2013, United States	RCT<br><br>48 participants<br><br>(IG: 24; CG 24)	Binge-eating disorder	IG: Self-help cognitive behaviour therapy (CBT) was provided in addition to usual care and involved being given Overcoming Binge Eating a self-help program (self-help manual) which follows the professional CBT program and is considered to be the treatment of choice for Binge Eating Disorder. The self-help manual has 3 stages. The first stage consisted of: presentation of the CBT model including the structure, goals, and methods; education regarding binge eating, dieting, and health; introduction of self-monitoring techniques; and introduction of graded behavioral techniques for establishing normalized eating patterns. The second stage consisted of maintaining the normalized eating and self-monitoring procedures and integrates cognitive restructuring procedures and	General practitioner	Face-to-face<br><br>Monthly (total 4 sessions)	Clinical outcomes:<br><br>Binge eating rates of “remission” during the previous 28 days<br>Depression levels (BDI score)<br>Body mass index<br><br>Humanistic outcomes:<br><br>Continuous measures of eating disorder

				the development of coping skills for triggers of maladaptive eating. The third stage focused on consolidating progress, maintenance of changes, and relapse prevention methods. The manual provided guidance as to when to move on to the next step of the program.<br><br>CG: Usual care			psychopathology (EDE global score, EDE-Q global score)
**Heitkemper et al. (36)**	Self-management for women with irritable bowel syndrome<br><br>2004, United States	RCT<br><br>132 participants<br><br>(IG (1): 40; IG (2): 48; CG: 44)	Irritable bowel syndrome	IG (1): Comprehensive treatment. Session 1: Introduction and review baseline assessment, Session 2: Physiologic arousal and abdominal breathing, Session 3: Diet and automatic thoughts, Session 4: Automatic thoughts and active progressive muscle relaxation, Session 5: Cognitive restructuring and mini-relaxation, Session 6: Targeted problem-solving skills and passive relaxation, Session 7: Consolidate strategies and review goals, plan, and obstacles, Session 8: Maintenance and termination. Individual sessions included a review of homework assignments from the previous session, new strategies were presented, and homework based on the content of the session was assigned. Adherence strategies were integrated throughout the sessions (e.g., breaking tasks into small pieces, beginning with assignments that allow participants to succeed reinforcement, rehearsal of assignments). Assignments included symptom monitoring and other homework specific to the intervention. At the last session, an individualized management plan was developed. The intervention had the following 4 components. Education and reassurance. In addition to defining IBS and providing reassurance that IBS is not life threatening, this content also included a discussion of signs and symptoms for which it is important to consult a health care provider. Dietary counselling. The participants were first instructed on healthy eating strategies; for example, participants were encouraged to eat small frequent meals/ snacks and slowly increase their fiber intake to 20–25 g/day. In addition, participants were taught to recognize foods that were associated with	Nurse practitioner	IG (1)<br><br>Face-to-face<br><br>Weekly for 8 weeks<br><br>IG (2)<br><br>Face-to-face<br><br>At 8 weeks	Clinical outcomes:<br><br>Improvements in symptoms<br><br>Humanistic outcomes:<br><br>Psychological distress<br>HRQOL<br>Indicators of stress-related hormones

				their symptoms (e.g., coffee, fatty foods, raw vegetables) as well as situations when select foods were not tolerated (e.g., a time of high work stress). Homework included keeping a food diary to identify when they ate, what they ate, and what was happening in their environment. Relaxation training. Abdominal breathing, progressive muscle relaxation, and mini-relaxation were taught. Homework included abdominal breathing at least 3 times a day (e.g., before each meal), use of the relaxation audiotape 3 times a week, and daily mini-relaxation with tension as a cue. Cognitive-behavioral strategies. Specific cognitive-behavioral strategies were selected on the basis of individualized assessment. These included anger management, cognitive restructuring, assertiveness and social skills training, and social support. Homework included writing down automatic thoughts and identifying and using alternative thoughts.<br><br>IG (2): The Brief program attempted to cover the same material as the Comprehensive program, but it was condensed into one 90-minute session. Each participant was given the same workbook and relaxation tape; the nurse discussed how dietary changes, relaxation exercises, and cognitive strategies could be integrated into lifestyle.<br><br>CG: Usual care			
**Hill et al. (37)**	Disease-specific education in the primary care setting increases the knowledge of people with chronic obstructive pulmonary disease: A randomized controlled trial<br><br>2010, Canada	RCT<br><br>93 participants<br><br>(IG: 50; CG: 43)	COPD	IG: Individuals allocated to the intervention attended two one-to-one education sessions, the first of which was scheduled one month following randomization and the second one-month later. A certified COPD educator performed both 60-minute sessions as face-to-face discussion at the primary care practice. The educational content was standardized. The sessions focused on enhancing self-efficacy in areas likely to be important to individuals recently diagnosed with COPD. Specifically, the following topics were addressed (1) normal lung function (2) how COPD affects the lungs (3) symptoms and what makes them	Certified COPD educator	Face-to-face<br><br>At 4 weeks	Clinical outcomes:<br><br>NA<br><br>Humanistic outcomes:<br><br>COPD knowledge (Bristol COPD Knowledge Questionnaire)

				worse (4) strategies for smoking cessation (5) respiratory medications (6) symptoms of an acute exacerbation (7) the role of regular exercise. A written teaching manual adapted from the “Living Well with COPD” program accompanied the oral information. Patients were given this after their second education session.<br><br>CG: Usual care			
**Hoffmann et al. (38)**	Pharmaceutical care for migraine and headache patients: a community-based, randomized intervention<br><br>2008, Germany	c-RCT<br><br>83 practices<br><br>410 participants<br><br>(IG: 201; CG: 209)	Migraine/ Headache	IG: The intervention included individual counselling with a defined extended time frame, usually provided in designated rooms. All specific steps of the intervention were documented by the pharmacist in semi-standardized forms including the number of consultations, demographic variables of the patient, medical history, nutrition, allergies, headache and migraine characteristics, past and present medication. Together with the patient, the intervention pharmacist prioritized problems, defined individual goals, and devised a plan to work toward them.<br><br>CG: Usual care	Pharmacist	Face-to-face<br><br>Up to 6 sessions<br><br>Telephone<br><br>At 4 months	Clinical outcomes:<br><br>Number of days with headache<br>Number and severity of headaches<br><br>Humanistic outcomes:<br><br>Self-efficacy<br>HRQOL
**Huang et al. (39, 40)**	Prospective randomized controlled trial to evaluate effectiveness of registered dietician-led diabetes management on glycemic and diet control in a primary care setting in Taiwan<br><br>2010, Taiwan	RCT<br><br>154 participants<br><br>(IG: 75; CG: 79)	T2DM	IG: Patients in the intervention group, in addition to receiving usual care, received ongoing instruction on the self-monitoring of glucose, medications, exercise, foot care and complication management. Patients in the intervention group were also provided individualized nutrition counselling and dietary plans to reinforce the concepts on controlling portion sizes of foods every 3 months. The registered dietician assessed patients understanding and practice of dietary plans and self-care skills and reinforced important knowledge throughout the study period. The physicians consulted with the registered dieticians based on medicines prescribed or patient’s self-care related to adjustment of meal times and amount of food. During each intervention visit, the dietician obtained daily nutrient intake by asking patients to recall the foods consumer for the previous	Dietician	Face-to-face<br><br>3-monthly for 12 months (total 4 sessions)	Clinical outcomes:<br><br>Anthropometric measurements<br>Clinical lab measurements after an 8 to 12 hour fast<br>Dietary habits<br><br>Humanistic outcomes:<br><br>NA

				24-hour period. Each patient received dietary education recommended by the ADA. An individualized diet plan was created to maintain intake of protein, fat, and carbohydrate energy to ~15-20, 25-30 and 50-60%.<br><br>CG: Routine care			
**Ismail et al. (41)**	Usage of glucometer is associated with improved glycemic control in type 2 diabetes mellitus patients in Malaysian public primary care clinics: an open-label, randomized controlled trial<br><br>2013, Malaysia	RCT<br><br>105 participants<br><br>(IG: 58; CG: 47)	T2DM	IG: All patients received similar health education, as recommended in the Malaysian Clinical Practice Guidelines on the management of diabetes mellitus, which highlighted the need for strict glycemic control, diet control, blood glucose monitoring, and knowledge on how to adjust the dose of oral hypoglycemic agents (OHA) or insulin, as well as treatment of hypoglycemia. In addition, participants were offered two-day classes that included practical demonstrations of self-monitoring blood glucose, during which the usage of the glucometer was explained. Patients were supplied a glucometer with reagent test strips at no charge, after they demonstrated the skill needed to use the device. Patients were advised to monitor their blood glucose levels (either during fasting, two hours after breakfast, or two hours after meals) and to keep a record in their logbooks. If the test result was found to be above the set target value (i.e. fasting blood glucose > 6.0 mmol/L; postprandial blood glucose > 7.8 mmol/L), the patient was advised to adjust the dose of OHA/insulin accordingly and recheck the blood glucose level of that particular time (either during fasting or postprandial), after four to five days.<br><br>CG: NA	Nurse	Face-to-face<br><br>2-monthly (total 3 sessions)	Clinical outcomes:<br><br>Fasting blood glucose or two-hour postprandial blood glucose<br>HbA1c<br>Fasting cholesterol<br>Triglycerides<br>Serum creatinine<br>Weight<br>Blood pressure<br><br>Humanistic outcomes:<br><br>NA
**Jaipakdee et al. (42)**	Effectiveness of a self-management support program for Thais with type 2 diabetes: Evaluation according to the RE-AIM framework	c-RCT<br><br>10 practices<br><br>403 participants	T2DM	IG: The diabetes self-management support program was adapted from three strategies, including diabetes education, behaviour change support, and emotional support program comprised of two components: (I) diabetic educational section to help patients understand the disease process; and (II) proper skill learning to manage their condition and change their	Nurse	Face-to-face<br><br>3,6 months	Clinical outcomes:<br><br>HbA1c<br><br>Humanistic outcomes:<br><br>NA

	2015, Thailand	(IG: 203; CG: 200)		lifestyle. In Component I, a CAI was developed for use in the educational sessions. The CAI facilitated the DSMS delivery lesson by lesson according to the pre-set steps, while lesson repetition is also possible. Its video component included lessons on diabetes and pre- and post-tests (10-questions with three choice answers of each lesson). The lessons consisted of: (1) knowledge of diabetes; (2) foods for diabetes; (3) physical activity; (4) foot care; (5) medication used to control diabetes; (6) reducing complications and stress management; (7) self-monitoring of clinical indicators and goals of diabetes control. The lessons were designed in various forms such as stories, graphics, animated images, interviews, and demonstrations to stimulate learners' interest and enjoyment. In Component II, the step-by-step approach for behaviour change and psychological support was in accordance with the 5C intervention and consisted of: (1) constructing a problem definition; (2) collaborative goal setting; (3) collaborative problem solving; (4) contracting for change; (5) continuing support. The nurse supporters performed as facilitators to help participants define their problem in a potentially useful way, set goals, identify barriers, and solve problems in achieving those goals. Participants were followed-up for their behaviour changes that were engaged in the previous session, such as dietary habits, exercise, foot care, medication, and blood glucose monitoring.<br>CG: Usual care			
**Kennedy et al. (43, 44)**	Implementation of self-management support for long term conditions in routine primary care settings: cluster randomized controlled trial<br>2013, United Kingdom	c-RCT<br>41 practices<br>5599 participants<br>(IG: 2295; CG: 3304)	Diabetes, COPD, IBS	IG: The intervention (whole system informing self-management engagement, WISE) is based on accumulated evidence from multiple randomized controlled trials and an ongoing program of work grounded in primary care.7 28-31 The core aim was to take several components found to be effective in these previous studies and to deliver them as a comprehensive package under naturalistic conditions and using routine care providers to maximize real	General practitioners<br>Practice nurses	Not specified	Clinical outcomes:<br>NA<br>Humanistic outcomes:<br>Shared decision making; self-efficacy, generic health related

				world applicability. The intervention was designed to be feasible to implement widely in primary care, which put practical limitations on the intensity of the intervention. Training (developed and piloted with two non-trial practices) was delivered in each practice over two sessions, which we estimated through informed feedback was the maximum feasible in UK primary care using current educational structures. Session 1 involved all practice staff (doctors, nurses, technicians, and administration staff) and session 2 focused on clinical staff. Fidelity checks and reinforcement sessions with trainers were scheduled after training. Two facilitators employed by the primary care trust delivered the training and also provided access to self-management support activities and resources in the primary care trust. The practices were provided with resources to support self-management, including a tool to assess patient support needs and priorities (PRISMS). In session 1, practices worked on ways to embed self-management tools in their systems; in session 2, clinicians practiced ways to use core self-management skills in consultations and ensure patients received, or were directed to, appropriate resources. Assessment of patient need was linked to appropriate support, including self help guidebooks based on published development methods, access to relevant community groups and programs.<br><br>CG: Wait list comparator			quality of life; general health; social or role limitations; energy and vitality; psychological wellbeing; self-care activity; and enablement
**McGeoch et al. (45)**	Self-management plans in the primary care of patients with chronic obstructive pulmonary disease<br><br>2006, New Zealand	c-RCT<br><br>159 participants<br><br>(IG: 86; CG: 73)	COPD	IG: The intervention group received usual care and education on the use of a self-management plan. The plan and structured education included methods of early recognition of exacerbations and a range of appropriate self-initiated interventions including antibiotics and short course of oral corticosteroids. In addition, patients were instructed to make early contact with their general practice during exacerbations. Standardized self-management plan education was delivered in an individual session of 1-	Practice nurse or respiratory educator<br><br>General practitioner	Face-to-face<br><br>1 session at 12 months<br><br>Telephone<br><br>3, 6, 9 months	Clinical outcomes:<br><br>Symptoms (St Georges Respiratory Questionnaire)<br>Courses of antibiotics<br>Courses of oral steroids

				hour duration from a practice nurse or respiratory educator in association with their general practitioner. The sessions covered the major points of the COPD self-management plan and the use of previously validated sputum color charts.<br><br>CG: Usual care			Humanistic outcomes:<br><br>Self-report anxiety and depression (Hospital Anxiety and Depression Scale)<br>Self-management (COPD Self-Management Interview)
**McLean et al. (46)**	The BC Community Pharmacy Asthma Study: A study of clinical, economic and holistic outcomes influenced by an asthma care protocol provided by specially trained community pharmacists in British Columbia<br><br>Canada, 2003	c-RCT<br><br>18 pharmacies<br><br>631 participants<br><br>(IG: 191; CG (1): 214; CG (2): 226)	Asthma	IG: Intervention (EC) involved soliciting all of the UC information plus the teaching of asthma self-management as outlined in the HOP Asthma Care Module. This involved instruction on the basic concepts of the disease, the medications being used and trigger identification and avoidance, as well as the development of the asthma action plan. In addition, the use of a peak flow meter was taught, calendars/diaries were provided and the patient asked to record PEFRs regularly for the course of the study period. Also, spacer devices were used by all patients requiring them for better utilization of their medications. Care in the EC group involved appointments of approximately one hour in length with a pharmacist in a private counselling area every two to three weeks for at least three appointments, and then follow-up appointments at least every three months for the remainder of the study. Patients could request additional appointments or could see the pharmacist intermittently for short sessions without an appointment. An initial assessment of 'readiness for change' was completed using the Transtheoretical Model of Change and patients were reassessed at each appointment. Education did not begin until the patient was in 'contemplation' stage, and the new strategies were not begun until the patient was in 'preparation' stage. EC patients received	Pharmacist	Face-to-face<br><br>(Every 2-3 weeks, for at least three appointments and then follow-up appointments at least every three months)	Clinical outcomes:<br><br>Peak expiratory flow rates; symptom scores (dyspnea, cough, wheeze, chest tightness, phlegm production and nasal symptoms)<br><br>Humanistic outcomes:<br><br>Patient's knowledge; QoL

				'pharmaceutical care'; thus, EC may be summarized as:<br>• pharmacist assesses readiness to change and adjusts initiation date<br>• pharmacist provides education on disease, helps identify triggers and works with patient to develop action plan<br>• patient participates in all decisions<br>• patient monitors own therapy (PEFRs, using calendar/diary)<br>• pharmacist takes responsibility for outcomes<br>• pharmacist promotes evidence-based care<br>• pharmacist-patient interaction based on appointment and occurs in private consultation area<br>• physician informed or consulted regarding all results and interventions<br>CG: Usual care			
**Mehuys et al. (47)**	Effectiveness of pharmacist intervention for asthma control improvement<br>2008, Belgium	RCT<br>201 participants<br>(IG: 107; CG: 94)	Asthma	IG: Session 1: Participants received personal education from the pharmacist about the following topics (1) Correct use of the inhaler device (2) Understanding asthma, symptoms, triggers, early warnings (3) Understanding asthma medication, difference between controller and reliever, adherence to controller medication (4) Smoking cessation if required. Session 2 & 3: at 1 month and 3-month follow up: Pharmacist advice based on ACT score of the patient. If ACT score < 15 - immediate referral to GP or respiratory specialist. If ACT 15-19: review inhalation technique and check controller medication adherence. If ACT ≥ 20: no specific advice needed, inform patient asthma is well controlled.<br>CG: Usual care	Pharmacist	Face-to-face<br>1, 3 months	Clinical outcomes:<br>Level of asthma control<br>Peak expiratory flow<br>Rescue medication use<br>Night-time awakenings due to asthma<br>Inhalation technique<br>Adherence to controller medication<br>Severe exacerbations

							Humanistic outcomes:<br>QOL<br>Knowledge on asthma and smoking behaviour
**Mehuys et al. (48)**	Effectiveness of a community pharmacist intervention in diabetes care: a randomized controlled trial<br>2011, Belgium	c-RCT<br>66 practices<br>288 participants<br>(IG: 153; CG: 135)	T2DM	IG: Patients in the intervention group received a protocol-defined intervention at the start of the study and at each prescription-refill visit (for hypoglycemic medication) during the course of the study. The intervention consisted of several elements: (1) Education about T2DM and its complications; (2) Education about the correct use of oral hypoglycemic agents (timing in relation to food); (3) Facilitation of medication adherence (by counselling); (4) Healthy lifestyle education (diet, physical exercise and smoking cessation); and (5) Reminders about annual eye and foot examinations.<br>CG: Usual care	Pharmacist	Face-to-face<br>At each prescription refill visit	Clinical outcomes:<br>HbA1c<br>Humanistic outcomes:<br>NA
**Meland et al. (49, 50)**	Effectiveness of two preventive interventions for coronary heart disease in primary care<br>1997, Norway	c-RCT<br>22 participants<br>110 patients<br>(IG: 58; CG: 52)	Coronary heart disease	IG: Participants were provided with self-help material based on cognitive behaviour change. A behavioral intervention was chosen by the patient at each consultation including cholesterol reduction, weight reduction, salt reduced diets, leisure time exercise, smoking cessation and stress management. The cholesterol lowering self-help brochure invited patients to self-monitor their everyday diet and make a contract on dietary changes. The smoking cessation program employed self-monitoring, gradually breaking smoking habits and addiction, and motivational behaviour change. Patients were also offered a stress-coping audiotape containing general relaxation and self-cognitive instructions.<br>CG: Conventional care	General practitioner	Face-to-face<br>Three-monthly	Clinical outcomes:<br>Blood pressure<br>Weight<br>Resting pulse<br>Total serum cholesterol<br>Triglycerides<br>Humanistic outcomes:<br>Self-efficacy in diet, physical exercise and smoking<br>Smoking status

**Mitchell et al. (51, 52)**	A self-management program for COPD: a randomized controlled trial<br><br>2014, UK	RCT<br><br>184 participants<br><br>(IG: 89; CG: 95)	COPD	IG: In addition to usual care, intervention participants received the self-management program structured around the SPACE FOR COPD manual (a 176-page workbook that individuals can follow independently at home). The manual, divided into four sections, contains educational material and a home exercise program. Acquisition of skills is promoted through goal-setting strategies, coping planning and case studies. It incorporates an exercise regime that consists of a daily walking program, and resistance training of the upper and lower limbs using free weights three times per week. The manual advises on training progression and includes an action plan for exacerbation management. Participants randomized to SPACE FOR COPD were introduced to the program by a physiotherapist during a 30–45-min consultation. MI techniques were used to underpin the consultation in order to explore the patients' readiness to change and to enhance motivation for adopting new lifestyle behaviours. Participants' needs were discussed, and goal-setting strategies were introduced. Participants were advised how to use the manual at home and the exercise regime was described by the physiotherapist in detail. It was anticipated that participants would work through the manual in approximately 6 weeks; however, participants were advised the manual was theirs to keep, as it could be used as a resource for the future, and that the lifestyle changes it suggested should be lifelong.<br><br>CG: Usual care	Physiotherapist	Telephone<br><br>2,4 weeks	Clinical outcomes:<br><br>Symptom burden (Chronic Respiratory Questionnaire)<br><br>Humanistic outcomes:<br><br>Shuttle walking tests Disease knowledge Anxiety/ depression Self-efficacy Smoking status
**Morgan et al. (53, 54)**	The TrueBlue model of collaborative care using practice nurses as case managers for depression alongside diabetes or heart disease: a randomized trial	c-RCT<br><br>11 practices<br><br>317 participants<br><br>(IG: 170; CG: 147)	Depression<br><br>Diabetes<br><br>Heart Disease	IG: The nurse and patient identified possible barriers to achieving their goals and discussed enabling methods that may overcome these barriers. The nurse supplied educational material to assist patients in understanding their condition and meeting their goals. This information was then added to the GP management plan. The GP completed the consultation with the patient, providing the patient	Nurse<br><br>General Practitioner	Face-to-face<br><br>3-monthly (total 4 sessions)	Clinical outcomes:<br><br>Five-point reduction in depression scores for patients with moderate-to-severe depression Improvement in

	2013, Australia			with a copy of the completed management plan to follow at home.<br><br>CG: Wait list control			physiological measures<br><br>Humanistic outcomes:<br><br>NA
**Moss-Morris et al. (55, 56)**	A randomized controlled trial of a cognitive behavioral therapy-based self-management intervention for irritable bowel syndrome in primary care<br><br>2010, UK	RCT<br><br>64 participants<br><br>(IG: 31; CG: 33)	Irritable bowel syndrome	IG: Participants received an IBS fact sheet after their diagnosis was confirmed. In addition, they were provided with a comprehensive self-management manual that included the provision of information, real life examples, and weekly homework sheets that they were encouraged to complete. The program was divided into seven chapters, one to be completed each week over a 7 to 8-week period. Participants received a 1-hour face-to-face session with a health psychologist at the beginning of the program. Participants received two 1-hour therapy sessions by telephone schedule midway and towards the end of the program. They were intended to give the patient an opportunity to go through any queries they might have, to clarify the appropriateness of the goals set, and to work through some of the more complex aspects of the program such as managing unhelpful thoughts. Chapter 1: IBS explained; Treatment rationale, which includes the following explanations: Illustrative physiology of the digestive system together with the functional changes that occur in the gut as a result of IBS; How the autonomic nervous system ('fight-or-flight' stress system) may interact with the enteric nervous system; The interaction between thoughts, feeling and behaviours and how these can impact on stress levels and gut symptoms. Chapter 2: Assessing symptoms and self-monitoring; Participants begin to make the link between their own symptoms, thoughts and behaviours. The pitfalls of becoming overly symptom focused are discussed; Participants keep daily diaries of the severity and experience of IBS symptoms in conjunction with stress levels experienced and eating routines/behaviours. Chapter 3: Managing IBS symptoms; Behavioral	Psychologist	Telephone<br><br>2 sessions in 8 weeks	Clinical outcomes:<br><br>Symptom severity (Irritable Bowel Syndrome Severity Scoring System)<br>Self-report anxiety and depression (Hospital Anxiety and Depression Scale)<br>Symptom relief (Subjects Global Assessment of Relief)<br><br>Humanistic outcomes:<br>Work and Social Adjustment

				management of the symptoms of diarrhoea and constipation and common myths in this area are discussed. Goal setting is explained; The importance of healthy eating and exercise regimes is covered, and participants are encouraged to set goals for managing symptoms, exercise and diet. Goal setting, monitoring and evaluation continue weekly through the program. Chapter 4: Managing unhelpful thoughts; The concept of negative automatic thoughts and how these can impact on IBS symptoms is introduced; Participants are asked to keep a daily thought record of unhelpful thoughts and to try and come up with alternative thoughts. Chapter 5: Personal expectations and activity patterns; The concept of perfectionism and unhelpful personal expectations is introduced. How these may lead to an all-or-nothing style of activity is addressed; Participants are asked to keep daily thought records of unhelpful thoughts related to personal expectations and patterns of over activity. Chapter 6: Relaxation and stress management; Basic stress management and sleep hygiene are discussed. A relaxation CD is provided, and participants are encouraged to set goals for relaxation and improving sleep over a 15-day period. Chapter 7: Managing flare-ups and the future; The probability of flare-ups is discussed, and patients are encouraged to develop achievable, long-term goals and to continue to use the skills they have learnt throughout the manual to manage flare-ups and ongoing symptoms.<br><br>CG: Treatment as usual			
**Murphy et al. (57-59)**	Effect of tailored practice and patient care plans on secondary prevention of heart disease in general practice: cluster randomized controlled trial	c-RCT<br><br>48 practices<br><br>903 participants<br><br>(IG: 444, CG: 459)	Coronary heart disease	IG: A multifaceted intervention comprising care plans for both the practices and the patients. An action plan for each patient was agreed with the practice and regularly reviewed by the study research nurse and practice. The study nurse maintained regular contact with the practices through a 2-page study newsletter provided to practices every four months. The patient and practitioner together identified areas of management that could be improved, and the patient	General practitioners<br><br>Practice Nurses	Face-to-face<br><br>4-monthly<br><br>Telephone<br><br>At 2 weeks	Clinical outcomes:<br><br>Blood pressure<br>Total cholesterol<br><br>Humanistic outcomes:<br><br>Physical and mental health

	2009, UK			was invited to prioritize one particular aspect of his or her lifestyle for change. Possible ways of achieving targets reflecting optimal management were identified and action plans individualized so that small, realistic goals for change were agreed. Booklet containing information on all the key risk factors for coronary heart disease was used by practitioners in discussions on initial target setting and then given to the patients. Six sections of the book include medications, smoking, exercise, healthy eating, stress and community support.<br><br>CG: Usual care			status (SF-12 survey)
**Olry de Labry Lima et al. (60)**	Effectiveness of an intervention to improve diabetes self-management on clinical outcomes in patients with low educational level<br><br>2017, Spain	c-RCT<br><br>9 general practitioners<br><br>184 participants<br><br>(IG: 90; CG: 94)	T2DM	IG (1): Face-to-face intervention carried out by GPs during the clinic visit and consisted of seven visits, one every three months. Each session consisted of completing a diabetes care record sheet (DCRS) together with the patient. The DCRS consisted of two parts: Five questions on self-care activities in the last three months and a graph with previously measured HbA1c levels. This information was completed at each session, resulting in a graph showing the evolution of glycemic control related to self-care activities. The DCRS was explained to patients, emphasizing the relationship between self-care and glycemic control. At the end of the session, patients were given a copy of the DCRS and suggested to show it and discuss it with their relatives.<br><br>IG (2): Face-to-face intervention plus telephone reinforcement. In this group patients received the above described intervention plus a telephone reinforcement. It consisted of five telephone calls lasting about 10 minutes each, to provide advice on carrying out physical exercise and eating a balanced diet and to encourage the use of health services related to diabetes control. Telephone follow-up reinforced T2DM self-management and motivational interviewing techniques.<br><br>CG: Standard care	General practitioner	IG (1):<br><br>Face-to-face<br><br>3-monthly (total 7 sessions)<br><br>IG (2):<br><br>Face-to-face<br><br>3-monthly (total 7 sessions)<br><br>Telephone<br><br>Variable (total 5 phone calls)	Clinical outcomes:<br><br>HbA1c<br>Blood pressure (systolic and diastolic)<br>Lipids (triglycerides, high density lipoprotein and low-density lipoprotein)<br>Body mass index<br>Waist circumference<br><br>Humanistic outcomes:<br><br>NA

**Partapsingh et al. (61)**	Applying the Stages of Change model to Type 2 diabetes care in Trinidad: A randomized trial<br>2011, Trinidad	RCT<br>122 participants<br>(IG: 61; CG: 61)	T2DM	IG: The intervention was 'stage-specific' and personalized. Care was delivered to Type 2 diabetics specific to the patient's current stage of change (SOC) and specific to the patient as a whole. These formats divided each consultation into sections specific for the named SOC. Each format was translated into a form, which was used at each patient-physician consultation. There were five forms in this study and each patient was exposed to the one appropriate to their present SOC with respect to diet, exercise and medication use. These forms were used as checklists for the physician to ensure all the sections of the consultation were attended to during the visit.<br>CG: Routine care	General practitioner	Face-to-face<br>At 48 weeks	Clinical outcomes:<br>HbA1c<br>Body mass index<br>Blood pressure<br>Plasma urea and creatinine<br>Total cholesterol<br>Triglycerides<br>Blood glucose (random)<br>Humanistic outcomes:<br>Patients' readiness to change.
**Richards et al. (62)**	PHASE: a randomized, controlled trial of supervised self-help cognitive behavioral therapy in primary care<br>2003, UK	RCT<br>139 participants<br>(IG: 75; CG: 64)	Anxiety/ depression	IG: PN-facilitated self-help. Practice nurses assisted patients in using a self-help book "Managing Anxiety and Depression" a booklet developed for primary care, based on CBT techniques. PNs assisted patients in using the booklet both within and between sessions. The program comprised up to three sessions based on a previous trial evaluating a 'two-plus-one' session model. The initial two sessions offered 1 week apart, focused on familiarization with the booklet and applying it to samples of the patients' problems. Patients then used the booklet at home and were offered a third review appointment ('plus-one') 3 months later.<br>CG: Usual care	Nurse	Face-to-face<br>Weekly for 2 weeks, then at 3 months (total 3 sessions)	Clinical outcomes:<br>NA<br>Humanistic outcomes:<br>HRQOL<br>Depth of relationship; professional care and perceived time (using consultation satisfaction questionnaire)
**Rosemann et al. (63)**	Case management of arthritis patients in primary care: a cluster-randomized controlled trial	c-RCT<br>75 practices<br>1021 participants	Osteoarthritis	IG: GPs participated in 2 peer group meetings. GPs received written materials for patients: a leaflet providing information about the cause and the treatment possibilities as well as coping strategies. The leaflets also contained contact addresses for the 2 largest self-help groups for patients. GPs also	General Practitioner	Telephone<br>4-weekly	Clinical outcomes:<br>NA<br>Humanistic outcomes:

	2007, Germany	(IG (1): 345; IG (2): 344; CG: 332)		received booklets and audio CDs with a detailed exercise program and were asked to provide these materials to every included patient. In intervention group II, a practice nurse conducted additional case management via telephone.<br>CG: NA			HRQOL (AIMS2-SF questionnaire)<br>Physical activity
**Smit et al. (64, 65)**	Enhanced treatment for depression in primary care: first year results on compliance, self-efficacy, the use of antidepressants and contacts with the primary care physician<br>2005, The Netherlands	RCT<br>267 participants<br>(IG (1): 112; IG (2): 39; IG (3) 44; CG: 72)	Depression	IG (1): The Depression Recurrence Prevention (DRP) Program consisted of three individual face-to-face sessions with a trained prevention specialist, followed by four telephone contacts per year. DRP is a structured psycho-educational self-management intervention. Education on self-care management of depression is an integral part of the program. Prior to the first session, patients were handed a book and corresponding videotape containing information about depression, treatment options, relapse prevention and self-management strategies, and a two-page instruction booklet to prepare for the first session. In the first session, the prevention specialist gave an overview of the DRP-program. The potential benefits of self-monitoring of depressive symptoms and various stress reduction strategies were introduced and discussed. In the second session, the personal Recurrence Prevention Plan was prepared, with special attention to self-care and what could be learned from the patient's earlier episodes. Socializing and the scheduling of pleasant activities such as sports were encouraged. At the third and final session, depression specialist and patient drew up the final Prevention Plan, with the following topics: personal warning signs; stress reduction strategies; an 'Emergency-plan', with the steps that the patient was planning to take once s/he feared a relapse or recurrence; and a medication plan for patients using antidepressants. During the first phase of the DRP-Program, the primary care physician regularly received written feedback about patient cooperation and progress, and about medication use including side effects. After the last session, a copy of the patients'	Nurse<br>Psychologist	IG (1):<br>Face-to-face<br>3 individual sessions<br>Telephone<br>4 phone calls yearly for 3 years<br>IG (3):<br>Face-to-face<br>Weekly CBT for 12 weeks<br>3 individual sessions<br>Telephone<br>4 phone calls yearly for 3 years	Clinical outcomes:<br>Use of antidepressant medication<br>Humanistic outcomes:<br>Patient evaluation of the information and care received for depression<br>Effects on perceived self-efficacy

				Prevention Plan was sent to the physician with an accompanying letter in which specific elements of the plan were highlighted.<br><br>IG (2): PC and DRP. The PC+DRP group was offered one 1-hour visit with one of two available psychiatrists prior to the DRP-intervention. The PCP provided the psychiatrist with information about the patients' health and treatment status. Afterwards, the psychiatrist reported and discussed his diagnostic findings and treatment advice with the PCP. A copy of this report was also made available to the prevention specialist.<br><br>IG (3): CBT and DRP. The CBT+DRP group was offered 12 weakly one-hour sessions of CBT treatment. The DRP-Program started after the final CBT session. The CBT-therapist informed the prevention specialist about the main themes that the CBT had addressed, and the progress achieved. To reinforce concepts and CBT techniques and to monitor their adherence to the protocol, regular supervision sessions were held.<br><br>CG: Usual care			
**Striegel-Moore et al. (66, 67)**	Cognitive Behavioral Guided Self-Help for the Treatment of Recurrent Binge Eating<br><br>2010, United States	RCT<br><br>123 participants<br><br>(IG: 59; CG: 64)	Recurrent Binge Eating	IG: Following randomization, all participants were mailed a flyer detailing relevant health plan sponsored services, such as regularly offered series of classes focused on non-diet approaches to health living and eating. Patients were also encouraged to contact their primary care physician for other potentially appropriate services within the health plan including visits with a nutritionist or mental health provider. The intervention group additionally received 8 sessions implemented over a 12-week period. The treatment was based on Fairburn's "Overcoming Binge Eating" (1995). The book's first part provides user-friendly information about binge eating; the second part comprised a six-step self-help program. The primary focus was on developing a regular pattern of moderate eating using self-monitoring, self-control strategies, and problem	Therapists (master's level)	Face-to-face<br><br>Weekly for 4 weeks, then fortnightly (total 8 sessions)	Clinical outcomes:<br><br>Abstinence from binge eating<br><br>Humanistic outcomes:<br><br>Eating related psychopathology<br>Psychosocial functioning

				solving. To promote maintenance of behavioral change, relapse prevention was emphasized.<br><br>CG: Treatment as usual			
**Sturt et al. (68)**	Effects of the Diabetes Manual 1:1 structured education in primary care<br><br>2008, UK	c-RCT<br><br>48 practices<br><br>245 participants<br><br>(IG: 114; CG: 131)	Diabetes	IG: Practices nurses held a 15-minute face-to-face consultation to introduce the 12-week Diabetes Manual program. Patients worked independently through the workbook. Patient workbook was recommended at 1 hour per day over 12 weeks. Topics included diabetes facts/ metabolism/ goal setting and evaluation/ exercise/ nutrition/ blood glucose monitoring/ weight loss/ smoking cessation/ tests/ complications/ medication/ stress, anxiety and depression/ cholesterol/ quizzes to self-evaluate workbook topics/ other people's stories/ self-assessment record sheets to encourage personal evaluation of current and new behaviours. A relaxation audiotape was provided, and the patient was encouraged within the workbook to use it and to explore alternative relaxation methods. A question/ answer audiotape was provided mirroring discussion between a general practitioner and patient used as a brief introduction to diabetes and its management. Practice nurse telephone support was provided to assess goal progress, promotion of self-evaluation and re-negotiation, offer support and problem solve.<br><br>CG: Usual care	Nurse	Telephone<br><br>1, 5, 11 weeks	Clinical outcomes:<br><br>HbA1c<br>Cardiovascular risk factors<br><br>Humanistic outcomes:<br><br>Diabetes-related distress<br>Confidence to self-care measured
**Tiessen et al. (69, 70)**	Randomized controlled trial on cardiovascular risk management by practice nurses supported by self-monitoring in primary care<br><br>2012, The Netherlands	RCT<br><br>201 participants<br><br>(IG: 105; CG: 96)	Cardiovascular disease or diabetes	IG: All patients received counselling regarding cardiovascular risk from practice nurses trained in MI techniques and in the intervention group this counselling was based on self-monitoring results (pedometer, weighing scale and/ or blood pressure device). Treatment for all present risk factors was pro-actively offered. The order in which the treatments for the different risk factors were started depended on preference and SOC of the participant. Adapted MI was used to help participants recognize and change unhealthy behaviour. If applicable, quitting smoking was advised as the first treatment goal. Treatment for	Nurse	Face-to-face<br><br>Variable number of sessions over 12 months	Clinical outcomes:<br><br>Anthropometric data<br>Changes in medication and medical history<br>Fasting blood glucose; lipids and creatinine<br>Number and duration of visits

				all risk factors that the participant was motivated for working on, had to start within three months. In case of several risk factors, these treatments could be combined within one visit. (1) Overweight: Intensive counselling and feedback on energy intake and expenditure, supported by food diary, home weight scale, step diary and pedometer. (2) Smoking: Intensive counselling and feedback based on SOC, Minimal Intervention Strategy and Dutch GP guideline. (3) Physical Inactivity: Intensive counselling and feedback on increasing physical activity, supported by step diary and pedometer. (4) Hypertension/Hypercholesterolemia: same as control group except feedback based on home measurements.<br><br>CG: For the control group, follow-up visits were planned according to the Dutch GP guideline in case of hypertension and/or hypercholesterolemia. Medication adjustments were made by the practice nurses under supervision of the GP. For each visit the practice nurses filled in a step by step treatment plan based on the Dutch GP guideline.			Humanistic outcomes:<br><br>Smoking behaviour<br>Use of self-monitoring
**van Dijk-de Vries et al. (71)**	Lessons learnt from a cluster-randomized trial evaluating the effectiveness of Self-Management Support (SMS) delivered by practice nurses in routine diabetes care<br><br>2015, The Netherlands	c-RCT<br><br>77 practices<br><br>264 participants<br><br>(IG: 117; CG: 147)	T2DM	IG: SMS included a detection and follow-up phase. The detection phase of SMS started by exploring whether patients experienced problems in daily life. Patients who experienced problems of daily functioning and emotional health problems were offered consultations for SMS. These extra consultations delivered by PNs were aimed at supporting patients in their day-to-day management of diabetes and its emotional and social consequences. The intervention strategy derived from the principles of learning theory has been described elsewhere. PNs supported patients in the processes of defining problems and finding solutions themselves, by applying problem-solving and reattribution techniques. Problem solving consisted of seven stages that efficiently addressed problems and their possible solutions. The reattribution	Nurse	Face-to-face or telephone<br><br>3-monthly	Clinical outcomes:<br><br>Glycemic control<br><br>Humanistic outcomes:<br><br>Perceived effect of diabetes on daily functioning<br>Diabetes-related distress<br>QOL<br>Autonomy and participation<br>Self-efficacy<br>Self-management

				technique was applied to challenge patients to link feelings and cognition to consequent behaviour. Patients could use information from a diary in which they recorded symptoms, thoughts, worries, feelings, and behaviour. Both problem solving, and reattribution techniques were intended to result in action plans indicating how patients would achieve their personal goals.<br>CG: Usual care			
**Von Korff et al. (72)**	A trial of an activating intervention for chronic back pain in primary care and physical therapy settings<br>2005, United States	RCT<br>240 participants<br>(IG: 119; CG: 121)	Chronic back pain	IG: An initial 90-minute visit with a psychologist; identified and addressed patient fears about back pain; discussing the relationship between resuming normal activities and QOL; setting activity or exercise goals to enhance QOL; and developed an action plan to achieve the goal. The second visit with a physical therapist conducted a standardized mechanical examination of the back, discussed unresolved patient concerns identified in the initial visit, taught stretches and exercises relevant to the action plan, and offered guidance in overcoming barriers the patient had encountered in carrying out the action plan. The third visit focused on the action plan and exercises relevant to the action plan. After a 2-week interval, a fourth visit (30 minutes) with the psychologist reviewed progress, encouraged use of relaxation, and developed plans for sustaining progress, managing flare ups and resuming activities when a flare up occurred.<br>CG: Usual care	Psychologist<br>Physiotherapist	Face-to-face<br>4 sessions over 4-5 weeks<br>Telephone<br>2, 6, 12 and 24 months	Clinical outcomes:<br>Pain intensity<br>Chronic pain grade<br>Humanistic outcomes:<br>Fear avoidance beliefs<br>Mental health and social functioning
**Waite et al. (73)**	Cognitive behaviour therapy for low self-esteem: a preliminary randomized controlled trial in a primary care setting<br>2012, UK	RCT<br>22 participants<br>(IG: 11; CG: 11)	Low self esteem	IG: The treatment was based on Fennell's (1997, 1999, 2006) CBT protocol for overcoming low self-esteem and included four phases: 1. Individualized formulation, goal setting and psycho-education (sessions 1-2) 2. Learning skills to re-evaluate anxious and self-critical thoughts and beliefs through cognitive techniques and behavioral experiments (sessions 3-8) 3. Enhancing self-acceptance (sessions 4 - 8) 4. The development of more adaptive beliefs	Psychologist	Face-to-face<br>10 sessions over 11 weeks	Clinical outcomes:<br>Self-report measures of depression (Beck Depression Inventory-II)<br>Humanistic outcomes:

				and rules and planning for the future (sessions 7-10). As well as individual treatment sessions, all participants were given a three-part self-help workbook and were asked to read the chapters and complete the exercises to tie in with the associated therapy sessions.<br>CG: Wait list control			Self Esteem (Robson Self-concept Questionnaire)<br>Overall psychological functioning and wellbeing (Routine Evaluation Outcome Measure)
**Watkins et al. (74)**	Guided self-help concreteness training as an intervention for major depression in primary care: A Phase II randomized controlled trial<br>2012, UK	RCT<br>121 participants<br>(IG (1): 40; IG (2): 39; CG; 42)	Major depression	IG (1): Cognitive bias modification (CBM) training guided self-help intervention. During the initial session, the treatment rationale was explained, the psychologist provided psycho-education about depression, rumination and overgeneralization, and practiced training exercises with the patient. During the telephone sessions, feedback, guidance and encouragement was provided to ensure accurate use of the exercises, monitored progress and scheduled regular practice. The training exercises involved patients' identifying a recent mildly to moderately upsetting difficulty and working through standardized steps to facilitate concrete thinking: (1) using mental imagery to focus on sensory details during the difficult event, noticing what is specific about the event and the context in which it occurs; (2) noticing the process and sequence by which the difficult event unfolds (‘How did it happen?’), including warning signs and actions that may have influenced its outcome; (3) focusing on how to move forward by specifying the particular steps and behaviours to do next (Watkins, 2009; Watkins et al. 2009). The practice CD included (a) 30 min repeating the original training exercise; (b) a 7-min First Aid exercise in which concrete thinking is applied to difficulties in real time as they occur (practiced in the first telephone session); (c) a 7-min ‘absorption exercise’ in which concrete thinking is	Psychologist	Telephone<br>1 week after initial training, and fortnightly thereafter	Clinical outcomes:<br>Self-report depression measures<br>Humanistic outcomes:<br>NA

				used to enhance positive experiences (practiced in the second telephone session).<br><br>IG (2): Treatment as usual plus relaxation training guided self-help. In relaxation training, the training exercises involved progressive relaxation skills including tensing and relaxing muscle groups and slowing breathing. The practice CD included (a) a 30-min progressive relaxation exercise; (b) a 7-min First Aid exercise using relaxation; (c) a 7-min exercise in which patients practiced letting go of tension without prior tensing of muscles.<br><br>CG: Treatment as usual			
**Watson et al. (75)**	Evaluation of a self-management plan for chronic obstructive pulmonary disease<br><br>New Zealand, 1997	RCT<br><br>56 participants<br><br>(IG: 29; CG: 27)	COPD	IG: Practice nurses were educated about the use of the Action Plan and booklet by a senior nurse from the hospital respiratory outreach service. The PN then introduced subjects to the Action Plan and booklet. The GP also saw each subject and gave them a prescription for a course of oral prednisone and a broad spectrum antibiotic appropriate for self-administration during an exacerbation. No attempt was made to supervise the adequacy of the instruction given to subjects. The COPD Action Plan and booklet evaluated in this study were developed by staff from Canterbury Respiratory Services. The format for the Action Plan was modelled on the asthma action plan produced by the Asthma Foundation of New Zealand. Feedback from workshops with GPs and PNs, and interviews with patients led to modifications to the Action Plan. The booklet, entitled "A Guide to Living Positively with Chronic Obstructive Pulmonary Disease", was developed to be used in conjunction with the Action Plan. Existing patient education material was reviewed together with appropriate scientific literature. The topics included in the booklet were: stopping smoking; controlling breathlessness; exercise; daily activities made easier; diet; sleep; clearing mucus from the lungs; planning for the future; medications; oxygen; and contact details for	Nurses	Not specified	Clinical outcomes:<br><br>Respiratory status; prednisone use; antibiotic use; contact with GP, PN, hospital specialist, pharmacist<br><br>Humanistic outcomes:<br><br>HRQOL

				support services. Drafts of the booklet were circulated among patients, their families, respiratory health professionals and PNs for comment.<br><br>CG: Usual care			
**Williams et al. (76)**	Guided self-help cognitive behavioral therapy for depression in primary care: a randomized controlled trial<br><br>2013, UK	RCT<br><br>281 participants<br><br>(IG: 140; CG: 141)	Depression	IG: The first appointment focused on an introduction to the use of self-help materials. The patient was given a copy of Workbook 1 (‘‘Understanding depression’’) and instructed on how to use it. At session 2, the first workbook was reviewed before a joint decision identified additional 1–2 treatment workbooks to be used between sessions 2 and 3. These were chosen on the basis of the initial self-assessment in the Understanding depression workbook. At session 3, there was a final review of their progress. The relapse prevention workbook and up to one or two additional workbooks were also offered at this final appointment. The workbooks aimed to communicate key CBT principles in a low jargon way. Case examples, illustrations, text and interactive worksheets encouraged users to self-assess, and then choose which topics (workbooks) they would work on. Each workbook included a Putting into Practice (homework) plan to encourage application in the reader’s own life. The choice of workbooks followed a core/options approach where the initial workbook (self-assessment) helped identify what problem areas the person wished to work on. In the final support session, the focus was on the Planning for the Future (relapse prevention) workbook. At any time during treatment, patients could arrange to see their doctor or other health care practitioner as normal. The intervention by the psychology graduate was only to support the use of the self-help materials using a written support protocol and ‘‘advice’’ separate from the intervention was not offered. The GP was informed that the participant had been seen and discharged at the end of GCBT-SH support. The support protocol focused on using and applying one	General practitioner<br><br>Psychologist	Face to face<br><br>Weekly to fortnightly (total 3-4 sessions)	Clinical outcomes:<br><br>Psychological symptoms<br><br>Humanistic outcomes:<br><br>Social functioning Acceptability of the intervention (using the Client Satisfaction Questionnaire-CSQ)

				to two workbooks per week. The support worker encouraged the participant to read, answer questions and plan how to put what was being learned into practice. Each session allowed progress or barriers to progress to be reviewed and plans to overcome these barriers to be discussed.<br><br>CG: Treatment as usual			
**Wood-Baker et al. (77)**	Written action plans in chronic obstructive pulmonary disease increase appropriate treatment for acute exacerbations<br><br>2006, Australia	c-RCT<br><br>139 participants<br><br>(IG: 67; CG: 72)	COPD	IG: All participants received a COPD information booklet and an individual educational session with a nurse experienced in managing respiratory disease. The nurse covered a range of topics including basic pathology of COPD, smoking cessation, immunization, nutrition, exercise, sputum clearance techniques, breathing control, stress management, medications, inhaler use and community support services. Participants in the intervention group were provided with a written self-management action plan, which was developed in consultation with their GP. The self-management plan listed the patient's maintenance medications and provided an individualized action plan based on the early recognition of symptoms associated with exacerbations of COPD. All participants in the action plan group were encouraged to make early contact with their GP during an exacerbation.<br><br>CG: Routine care	Nurse<br><br>General Practitioner	Face-to-face<br><br>6, 12 months<br><br>Telephone<br><br>3, 9 months	Clinical outcomes:<br><br>Use of antibiotics<br>Short courses of oral steroids<br>Smoking status<br><br>Humanistic outcomes:<br><br>HRQOL
**Yardley et al. (78)**	Effectiveness of primary care-based vestibular rehabilitation for chronic dizziness<br><br>2004, UK	RCT<br><br>170 participants<br><br>(IG: 83; CG: 87)	Chronic dizziness	IG: The nurse taught the patient exercises to be carried out daily at home, with the support of a treatment booklet. Nurses explained the rationale for vestibular rehabilitation, described and took the patient through the set of standard head and eye exercises, asked the patient to identify and record in the booklet, advised the patient how to monitor recovery using standardized exercises and dizziness ratings recorded weekly in the booklet; the nurse then taught the patient to tailor the intensity and difficulty of the exercises he or she carries out to their stage of recovery, helped the patient to select daily activities	Nurse	Telephone<br><br>At 1 and 3 weeks	Clinical outcomes:<br><br>Self-reported spontaneous and provoked symptoms of dizziness<br>Objective measurement of postural stability with eyes open and eyes closed

				to encourage physical and psychological adaptation in everyday situations, suggested additional customized exercises to treat particular forms of dizziness or imbalance, provided advice on how to anticipate and cope with obstacles to adherence. The patient carried out exercises and activities daily at home; the patient monitored and adjusted the program as needed.<br><br>CG: Usual care			Humanistic outcomes:<br><br>Dizziness related QOL<br>Anxiety and depression (Hospital Anxiety and Depression Scale)<br>QOL (scores on the physical functioning scale of the Medical Outcomes Study Short Form-36 quality of life questionnaire)
**Zimmermann et al. (79)**	Collaborative nurse-led self-management support for primary care patients with anxiety, depressive or somatic symptoms: Cluster-randomized controlled trial (findings of the SMADS study)<br><br>2016, Germany	c-RCT<br><br>20 practices<br><br>325 participants<br><br>(IG: 191; CG: 134)	Anxiety<br><br>Depression	IG: Case management and counselling techniques to promote self-management for patients. In cooperation with the patients, they developed specific objectives to be achieved over the course of the trial. Together, they decided on a hierarchy of goals, from smaller to larger ones, consented and recorded in written form. Subsequently, the nurses and the patients developed strategies on how to achieve these goals. The planning of the measures and concrete self-management support took place in close consultation with GPs. After reaching an agreement at the first session, further appointments were scheduled. Over the course of the trial, nurses could use the nine modules of intervention to support their patients or offer low-threshold, behavioral modules: problem-solving techniques, relaxation exercises or strengthening self-confidence activities – all promoting better self-care, i.e. improving self-management. The counselling process ended with a final interview in order to get patients' feedback, check goal attainment and preview further	Nurse	Face-to-face<br><br>Sessions delivered over 12 months	Clinical outcomes:<br><br>NA<br><br>Humanistic outcomes:<br><br>Change in self-efficacy

				developments. Nurses regularly met with the study GP (EP; GP and psychotherapist) for joint discussions.<br><br>CG: Routine care			

Abbreviations: BMI: body mass index; CBM: cognitive bias modification; CBT: cognitive behaviour therapy; CG: control group; CHD: coronary heart disease; COPD: chronic obstructive pulmonary disease; c-RCT: cluster randomized controlled trial; DCRS: diabetes care record sheet; DRP: depression recurrence prevention; EQ-5D: EuroQoL-5D; GPs: general practitioners; HbA1c: glycated haemoglobin; HRQOL: health-related quality of life; IBS: irritable bowel syndrome; IG: intervention group; MI: motivational interviewing; NA: not available; NCMs: nurse case managers; NP: nurse practitioners; OT: occupational therapist; PCPs: primary care professionals; PCST: pain coping skills training; PEFR: peak expiratory flow rate; PNs: practice nurses; QOL: quality of life; RCT: randomized controlled trial; SOC: stage of change; SMS: self-management support; T2DM: type 2 diabetes mellitus; UC: usual care; UK: United Kingdom

References

1. Adachi M, Yamaoka K, Watanabe M, Nishikawa M, Kobayashi I, Hida E, et al. Effects of lifestyle education program for type 2 diabetes patients in clinics: a cluster randomized controlled trial. BMC public health. 2013;13:467.
2. Banasiak S, Paxton S, Hay P. Guided self-help for bulimia nervosa in primary care: a randomized controlled trial. Psychological medicine. 2005;35(9):1283-94.
3. Barbanel D, Eldridge S, Griffiths C. Can a self-management programme delivered by a community pharmacist improve asthma control? A randomised trial. Thorax. 2003;58(10):851-4.
4. Barley E, Walters P, Haddad M, Phillips R, Achilla E, McCrone P, et al. The UPBEAT nurse-delivered personalized care intervention for people with coronary heart disease who report current chest pain and depression: a randomised controlled pilot study. PloS one. 2014;9(6):e98704.
5. Bartels S, Pratt S, Mueser K, Naslund J, Wolfe R, Santos M, et al. Integrated IMR for psychiatric and general medical illness for adults aged 50 or older with serious mental illness. Psychiatric services (Washington, DC). 2014;65(3):330-7.
6. Bischoff E, Akkermans R, Bourbeau J, van Weel C, Vercoulen J, Schermer T. Comprehensive self management and routine monitoring in chronic obstructive pulmonary disease patients in general practice: randomised controlled trial. BMJ : British Medical Journal. 2012;345.
7. Broderick J, Keefe F, Bruckenthal P, Junghaenel D, Schneider S, Schwartz J, et al. Nurse practitioners can effectively deliver pain coping skills training to osteoarthritis patients with chronic pain: A randomized, controlled trial. Pain. 2014;155(9):1743-54.
8. Browning C, Chapman A, Yang H, Liu S, Zhang T, Enticott J, et al. Management of type 2 diabetes in China: the Happy Life Club, a pragmatic cluster randomised controlled trial using health coaches. BMJ open. 2016;6(3):e009319.
9. Browning C, Yang H, Zhang T, Chapman A, Liu S, Enticott J, et al. Implementing a Chronic Disease Self-Management Program into China: The Happy Life Club™. Frontiers in public health. 2014;2:181.
10. Chalder T, Wallace P, Wessely S. Self-help treatment of chronic fatigue in the community: A randomized controlled trial. British Journal of Health Psychology. 1997;2(3):189-97.
11. Cherkin D, Deyo R, Street J, Hunt M, Barlow W. Pitfalls of patient education. Limited success of a program for back pain in primary care. Spine. 1996;21(3):345-55.
12. Clark M, Hampson S. Implementing a psychological intervention to improve lifestyle self-management in patients with Type 2 diabetes. Patient education and counseling. 2001;42(3):247-56.
13. Clark M, Hampson S, Avery L, Simpson R. Effects of a tailored lifestyle self-management intervention in patients with Type 2 diabetes. British Journal of Health Psychology. 2004;9:365-79.
14. Blinkhorn A. Influencing patients' oral hygiene behaviour. Evidence-based dentistry. 2010;11(2):41.
15. Clarkson J, Young L, Ramsay C, Bonner B, Bonetti D. How to influence patient oral hygiene behavior effectively. Journal of dental research. 2009;88(10):933-7.
16. Doucette W, Witry M, Farris K, McDonough R. Community pharmacist-provided extended diabetes care. The Annals of pharmacotherapy. 2009;43(5):882-9.
17. Porcheret M, Grime J, Main C, Dziedzic K. Developing a model osteoarthritis consultation: a Delphi consensus exercise. BMC musculoskeletal disorders. 2013;14:25.
18. Dziedzic KS, Healey EL, Porcheret M, Ong BN, Main CJ, Jordan KP, et al. Implementing the NICE osteoarthritis guidelines: a mixed methods study and cluster randomised trial of a model osteoarthritis consultation in primary care--the Management of OsteoArthritis In Consultations (MOSAICS) study protocol. Implement Sci. 2014;9:95.
19. Dziedzic KS, Healey EL, Porcheret M, Afolabi EK, Lewis M, Morden A, et al. Implementing core NICE guidelines for osteoarthritis in primary care with a model consultation (MOSAICS): a cluster randomised controlled trial. Osteoarthritis and cartilage. 2018;26(1):43-53.
20. Efraimsson E, Hillervik C, Ehrenberg A. Effects of COPD self-care management education at a nurse-led primary health care clinic. Scandinavian journal of caring sciences. 2008;22(2):178-85.
21. Eikelenboom N, van Lieshout J, Jacobs A, F. V, Lacroix J, van Halteren A, et al. Effectiveness of personalised support for self-management in primary care: a cluster randomised controlled trial. The British journal of general practice : the journal of the Royal College of General Practitioners. 2016;66(646):e354-61.
22. Eikelenboom N, van Lieshout J, Wensing M, Smeele I, Jacobs A. Implementation of personalized self-management support using the self-management screening questionnaire SeMaS; a study protocol for a cluster randomized trial. Trials. 2013;14:336.

23. Farmer A, Wade A, Goyder E, Yudkin P, French D, Craven A, et al. Impact of self monitoring of blood glucose in the management of patients with non-insulin treated diabetes: open parallel group randomised trial. BMJ (Clinical research ed). 2007;335(7611):132.
24. Simon J, Gray A, Clarke P, Wade A, Neil A, Farmer A. Cost effectiveness of self monitoring of blood glucose in patients with non-insulin treated type 2 diabetes: economic evaluation of data from the DiGEM trial. BMJ (Clinical research ed). 2008;336(7654):1177-80.
25. Farmer A, Wade A, French D, Simon J, Yudkin P, Gray A, et al. Blood glucose self-monitoring in type 2 diabetes: a randomised controlled trial. Health technology assessment (Winchester, England). 2009;13(15):iii-iv, ix-xi, 1-50.
26. Ferrone M, Masciantonio MG, Malus N, Stitt L, O'Callahan T, Roberts Z, et al. The impact of integrated disease management in high-risk COPD patients in primary care. npj Primary Care Respiratory Medicine. 2019;29(1):8.
27. Fortin M, Chouinard M-C, Dubois M-F, Bélanger M, Almirall J, Bouhali T, et al. Integration of chronic disease prevention and management services into primary care: a pragmatic randomized controlled trial (PR1MaC). CMAJ Open. 2016;4(4):E588-E98.
28. Contant É, Loignon C, Bouhali T, Almirall J, Fortin M. A multidisciplinary self-management intervention among patients with multimorbidity and the impact of socioeconomic factors on results. BMC Family Practice. 2019;20(1):53.
29. Freund K, Lous J. The effect of preventive consultations on young adults with psychosocial problems: a randomized trial. Health education research. 2012;27(5):927-45.
30. Friedberg F, Napoli A, Coronel J, Adamowicz J, Seva V, Caikauskaite I, et al. Chronic fatigue self-management in primary care: a randomized trial. Psychosomatic medicine. 2013;75(7):650-7.
31. Meng H, Friedberg F, Castora-Binkley M. Cost-effectiveness of chronic fatigue self-management versus usual care: a pilot randomized controlled trial. BMC family practice. 2014;15:184.
32. Gabbay R, Lendel I, Saleem T, Shaeffer G, Adelman A, Mauger D, et al. Nurse case management improves blood pressure, emotional distress and diabetes complication screening. Diabetes research and clinical practice. 2006;71(1):28-35.
33. Gabbay R, Anel-Tiangco R, Dellasega C, Mauger D, Adelman A, Van Horn D. Diabetes nurse case management and motivational interviewing for change (DYNAMIC): Results of a 2-year randomized controlled pragmatic trial. Journal of Diabetes. 2013;5(3):349-57.
34. Goudswaard A, Stolk R, Zuithoff N, de Valk H, Rutten G. Long-term effects of self-management education for patients with Type 2 diabetes taking maximal oral hypoglycaemic therapy: a randomized trial in primary care. Diabetic medicine : a journal of the British Diabetic Association. 2004;21(5):491-6.
35. Grilo C, White M, Gueorguieva R, Barnes R, Masheb R. Self-help for binge eating disorder in primary care: a randomized controlled trial with ethnically and racially diverse obese patients. Behaviour research and therapy. 2013;51(12):855-61.
36. Heitkemper M, Jarrett M, Levy R, Cain K, Burr R, Feld A, et al. Self-management for women with irritable bowel syndrome. Clinical Gastroenterology and Hepatology. 2004;2(7):585-96.
37. Hill K, Mangovski-Alzamora S, Blouin M, Guyatt G, Heels-Ansdell D, Bragaglia P, et al. Disease-specific education in the primary care setting increases the knowledge of people with chronic obstructive pulmonary disease: A randomized controlled trial. Patient education and counseling. 2010;81(1):14-8.
38. Hoffmann W, Herzog B, Muhlig S, Kayser H, Fabian R, Thomsen M, et al. Pharmaceutical care for migraine and headache patients: a community-based, randomized intervention. The Annals of pharmacotherapy. 2008;42(12):1804-13.
39. Huang M, Hsu C, Wang H, Shin S. Prospective randomized controlled trial to evaluate effectiveness of registered dietitian-led diabetes management on glycemic and diet control in a primary care setting in Taiwan. Diabetes care. 2010;33(2):233-9.
40. Hsu C, Tai T. Long-term glycemic control by a diabetes case-management program and the challenges of diabetes care in Taiwan. Diabetes research and clinical practice. 2014;106(S2):S328-S32.
41. Ismail M, Teng C, Omar M, Ho B, Kusiar Z, Hasim R. Usage of glucometer is associated with improved glycaemic control in type 2 diabetes mellitus patients in Malaysian public primary care clinics: an open-label, randomised controlled trial. Singapore Med J. 2013;54(7):391-5.
42. Jaipakdee J, Jiamjarasrangsi W, Lohsoonthorn V, Lertmaharit S. Effectiveness of a self-management support program for Thais with type 2 diabetes: Evaluation according to the RE-AIM framework. Nursing & Health Sciences. 2015;17(3):362-9.
43. Kennedy A, Bower P, Reeves D, Blakeman T, Bowen R, Chew-Graham C, et al. Implementation of self management support for long term conditions in routine primary care settings: cluster randomised controlled trial. BMJ : British Medical Journal. 2013;346:f2882.

44. Kennedy A, Chew-Graham C, Blakeman T, Bowen A, Gardner C, Protheroe J, et al. Delivering the WISE (Whole Systems Informing Self-Management Engagement) training package in primary care: learning from formative evaluation. Implement Sci. 2010;5:7-.
45. McGeoch G, Willsman K, Dowson C, Town G, Frampton C, McCartin F, et al. Self-management plans in the primary care of patients with chronic obstructive pulmonary disease. Respirology. 2006;11(5):611-8.
46. McLean W, Gillis J, Waller R. The BC Community Pharmacy Asthma Study: A study of clinical, economic and holistic outcomes influenced by an asthma care protocol provided by specially trained community pharmacists in British Columbia. Canadian respiratory journal. 2003;10(4):195-202.
47. Mehuys E, Van Bortel L, De Bolle L, Van Tongelen I, Annemans L, Remon J, et al. Effectiveness of pharmacist intervention for asthma control improvement. The European respiratory journal. 2008;31(4):790-9.
48. Mehuys E, Van Bortel L, De Bolle L, Van Tongelen I, Annemans L, Remon J, et al. Effectiveness of a community pharmacist intervention in diabetes care: a randomized controlled trial. Journal of clinical pharmacy and therapeutics. 2011;36(5):602-13.
49. Meland E, Laerum E, Ulvik R. Effectiveness of two preventive interventions for coronary heart disease in primary care. Scandinavian journal of primary health care. 1997;15(1):57-64.
50. Meland E, Maeland J, Laerum E. The importance of self-efficacy in cardiovascular risk factor change. Scandinavian journal of public health. 1999;27(1):11-7.
51. Mitchell K, Johnson-Warrington V, Apps L, Bankart J, Sewell L, Williams J, et al. A self-management programme for COPD: a randomised controlled trial. The European respiratory journal. 2014;44(6):1538-47.
52. Dritsaki M, Johnson-Warrington V, Mitchell K, Singh S, Rees K. An economic evaluation of a self-management programme of activity, coping and education for patients with chronic obstructive pulmonary disease. Chron Respir Dis. 2016;13(1):48-56.
53. Morgan M, Dunbar J, Reddy P, Coates M, Leahy R. The TrueBlue study: is practice nurse-led collaborative care effective in the management of depression for patients with heart disease or diabetes? BMC family practice. 2009;10:46.
54. Morgan M, Coates M, Dunbar J, Reddy P, Schlicht K, Fuller J. The TrueBlue model of collaborative care using practice nurses as case managers for depression alongside diabetes or heart disease: a randomised trial. BMJ open. 2013;3(1).
55. Moss-Morris R, McAlpine L, Didsbury L, Spence M. A randomized controlled trial of a cognitive behavioural therapy-based self-management intervention for irritable bowel syndrome in primary care. Psychological medicine. 2010;40(1):85-94.
56. Chilcot J, Moss-Morris R. Changes in illness-related cognitions rather than distress mediate improvements in irritable bowel syndrome (IBS) symptoms and disability following a brief cognitive behavioural therapy intervention. Behaviour research and therapy. 2013;51(10):690-5.
57. Murphy A, Cupples M, Smith S, Byrne M, Byrne MC, Newell J. Effect of tailored practice and patient care plans on secondary prevention of heart disease in general practice: cluster randomised controlled trial. BMJ (Clinical research ed). 2009;339:b4220.
58. Murphy A, Cupples M, Smith S, Byrne M, Leathem C, Byrne M. The SPHERE Study. Secondary prevention of heart disease in general practice: protocol of a randomised controlled trial of tailored practice and patient care plans with parallel qualitative, economic and policy analyses. [ISRCTN24081411]. Current controlled trials in cardiovascular medicine. 2005;6:11.
59. Gillespie P, O'Shea E, Smith S, Cupples M, Murphy A. A comparison of medical records and patient questionnaires as sources for the estimation of costs within research studies and the implications for economic evaluation. Family practice. 2016.
60. Olry de Labry Lima A, Bermudez Tamayo C, Pastor Moreno G, Bolivar Munoz J, Ruiz Perez I, Johri M, et al. Effectiveness of an intervention to improve diabetes self-management on clinical outcomes in patients with low educational level. Gaceta sanitaria / SESPAS. 2016.
61. Partapsingh V, Maharaj R, Rawlins J. Applying the Stages of Change model to Type 2 diabetes care in Trinidad: A randomised trial. Journal of Negative Results in BioMedicine. 2011;10(1).
62. Richards A, Barkham M, Cahill J, Richards D, Williams C, Heywood P. PHASE: a randomised, controlled trial of supervised self-help cognitive behavioural therapy in primary care. The British journal of general practice : the journal of the Royal College of General Practitioners. 2003;53(495):764-70.
63. Rosemann T, Joos S, Laux G, Gensichen J, Szecsenyi J. Case management of arthritis patients in primary care: a cluster-randomized controlled trial. Arthritis and rheumatism. 2007;57(8):1390-7.
64. Smit A, Tiemens B, Ormel J, Kluiter H, Jenner J, van de Meer K, et al. Enhanced treatment for depression in primary care: first year results on compliance, self-efficacy, the use of antidepressants

and contacts with the primary care physician. Primary Care & Community Psychiatry. 2005;10(2):39-49.
65. Stant A, TenVergert E, Kluiter H, Conradi H, Smit A, Ormel J. Cost-effectiveness of a psychoeducational relapse prevention program for depression in primary care. The journal of mental health policy and economics. 2009;12(4):195-204.
66. Striegel-Moore R, Wilson G, DeBar L, Perrin N, Lynch F, Rosselli F, et al. Cognitive Behavioral Guided Self-Help for the Treatment of Recurrent Binge Eating. Journal of consulting and clinical psychology. 2010;78(3):312-21.
67. DeBar L, Striegel-Moore R, Wilson G, Perrin N, Yarborough B, Dickerson J, et al. Guided Self-Help Treatment for Recurrent Binge Eating: Replication and Extension. Psychiatric Services. 2011;62(4):367-73.
68. Sturt J, Whitlock S, Fox C, Hearnshaw H, Farmer A, Wakelin M, et al. Effects of the Diabetes Manual 1:1 structured education in primary care. Diabetic medicine : a journal of the British Diabetic Association. 2008;25(6):722-31.
69. Tiessen A, Smit A, Broer J, Groenier K, van der Meer K. Randomized controlled trial on cardiovascular risk management by practice nurses supported by self-monitoring in primary care. BMC family practice. 2012;13:90.
70. Tiessen A, Vermeulen K, Broer J, Smit A, van der Meer K. Cost-effectiveness of cardiovascular risk management by practice nurses in primary care. BMC public health. 2013;13:148.
71. van Dijk-de Vries A, van Bokhoven M, Winkens B, Terluin B, Knottnerus J, van der Weijden T, et al. Lessons learnt from a cluster-randomised trial evaluating the effectiveness of Self-Management Support (SMS) delivered by practice nurses in routine diabetes care. BMJ open. 2015;5(6):e007014.
72. Von Korff M, Balderson B, Saunders K, Miglioretti D, Lin E, Berry S, et al. A trial of an activating intervention for chronic back pain in primary care and physical therapy settings. Pain. 2005;113(3):323-30.
73. Waite P, McManus F, Shafran R. Cognitive behaviour therapy for low self-esteem: a preliminary randomized controlled trial in a primary care setting. Journal of behavior therapy and experimental psychiatry. 2012;43(4):1049-57.
74. Watkins E, Taylor R, Byng R, Baeyens C, Read R, Pearson K, et al. Guided self-help concreteness training as an intervention for major depression in primary care: a Phase II randomized controlled trial. Psychological medicine. 2012;42(7):1359-71.
75. Watson PB, Town GI, Holbrook N, Dwan C, Toop LJ, Drennan CJ. Evaluation of a self-management plan for chronic obstructive pulmonary disease. The European respiratory journal. 1997;10(6):1267-71.
76. Williams C, Wilson P, Morrison J, McMahon A, Walker A, Allan L, et al. Guided self-help cognitive behavioural therapy for depression in primary care: a randomised controlled trial. PloS one. 2013;8(1):e52735.
77. Wood-Baker R, McGlone S, Venn A, Walters E. Written action plans in chronic obstructive pulmonary disease increase appropriate treatment for acute exacerbations. Respirology. 2006;11(5):619-26.
78. Yardley L, Donovan-Hall M, Smith H, Walsh B, Mullee M, Bronstein A. Effectiveness of primary care-based vestibular rehabilitation for chronic dizziness. Annals of internal medicine. 2004;141(8):598-605.
79. Zimmermann T, Puschmann E, van den Bussche H, Wiese B, Ernst A, Porzelt S, et al. Collaborative nurse-led self-management support for primary care patients with anxiety, depressive or somatic symptoms: Cluster-randomised controlled trial (findings of the SMADS study). International journal of nursing studies. 2016;63:101-11.
