# Supplementary material for: Helping patients help themselves: A systematic review of self-management support strategies in primary health care practice
Source: PLoS One. 2019 Aug 1;14(8):e0220116. doi: 10.1371/journal.pone.0220116 (PMC6675068; doi:10.1371/journal.pone.0220116)
Supplement: S4 Table — (PDF) [file pone.0220116.s005.pdf]

S4 Table. Summary of findings and extracted outcomes

Reported outcomes	Method of assessment	Findings in (c)-RCTs
**Self-efficacy**		
Changes in perceived self-efficacy	DMSES	(+) Sturt et al. (1)
	Questionnaire (Likert 7-point scale)	(+) Clarkson et al. (2, 3)
	Arthritis Self-Efficacy Scale (8 items) * adapted from Lorig et al. 1989	(+) (post-treatment); NS (6 months); NS (12 months) Broderick et al. (4)
	Arthritis Self-Efficacy pain subscale	NS Dziedzic et al. (5-7)
	GSE	(+) Zimmermann et al. (8)
	GSES-12	NEA Barley et al. (9)<br>NS van Dijk-de Vries et al. (10)
	Questionnaire (28 item)	NS Hoffmann et al. (11)
	Self-administered questionnaire	NS Meland et al. (12, 13)
	PRAISE	NS Mitchell et al. (14, 15)
	CDMSES	NS Browning et al. (16, 17)
	COPD self-efficacy scale	NS Bischoff et al. (18)
	DSES	NS Smit et al. (19, 20)
	Short form healthcare climate questionnaire	NS Kennedy et al. (21, 22)
	Managing Chronic Disease 6-Item Scale	(+) Fortin et al. (23, 24)
Level of patient activation	PAM-13	NS Eikelenboom et al. (25, 26)
Self-management and patient enablement	Patient Enablement Instrument (PEI)	(+) Dziedzic et al. (5-7)
**Quality of life**		
Change in HRQOL	SGRQ	(+) Efraimsson et al. (27)<br>NS Wood-Baker et al. (28)<br>NS McGeoch et al. (29)<br>NS Watson et al. (30)
	Dizziness Handicap Inventory (25 items)	(+) Yardley et al. (31)
	WSAS	(+) Moss-Morris et al. (32, 33)<br>(+) Striegel-Moore et al. (34, 35)
	IBSQOL	(+) Heitkemper et al. (36)
	Brief clinical inventory category	(+) Jaipakdee et al. (37)
	AIMS2-SF	NS (Group 1); (+) (Group 2 symptom scale, the lower limb scale, and the social scale) Rosemann et al. (38)<br>NS Broderick et al. (4)

	SF-12	NS (physical health); (+) (mental health) Freund et al. (39)<br>NEA Barley et al. (9)<br>NS van Dijk-de Vries et al. (10)<br>(+) Fortin et al. (23, 24)<br>NS (physical component score); NS (mental component summary) Dziedzic et al. (5-7)
	SF-36	NS (physical health); (+) (mental health) Hoffmann et al. (11)
	SF-36PF	NS Friedberg et al. (40, 41)
	EQ-5D	NS Richards et al. (42)<br>NS Zimmermann et al. (8)<br>NS Kennedy et al. (21, 22)
	ADDQoL	NS Gabbay et al. (43)
	CRQ	NS Bischoff et al. (18)
	AQLQ	NS Mehuys et al. (44)
	WHO QoL-BREF	NS Browning et al. (16, 17)
	Juniper questionnaire	(+) McLean et al. **(45)**
	CAT	(+) Ferrone et al. (46)
	CCQ	(+) Ferrone et al. (46)
**Physical and social functioning**		
General functioning	Satisfaction with life scale; SAS-SR	(+) Banasiak et al. (47)[#]
Physical and social functioning	WOMAC	(+) (post-treatment); NS (6 months); NS (12 months) Broderick et al. (4)
General health and physical functioning	GHQ<br>Medical Outcome Study short form general health survey	(+) Chalder et al.(48)<br>(+) Chalder et al.(48)
Physical functioning	SF-36	NS Yardley et al. (31)
Perceived effect of diabetes on daily functioning	DFT	NS van Dijk-de Vries et al. (10)
Changes in physical and mental health status	SF-12	NS Murphy et al. (49-51)
	Warwick-Edinburgh Mental Well-being Scale	NEA Barley et al. (9)
Functional status	RDQ (modified)	NS Cherkin (52)[#]
Symptoms and functioning	Brief psychiatric rating scale	NS Bartels et al. (53)
Social functioning	Social Functioning scale from the SF-36	NS Von Korff et al.(54)

**Psychological functioning**		
Overall psychological functioning and wellbeing	CORE-OM	(+) Waite et al. (55)<br>NS Richards et al. (42)<br>(+) Williams et al. (56)
Self-esteem	RSCQ	(+) Waite et al. (55)
Depression score	PHQ	(+) Morgan et al. (57, 58)<br>(+) Watkins et al. (59)<br>NS Jaipakdee et al. (37)<br>NS Dziedzic et al. (5-7)
Anxiety score	GAD-7	(+) Watkins et al. (59)<br>NS Dziedzic et al. (5-7)
Cognitive symptoms	CSFBD	(+) Heitkemper et al. (36)
Level of distress	SCL-90R	(+) Heitkemper et al. (36)
	K10	(+) Browning et al. (16, 17)
Psychological/ psychosocial functioning	BDI-II	NS Friedberg et al. (40, 41)<br>(+) Waite et al. (55)<br>(+) Williams et al. (56)<br>(+) Banasiak et al. (47)[#]<br>(+) Watkins et al. (59)
Psychological distress	Mental Health Inventory	NS Von Korff et al.(54)
	Kessler Psychological Distress Scale	(+) Fortin et al. (23, 24)
Diabetes-related distress	PAID	(+) Gabbay et al. (60)[#]<br>NS Gabbay et al. (43)<br>(+) Sturt et al. (1)<br>NS van Dijk-de Vries et al. (10)
Level of anxiety and depression	HADS	NS Yardley et al. (31)<br>NS McGeoch et al. (29)<br>NS Moss-Morris et al. (32, 33)[#]<br>(+) (HADS anxiety); NS (HADS depression) Mitchell et al. (14, 15)<br>NEA Barley et al. (9)
	BDI	(+) Striegel-Moore et al. (34, 35)<br>(+) (post-treatment); NS (6 months); (+) (12 months) Broderick et al. (4)<br>NS Grilo et al. (61)
Coping	FQCI	NS Zimmermann et al. (8)
	CSQ	(+) Broderick et al. (4)
**Knowledge**		
Disease knowledge	Questionnaire (5-point scale)	(+) Cherkin et al. (52)
	Brief Diabetes Knowledge Test	(+) Mehuys et al. (62)
	Questionnaire (details not reported)	(+) Efraimsson et al. (27)<br>(+) McLean et al. **(45)**
	BCKQ	(+) Hill et al. (63)<br>(+) Mitchell et al. (14, 15)

		(+) Ferrone et al. (46)
	Knowledge of Asthma and Asthma Medicine questionnaire (updated version)	NS Mehuys et al. (44)
	KQ	NS Grilo et al. (61)
Self-management knowledge and behaviours	PIH-NL	NS van Dijk-de Vries et al. (10)
Self-management education	Health Education Impact Questionnaire (heiQ)	(+) Fortin et al. (23, 24)
**Behaviours**		
**Exercise**		
Level of exercise/ physical activity	Questionnaire (details not reported)	(+) Cherkin (52)
	IPAQ	NS Rosemann et al. (38)<br>NS Dziedzic et al. (5-7)
	PACE	NS Clark et al. (64, 65)
	PASE	NS Dziedzic et al. (5-7)
	Rapid Assessment of Physical Activity questionnaire	NS Eikelenboom et al. (25, 26)
Physical activity over the past 7 days (steps/ day)	Self-report diary card and pedometer recordings	NS Wood-Baker et al. (28)
**Smoking**		
Mean number of cigarettes smoked per day	Questionnaire (details not reported)	NS Meland et al. (12, 13)
Smoking behaviour	Questionnaire	NS Tiessen et al. (66, 67)[#]<br>(+) Efraimsson et al. (27)
Smoking status	Smoking status assessment questionnaire	NS Eikelenboom et al. (25, 26)
**Diet**		
Estimated daily grams of fat	The Block Fat Screener questionnaire (15 items)	NS Clark et al. (64, 65)
Dietary behaviour	Rapid Eating Assessment for Participants — short questionnaire	NS Eikelenboom et al. (25, 26)
**Self-management activities**		
Mean days per week engaging in 3 separate diet activities	Questionnaire (self-reported) *self-care items taken from Toobert et al. 2000.	(+) Doucette et al. (68)
Mean days per week engaging in 5 separate diabetes activities	Questionnaire (self-reported) *self-care items taken from Toobert et al. 2000.	(+) Doucette et al. (68)

Mean days per week engaging in 2 separate exercise activities	Questionnaire (self-reported) *self-care items taken from Toobert et al. 2000.	NS Doucette et al. (68)
Self-care activities	SDSCA	NS (general diet); (+) (specific diet); NS (physical exercise); NS (blood glucose monitoring); NS (foot care) Browning et al. (16, 17)
		NS (general diet); NS (specific diet); (+) (physical exercise); (+) (foot care); NS (smoking) Mehuys et al. (62)
		NS Gabbay et al. (43)
		(+) Clark et al. (64, 65)
General medical illness self-management	Stanford Chronic Disease self-efficacy scale	NS Bartels et al. (53)
Self-management	COPD-SMI	(+) (for all domains expect one) McGeoch et al. (29)
Psychiatric illness self-management	IMR scale	(+) Bartels et al. (53)
**Adherence**		
Adherence to medication (number of prescription refills; self-reported)	Questionnaire	(+); NS Mehuys et al. (62)
**Technique**		
Inhalation technique	Checklist	(+) Mehuys et al. (44)
**Disease specific**		
**Diabetes / cardiovascular conditions**		
Change in HbA1c / blood glucose	Clinical laboratory measurements	(+) Goudswaard et al. (69)<br>(+) Huang et al. (70, 71)<br>(+) Morgan et al. (57, 58)<br>(+) Adachi et al. (72)<br>(+) Olry de Labry Lima et al. (73)<br>(+) Mehuys et al. (62)<br>(+); NS Jaipakdee et al. (37)<br>NS Gabbay et al. (60)#<br>NS Sturt et al. (1)<br>NS Doucette et al. (68)<br>NS Farmer et al.(74-76)<br>NS Ismail et al. (77)<br>NS Browning et al. (16, 17)<br>NS Tiessen et al. (66, 67)#<br>NS van Dijk-de Vries et al. (10)<br>NS Gabbay et al. (43)<br>(-) Partapsingh et al. (78)#
Change in weight / BMI	Anthropometric measurements	(+) Goudswaard et al. (69)<br>NS Gabbay et al. (60)#

		NS Huang et al. (70, 71)<br>NS Morgan et al. (57, 58)#<br>NS Ismail et al. (77)<br>NS Adachi et al. (72)<br>NS Partapsingh et al. (78)<br>NS Olry de Labry Lima et al. (73)#<br>NS Browning et al. (16, 17)<br>NS Tiessen et al. (66, 67)#
Change in blood pressure	Blood pressure measurement	(+) Gabbay et al. (60)<br>(+) (SBP); NS (DBP) Huang et al. (70, 71)<br>(+) (SBP); NS (DBP) Browning et al. (16, 17)<br>(+) (SBP); NS (DBP) Gabbay et al. (43)<br>NS Meland et al. (12, 13)<br>NS Doucette et al. (68)<br>NS Morgan et al. (57, 58)#<br>NS Murphy et al. (49-51)<br>NS Ismail et al. (77)<br>NS Adachi et al. (72)<br>NS Partapsingh et al. (78)<br>NS Olry de Labry Lima et al. (73)<br>NS Tiessen et al. (66, 67)#
Change in lipids	Clinical laboratory test	NS Meland et al. (12, 13)<br>NS Gabbay et al. (60)#<br>NS Doucette et al. (68)<br>NS Huang et al. (70, 71)<br>NS Morgan et al. (57, 58)#<br>NS Murphy et al. (49-51)<br>NS Ismail et al. (77)<br>NS Adachi et al. (72)<br>NS Partapsingh et al. (78)<br>NS Olry de Labry Lima et al. (73)<br>NS Browning et al. (16, 17)<br>NS Tiessen et al. (66, 67)#<br>NS Gabbay et al. (43)
Chest pain	Modified Rose Angina Questionnaire	NEA Barley et al. (9)
**Chronic obstructive pulmonary disease**		
Pulmonary function	FEV1	NS Wood-Baker et al. (28)
		(+) Ferrone et al. (46)
Respiratory status	Diary (self-report)	NS Watson et al. (30)
Courses of antibiotics	COPD-SMI	NS McGeoch et al. (29)
	Diary (self-report)	(+) Wood-Baker et al. (28)<br>(+) Watson et al. (30)

Courses of oral steroids	COPD-SMI	NS McGeoch et al. (29)
	Diary (self-report)	(+) Wood-Baker et al. (28)<br>NS Watson et al. (30)
Symptom burden	CRQ-SR	(+) (6 weeks); NS (6 months) Mitchell et al. (14, 15)<br>NS Bischoff et al. (18)
Frequency and patients' management of exacerbations	TEXAS	NS Bischoff et al. (18)
	Diary (self-report)	(+) Ferrone et al. (46)
**Eating disorders**		
Frequency over the past 28 days of episodes of objective binge eating	EDE; EDE-Q	(+) Banasiak et al. (47)<br>NS Grilo et al. (61)
Frequency over the past 28 days of episodes of subjective binge eating	EDE; EDE-Q	(+) Banasiak et al. (47)[#]
Purging episodes score=primary purging behaviour (episodes of vomiting, or laxative, or diuretic or enema/suppository misuse).	EDE; EDE-Q	(+) Banasiak et al. (47)
Body mass index	Direct measurements of weight and height	NS Banasiak et al. (47)[#]<br>NS Grilo et al. (61)
Binge eating severity	The Gormally binge eating scale (16 items)	NEA Clark et al. (64, 65)[#]
Abstinence from binge eating	PHQ-ED (modified)	(+) Striegel-Moore et al. (34, 35)
**Asthma**		
Level of asthma control	ACT	NS Mehuys et al. (44)
PEF	PEFR (self-measured)	NS Mehuys et al. (44)<br>(+) McLean et al. **(45)**
Rescue medication use	Self-report diary	(+) Mehuys et al. (44)
Asthma symptoms	North of England asthma symptoms scale (10 items)	(+) Barbanel et al. (79)
	Self-report symptom scores	(+) McLean et al. **(45)**
Number of exacerbations	Self-report diary	NS Mehuys et al. (44)
Nocturnal awakenings over 14 days	Self-report diary	(+) Mehuys et al. (44)
**Migraine**		
Intensity of pain	Questionnaire (10 point scale)	NS Hoffmann et al. (11)
Number of days with headache	Kiel Headache Questionnaire	NS Hoffmann et al. (11)
**Back pain**		
Mean difference in pain intensity	Questionnaire (10 point scale)	NS Von Korff et al. (54)
Symptom relief	Questionnaire (10-point scale)	NS Cherkin (52)

**Dizziness**		
Change in spontaneous and provoked symptoms of dizziness	Vertigo Symptom Scale-Short Form	(+) Yardley et al. (31)
**Oral hygiene**		
Percentage of surfaces with plaque	Silness and Löe index	NS (RCT); (+) (cRCT) Clarkson et al.(2, 3)
Percentage sites bleeding	Silness and Löe index	NS Clarkson et al.(2, 3)
Timing, duration and method	Self-report (scale)	(+) Clarkson et al.(2, 3)
**Irritable bowel syndrome**		
Symptom severity	IBS-SSS	(+) Moss-Morris et al. (32, 33)#
**Chronic fatigue syndrome**		
Fatigue impact on functioning	FSS	(+) Friedberg et al. (40, 41)<br>(+) Chalder et al.(48)
**Depression**		
Depressive symptoms	HAM-D	(+) Watkins et al. (59)
	CES-D	NS Gabbay et al. (43)
Symptom load	PHQ-D	NS Zimmermann et al. (8)
**Osteoarthritis**		
Pain intensity	BPI	(+) (post-treatment); NS (6 months); (+) (12 months) Broderick et al. (4)
Peripheral joint pain intensity	OMERACT/OARSI responder criteria	(+) Dziedzic et al. (5-7)
Fatigue	BFI	(+) (post-treatment); NS (6 months); (+) (12 months) Broderick et al. (4)
Use of pain medication	Diary (self-report)	(+) (post-treatment); NS (6 months); (+) (12 months) Broderick et al. (4)
**Other**		
Satisfaction	Questionnaire (questionnaire with two subscales, "Information and General care")	(+) Cherkin (52)
Satisfaction with health	AIMS2-SF	(+) (post-treatment); NS (6 months); NS (12 months) Broderick et al. (4)
Depth of relationship; professional care and perceived time	Consultation satisfaction questionnaire	(+) Richards et al. (42)

Legend: (+): significant findings p<0.05; NS: non-significant findings; (-): negative findings; NEA: no evidence available; #: no p-value provided

Abbreviations: ACT: Asthma control test; ADDQoL: Audit of Diabetes Dependent Quality of Life; AIMS2-SF: Arthritis Impact Measurement Scales Short Form (78 items); AQLQ: Standardised Asthma Quality of Life Questionnaire; BCKQ: Bristol COPD Knowledge Questionnaire; BDI: Beck Depression Inventory (21 items); BDI-II: Beck Depression Inventory-II; BFI: Brief Fatigue Inventory; BMI: body mass index; BPI: Brief pain inventory; CAT: COPD assessment test; CCQ: Clinical COPD Questionnaire; CDMSES: Career Decision Making Self Efficacy Scale; CES-D: Centre for Epidemiologic Studies Depression scale; COPD-SMI: COPD-self management interview; CORE-OM: Clinical Outcomes in Routine Evaluation Outcome Measure; CRQ: Chronic respiratory questionnaire (20 items); CRQ-SR: Chronic Respiratory Questionnaire dyspnoea domain; CSFBD: Cognitive Scale for Functional Bowel Disorders; CSQ: Coping strategies questionnaire (42 items); DBP: diastolic blood pressure; DFT: Daily Functioning Thermometer; DMSES: Diabetes Management Self-efficacy Scale; DSES: Depression Self-Efficacy Scale (5 items); DTSQ: Diabetes Treatment Satisfaction Questionnaire; EDE: Eating Disorder Examination; EDE-Q: Eating Disorder Examination Questionnaire (EDE Q); EQ-5D: quality of life questionnaire (5 items); FEV1: Forced expiratory volume in one second; FQCI: Freiburg questionnaire of coping with illness; FSS: Fatigue Severity Scale; GAD-7: Generalized Anxiety Disorder-7; GHQ: General Health Questionnaire (12 items); GSE: general self-efficacy scale; GSES: General Self Efficacy Scale; HADS: Hospital Anxiety and Depression Scale (14 items); HAM-D: Depression rating scale (17 items); heiQ: Health Education Impact Questionnaire; HDL: high density lipoprotein; HRQOL: Health-related quality of life; IBSQOL: Irritable Bowel Syndrome quality of life questionnaire; IBS-SSS: Irritable Bowel Syndrome Severity Scoring System; IPAQ: Short form of the International Physical Activity Questionnaire; K10: Kessler 10 score; KQ: Knowledge Questionnaire (10 items); LDL: low density lipoprotein; PACE: The Physician-based Assessment and Counselling for Physical Activity (11 items); PAID: Problem Areas in Diabetes scale (20 items); PAM-13: Patient Activation Measure (13 items); PASE: Physical Activity Scale for the Elderly; PEF: Peak expiratory flow; PEFR: Peak expiratory flow rate; PEI: Patient Enablement Instrument; PHQ9: Patient Health Questionnaire-9; PHQ-D: Patient health questionnaire; PHQ-ED: Patient Health Questionnaire eating disorder module; PIH-NL: Partners in Health scale (12 items); PRAISE: Pulmonary Rehabilitation Adapted Index of Self-Efficacy; RDQ: Roland Disability Questionnaire (23 items); RSCQ: Robson Self-concept Questionnaire (30 items); SAS-SR: Overall adjustment score of the modified social adjustment scale; SBP: systolic blood pressure; SCL-90R: Symptoms checklist (90 items); SDSCA: Summary of Diabetes Self-Care Activities; SF-12: Short form survey (12 items); SF-36: Short form survey (36 items); SF-36PF: Short Form-36 Physical Function subscale; SGRQ: St George's Respiratory Questionnaire (50 items); TC: total cholesterol; TEXAS: Nijmegen telephonic exacerbation assessment system; TG: triglycerides; WHO QoL-BREF: World Health Organisation Quality of Life-BREF; WOMAC: Western Ontario and McMaster Universities Osteoarthritis Index (24 items); WSAS: Work and Social Adjustment Scale (5 items)

References

1. Sturt J, Whitlock S, Fox C, Hearnshaw H, Farmer A, Wakelin M, et al. Effects of the Diabetes Manual 1:1 structured education in primary care. Diabetic medicine : a journal of the British Diabetic Association. 2008;25(6):722-31.
2. Blinkhorn A. Influencing patients' oral hygiene behaviour. Evidence-based dentistry. 2010;11(2):41.
3. Clarkson J, Young L, Ramsay C, Bonner B, Bonetti D. How to influence patient oral hygiene behavior effectively. Journal of dental research. 2009;88(10):933-7.
4. Broderick J, Keefe F, Bruckenthal P, Junghaenel D, Schneider S, Schwartz J, et al. Nurse practitioners can effectively deliver pain coping skills training to osteoarthritis patients with chronic pain: A randomized, controlled trial. Pain. 2014;155(9):1743-54.
5. Porcheret M, Grime J, Main C, Dziedzic K. Developing a model osteoarthritis consultation: a Delphi consensus exercise. BMC musculoskeletal disorders. 2013;14:25.

6. Dziedzic KS, Healey EL, Porcheret M, Ong BN, Main CJ, Jordan KP, et al. Implementing the NICE osteoarthritis guidelines: a mixed methods study and cluster randomised trial of a model osteoarthritis consultation in primary care--the Management of OsteoArthritis In Consultations (MOSAICS) study protocol. Implement Sci. 2014;9:95.
7. Dziedzic KS, Healey EL, Porcheret M, Afolabi EK, Lewis M, Morden A, et al. Implementing core NICE guidelines for osteoarthritis in primary care with a model consultation (MOSAICS): a cluster randomised controlled trial. Osteoarthritis and cartilage. 2018;26(1):43-53.
8. Zimmermann T, Puschmann E, van den Bussche H, Wiese B, Ernst A, Porzelt S, et al. Collaborative nurse-led self-management support for primary care patients with anxiety, depressive or somatic symptoms: Cluster-randomised controlled trial (findings of the SMADS study). International journal of nursing studies. 2016;63:101-11.
9. Barley E, Walters P, Haddad M, Phillips R, Achilla E, McCrone P, et al. The UPBEAT nurse-delivered personalized care intervention for people with coronary heart disease who report current chest pain and depression: a randomised controlled pilot study. PloS one. 2014;9(6):e98704.
10. van Dijk-de Vries A, van Bokhoven M, Winkens B, Terluin B, Knottnerus J, van der Weijden T, et al. Lessons learnt from a cluster-randomised trial evaluating the effectiveness of Self-Management Support (SMS) delivered by practice nurses in routine diabetes care. BMJ open. 2015;5(6):e007014.
11. Hoffmann W, Herzog B, Muhlig S, Kayser H, Fabian R, Thomsen M, et al. Pharmaceutical care for migraine and headache patients: a community-based, randomized intervention. The Annals of pharmacotherapy. 2008;42(12):1804-13.
12. Meland E, Laerum E, Ulvik R. Effectiveness of two preventive interventions for coronary heart disease in primary care. Scandinavian journal of primary health care. 1997;15(1):57-64.
13. Meland E, Maeland J, Laerum E. The importance of self-efficacy in cardiovascular risk factor change. Scandinavian journal of public health. 1999;27(1):11-7.
14. Mitchell K, Johnson-Warrington V, Apps L, Bankart J, Sewell L, Williams J, et al. A self-management programme for COPD: a randomised controlled trial. The European respiratory journal. 2014;44(6):1538-47.
15. Dritsaki M, Johnson-Warrington V, Mitchell K, Singh S, Rees K. An economic evaluation of a self-management programme of activity, coping and education for patients with chronic obstructive pulmonary disease. Chron Respir Dis. 2016;13(1):48-56.
16. Browning C, Chapman A, Yang H, Liu S, Zhang T, Enticott J, et al. Management of type 2 diabetes in China: the Happy Life Club, a pragmatic cluster randomised controlled trial using health coaches. BMJ open. 2016;6(3):e009319.
17. Browning C, Yang H, Zhang T, Chapman A, Liu S, Enticott J, et al. Implementing a Chronic Disease Self-Management Program into China: The Happy Life Club™. Frontiers in public health. 2014;2:181.
18. Bischoff E, Akkermans R, Bourbeau J, van Weel C, Vercoulen J, Schermer T. Comprehensive self management and routine monitoring in chronic obstructive pulmonary disease patients in general practice: randomised controlled trial. British Medical Journal. 2012;345.
19. Smit A, Tiemens B, Ormel J, Kluiter H, Jenner J, van de Meer K, et al. Enhanced treatment for depression in primary care: first year results on compliance, self-efficacy, the use of antidepressants and contacts with the primary care physician. Primary Care & Community Psychiatry. 2005;10(2):39-49.
20. Stant A, TenVergert E, Kluiter H, Conradi H, Smit A, Ormel J. Cost-effectiveness of a psychoeducational relapse prevention program for depression in primary care. The journal of mental health policy and economics. 2009;12(4):195-204.
21. Kennedy A, Bower P, Reeves D, Blakeman T, Bowen R, Chew-Graham C, et al. Implementation of self management support for long term conditions in routine primary care settings: cluster randomised controlled trial. BMJ : British Medical Journal. 2013;346:f2882.

22. Kennedy A, Chew-Graham C, Blakeman T, Bowen A, Gardner C, Protheroe J, et al. Delivering the WISE (Whole Systems Informing Self-Management Engagement) training package in primary care: learning from formative evaluation. Implement Sci. 2010;5:7-.
23. Fortin M, Chouinard M-C, Dubois M-F, Bélanger M, Almirall J, Bouhali T, et al. Integration of chronic disease prevention and management services into primary care: a pragmatic randomized controlled trial (PR1MaC). CMAJ Open. 2016;4(4):E588-E98.
24. Contant É, Loignon C, Bouhali T, Almirall J, Fortin M. A multidisciplinary self-management intervention among patients with multimorbidity and the impact of socioeconomic factors on results. BMC Family Practice. 2019;20(1):53.
25. Eikelenboom N, van Lieshout J, Jacobs A, Verhulst F, Lacroix J, van Halteren A, et al. Effectiveness of personalised support for self-management in primary care: a cluster randomised controlled trial. British Journal of General Practice. 2016;66(646):e354-61.
26. Eikelenboom N, van Lieshout J, Wensing M, Smeele I, Jacobs A. Implementation of personalized self-management support using the self-management screening questionnaire SeMaS; a study protocol for a cluster randomized trial. Trials. 2013;14:336.
27. Efraimsson E, Hillervik C, Ehrenberg A. Effects of COPD self-care management education at a nurse-led primary health care clinic. Scandinavian journal of caring sciences. 2008;22(2):178-85.
28. Wood-Baker R, McGlone S, Venn A, Walters E. Written action plans in chronic obstructive pulmonary disease increase appropriate treatment for acute exacerbations. Respirology. 2006;11(5):619-26.
29. McGeoch G, Willsman K, Dowson C, Town G, Frampton C, F. M, et al. Self-management plans in the primary care of patients with chronic obstructive pulmonary disease. Respirology. 2006;11(5):611-8.
30. Watson PB, Town GI, Holbrook N, Dwan C, Toop LJ, Drennan CJ. Evaluation of a self-management plan for chronic obstructive pulmonary disease. The European respiratory journal. 1997;10(6):1267-71.
31. Yardley L, Donovan-Hall M, Smith H, Walsh B, Mullee M, Bronstein A. Effectiveness of primary care-based vestibular rehabilitation for chronic dizziness. Annals of internal medicine. 2004;141(8):598-605.
32. Moss-Morris R, McAlpine L, Didsbury L, Spence M. A randomized controlled trial of a cognitive behavioural therapy-based self-management intervention for irritable bowel syndrome in primary care. Psychological medicine. 2010;40(1):85-94.
33. Chilcot J, Moss-Morris R. Changes in illness-related cognitions rather than distress mediate improvements in irritable bowel syndrome (IBS) symptoms and disability following a brief cognitive behavioural therapy intervention. Behaviour research and therapy. 2013;51(10):690-5.
34. Striegel-Moore R, Wilson T, DeBar L, Perrin N, Lynch F, Rosselli F, et al. Cognitive Behavioral Guided Self-Help for the Treatment of Recurrent Binge Eating. Journal of consulting and clinical psychology. 2010;78(3):312-21.
35. DeBar L, Striegel-Moore R, Wilson T, Perrin N, Yarborough B, Dickerson J, et al. Guided Self-Help Treatment for Recurrent Binge Eating: Replication and Extension. Psychiatric Services. 2011;62(4):367-73.
36. Heitkemper M, Jarrett M, Levy R, Cain K, Burr R, Feld A, et al. Self-management for women with irritable bowel syndrome. Clinical Gastroenterology and Hepatology. 2004;2(7):585-96.
37. Jaipakdee J, Jiamjarasrangsi W, Lohsoonthorn V, Lertmaharit S. Effectiveness of a self-management support program for Thais with type 2 diabetes: Evaluation according to the RE-AIM framework. Nursing & Health Sciences. 2015;17(3):362-9.
38. Rosemann T, Joos S, Laux G, Gensichen J, Szecsenyi J. Case management of arthritis patients in primary care: a cluster-randomized controlled trial. Arthritis and rheumatism. 2007;57(8):1390-7.
39. Freund K, Lous J. The effect of preventive consultations on young adults with psychosocial problems: a randomized trial. Health education research. 2012;27(5):927-45.

40. Friedberg F, Napoli A, Coronel J, Adamowicz J, Seva V, Caikauskaite I, et al. Chronic fatigue self-management in primary care: a randomized trial. Psychosomatic medicine. 2013;75(7):650-7.
41. Meng H, Friedberg F, Castora-Binkley M. Cost-effectiveness of chronic fatigue self-management versus usual care: a pilot randomized controlled trial. BMC family practice. 2014;15:184.
42. Richards A, Barkham M, Cahill J, Richards D, Williams C, Heywood P. PHASE: a randomised, controlled trial of supervised self-help cognitive behavioural therapy in primary care. The British journal of general practice : the journal of the Royal College of General Practitioners. 2003;53(495):764-70.
43. Gabbay R, Anel-Tiangco R, Dellasega C, Mauger D, Adelman A, Van Horn D. Diabetes nurse case management and motivational interviewing for change (DYNAMIC): Results of a 2-year randomized controlled pragmatic trial. Journal of Diabetes. 2013;5(3):349-57.
44. Mehuys E, Van Bortel L, De Bolle L, Van Tongelen I, Annemans L, Remon J, et al. Effectiveness of pharmacist intervention for asthma control improvement. The European respiratory journal. 2008;31(4):790-9.
45. McLean W, Gillis J, Waller R. The BC Community Pharmacy Asthma Study: A study of clinical, economic and holistic outcomes influenced by an asthma care protocol provided by specially trained community pharmacists in British Columbia. Canadian respiratory journal. 2003;10(4):195-202.
46. Ferrone M, Masciantonio MG, Malus N, Stitt L, O'Callahan T, Roberts Z, et al. The impact of integrated disease management in high-risk COPD patients in primary care. npj Primary Care Respiratory Medicine. 2019;29(1):8.
47. Banasiak S, Paxton S, Hay P. Guided self-help for bulimia nervosa in primary care: a randomized controlled trial. Psychological medicine. 2005;35(9):1283-94.
48. Chalder T, Wallace P, Wessely S. Self-help treatment of chronic fatigue in the community: A randomized controlled trial. British Journal of Health Psychology. 1997;2(3):189-97.
49. Murphy A, Cupples M, Smith S, Byrne M, Byrne M, Newell J. Effect of tailored practice and patient care plans on secondary prevention of heart disease in general practice: cluster randomised controlled trial. BMJ (Clinical research ed). 2009;339:b4220.
50. Murphy A, Cupples M, Smith S, Byrne M, Leathem C, Byrne M. The SPHERE Study. Secondary prevention of heart disease in general practice: protocol of a randomised controlled trial of tailored practice and patient care plans with parallel qualitative, economic and policy analyses. Current controlled trials in cardiovascular medicine. 2005;6:11.
51. Gillespie P, O'Shea E, Smith S, Cupples M, Murphy A. A comparison of medical records and patient questionnaires as sources for the estimation of costs within research studies and the implications for economic evaluation. Family practice. 2016.
52. Cherkin D, Deyo R, Street J, Hunt M, Barlow W. Pitfalls of patient education. Limited success of a program for back pain in primary care. Spine. 1996;21(3):345-55.
53. Bartels S, Pratt S, Mueser K, Naslund J, Wolfe R, Santos M, et al. Integrated IMR for psychiatric and general medical illness for adults aged 50 or older with serious mental illness. Psychiatric services (Washington, DC). 2014;65(3):330-7.
54. Von Korff M, Balderson B, Saunders K, Miglioretti D, Lin E, Berry S, et al. A trial of an activating intervention for chronic back pain in primary care and physical therapy settings. Pain. 2005;113(3):323-30.
55. Waite P, McManus F, Shafran R. Cognitive behaviour therapy for low self-esteem: a preliminary randomized controlled trial in a primary care setting. Journal of behavior therapy and experimental psychiatry. 2012;43(4):1049-57.
56. Williams C, Wilson P, Morrison J, McMahon A, Walker A, Allan L, et al. Guided self-help cognitive behavioural therapy for depression in primary care: a randomised controlled trial. PloS one. 2013;8(1):e52735.
57. Morgan M, Dunbar J, Reddy P, Coates M, Leahy R. The TrueBlue study: is practice nurse-led collaborative care effective in the management of depression for patients with heart disease or diabetes? BMC family practice. 2009;10:46.

58. Morgan M, Coates M, Dunbar J, Reddy P, Schlicht K, Fuller J. The TrueBlue model of collaborative care using practice nurses as case managers for depression alongside diabetes or heart disease: a randomised trial. BMJ open. 2013;3(1).
59. Watkins E, Taylor R, Byng R, Baeyens C, Read R, Pearson K, et al. Guided self-help concreteness training as an intervention for major depression in primary care: a Phase II randomized controlled trial. Psychological medicine. 2012;42(7):1359-71.
60. Gabbay R, Lendel I, Saleem T, Shaeffer G, Adelman A, Mauger D, et al. Nurse case management improves blood pressure, emotional distress and diabetes complication screening. Diabetes research and clinical practice. 2006;71(1):28-35.
61. Grilo C, White M, Gueorguieva R, Barnes R, Masheb R. Self-help for binge eating disorder in primary care: a randomized controlled trial with ethnically and racially diverse obese patients. Behaviour research and therapy. 2013;51(12):855-61.
62. Mehuys E, Van Bortel L, De Bolle L, Van Tongelen I, Annemans L, Remon J, et al. Effectiveness of a community pharmacist intervention in diabetes care: a randomized controlled trial. Journal of clinical pharmacy and therapeutics. 2011;36(5):602-13.
63. Hill K, Mangovski-Alzamora S, Blouin M, Guyatt G, Heels-Ansdell D, Bragaglia P, et al. Disease-specific education in the primary care setting increases the knowledge of people with chronic obstructive pulmonary disease: A randomized controlled trial. Patient education and counseling. 2010;81(1):14-8.
64. Clark M, Hampson S. Implementing a psychological intervention to improve lifestyle self-management in patients with Type 2 diabetes. Patient education and counseling. 2001;42(3):247-56.
65. Clark M, Hampson S, Avery L, Simpson R. Effects of a tailored lifestyle self-management intervention in patients with Type 2 diabetes. British Journal of Health Psychology. 2004;9:365-79.
66. Tiessen A, Smit A, Broer J, Groenier K, van der Meer K. Randomized controlled trial on cardiovascular risk management by practice nurses supported by self-monitoring in primary care. BMC family practice. 2012;13:90.
67. Tiessen A, Vermeulen K, Broer J, Smit A, van der Meer K. Cost-effectiveness of cardiovascular risk management by practice nurses in primary care. BMC public health. 2013;13:148.
68. Doucette W, Witry M, Farris K, McDonough R. Community pharmacist-provided extended diabetes care. The Annals of pharmacotherapy. 2009;43(5):882-9.
69. Goudswaard A, Stolk R, Zuithoff N, de Valk H, Rutten G. Long-term effects of self-management education for patients with Type 2 diabetes taking maximal oral hypoglycaemic therapy: a randomized trial in primary care. Diabetic medicine : a journal of the British Diabetic Association. 2004;21(5):491-6.
70. Huang M, Hsu C, Wang H, Shin S. Prospective randomized controlled trial to evaluate effectiveness of registered dietitian-led diabetes management on glycemic and diet control in a primary care setting in Taiwan. Diabetes care. 2010;33(2):233-9.
71. Hsu C, Tai T. Long-term glycemic control by a diabetes case-management program and the challenges of diabetes care in Taiwan. Diabetes research and clinical practice. 2014;106(S2):S328-S32.
72. Adachi M, Yamaoka K, Watanabe M, Nishikawa M, Kobayashi I, Hida E, et al. Effects of lifestyle education program for type 2 diabetes patients in clinics: a cluster randomized controlled trial. BMC public health. 2013;13:467.
73. Olry de Labry Lima A, Bermudez Tamayo C, Pastor Moreno G, Bolivar Munoz J, Ruiz Perez I, Johri M, et al. Effectiveness of an intervention to improve diabetes self-management on clinical outcomes in patients with low educational level. Gaceta sanitaria / SESPAS. 2016.
74. Farmer A, Wade A, Goyder E, Yudkin P, French D, Craven A, et al. Impact of self monitoring of blood glucose in the management of patients with non-insulin treated diabetes: open parallel group randomised trial. BMJ (Clinical research ed). 2007;335(7611):132.
75. Simon J, Gray A, Clarke P, Wade A, Neil A, Farmer A. Cost effectiveness of self monitoring of blood glucose in patients with non-insulin treated type 2 diabetes: economic evaluation of data from the DiGEM trial. BMJ (Clinical research ed). 2008;336(7654):1177-80.

76. Farmer A, Wade A, French D, Simon J, Yudkin P, Gray A, et al. Blood glucose self-monitoring in type 2 diabetes: a randomised controlled trial. Health technology assessment (Winchester, England). 2009;13(15):iii-iv, ix-xi, 1-50.
77. Ismail M, Teng C, Omar M, Ho B, Kusiar Z, Hasim R. Usage of glucometer is associated with improved glycaemic control in type 2 diabetes mellitus patients in Malaysian public primary care clinics: an open-label, randomised controlled trial. Singapore Med J. 2013;54(7):391-5.
78. Partapsingh V, Maharaj R, Rawlins J. Applying the Stages of Change model to Type 2 diabetes care in Trinidad: A randomised trial. Journal of Negative Results in BioMedicine. 2011;10(1).
79. Barbanel D, Eldridge S, Griffiths C. Can a self-management programme delivered by a community pharmacist improve asthma control? A randomised trial. Thorax. 2003;58(10):851-4.
