# Supplementary material for: Helping patients help themselves: A systematic review of self-management support strategies in primary health care practice
Source: PLoS One. 2019 Aug 1;14(8):e0220116. doi: 10.1371/journal.pone.0220116 (PMC6675068; doi:10.1371/journal.pone.0220116)
Supplement: S5 Table — (PDF) [file pone.0220116.s006.pdf]

S5 Table. Mapping of intervention components

	Transfer of information	Self-treatment through use of an action plan	Active stimulation of symptom monitoring	Stress or psychological management	Enhancing problem solving/ decision-making	Resource utilization	Enhancing physical activity	Enhancing dietary intake	Enhancing smoking cessation	Enhancing medication adherence
Adachi et al. (1)	✓	×	×	✓	×	×	✓	✓	×	×
Banasiak et al. (2)	✓	×	×	×	✓	×	×	×	×	×
Barbanel et al. (3)	✓	✓	✓	×	✓	×	×	×	✓	✓
Barley et al. (4)	✓	✓	×	×	×	✓	×	×	×	×
Bartels et al. (5)	✓	×	×	✓	×	×	×	×	×	✓
Bischoff et al. (6)	✓	✓	×	✓	×	×	×	×	×	✓
Broderick et al. (7)	✓	×	✓	✓	✓	×	×	×	×	×
Browning et al. (8, 9)	✓	×	×	×	✓	×	×	×	×	×
Chalder et al. (10)	✓	×	✓	×	✓	×	×	×	×	×
Cherkin et al. (11)	✓	×	✓	×	×	×	✓	×	×	×
Clark et al. (12, 13)	✓	×	×	×	✓	×	✓	✓	×	×
Clarkson et al.(14, 15)	✓	✓	×	×	×	×	×	×	×	×
Doucette et al. (16)	✓	✓	×	×	×	×	×	×	×	✓
Dziedzic et al. (17-19)	✓	✓	×	×	×	✓	✓	✓	×	×
Efraimsson et al. (20)	✓	✓	×	×	×	✓	✓	✓	✓	✓
Eikelenboom et al. (21, 22)	✓	✓	✓	×	✓	×	×	×	×	×
Farmer et al. (23-25)	✓	×	✓	×	✓	×	✓	✓	×	✓
Ferrone et al. (26)	✓	✓	×	✓	✓	×	✓	✓	✓	✓
Fortin et al. (27, 28)	✓	×	×	×	×	×	✓	✓	✓	×
Freund et al. (29)	✓	✓	✓	×	✓	✓	×	×	×	×

Friedberg et al. (30, 31)	✓	✓	✓	✓	✓	×	✓	×	×	×
Gabbay et al. (32)	✓	✓	×	×	×	✓	×	×	×	×
Gabbay et al. (33)	✓	×	×	×	×	✓	×	×	×	✓
Goudswaard et al. (34)	✓	×	✓	×	×	×	✓	✓	×	✓
Grilo et al.(35)	✓	×	✓	✓	×	×	×	✓	×	×
Heitkemper et al. (36)	✓	✓	✓	✓	✓	×	×	✓	×	×
Hill et al. (37)	✓	×	×	×	×	×	✓	×	✓	✓
Hoffmann et al. (38)	✓	✓	×	×	×	×	×	×	×	×
Huang et al. (39, 40)	✓	✓	✓	×	×	×	✓	✓	×	✓
Ismail et al. (41)	✓	✓	✓	×	✓	×	×	✓	×	✓
Jaipakdee et al. (42)	✓	×	✓	✓	✓	×	✓	✓	×	✓
Kennedy et al. (43, 44)	✓	×	×	✓	×	✓	×	×	×	×
McGeoch et al. (45)	✓	✓	✓	×	×	×	×	×	×	×
McLean et al. (46)	✓	✓	✓	×	✓	×	×	×	×	✓
Mehuys et al. (47)	✓	×	×	×	×	✓	×	×	✓	✓
Mehuys et al. (48)	✓	×	×	×	×	✓	✓	✓	✓	✓
Meland et al.(49, 50)	✓	×	✓	✓	✓	×	✓	✓	✓	×
Mitchell et al. (51, 52)	✓	×	×	✓	×	×	✓	×	×	×
Morgan et al. (53, 54)	✓	✓	×	×	✓	×	×	×	×	×
Moss-Morris et al. (55, 56)	✓	×	✓	✓	×	×	✓	✓	×	×
Murphy et al. (57-59)	✓	✓	×	✓	×	✓	✓	✓	✓	✓
Olry de Labry Lima et al. (60)	✓	×	×	×	×	✓	✓	✓	×	×
Partapsingh et al. (61)	✓	×	×	×	×	×	✓	✓	×	✓
Richards et al. (62)	✓	×	×	✓	×	×	×	×	×	×
Rosemann et al. (63)	✓	×	×	✓	×	✓	✓	×	×	×
Smit et al. (64, 65)	✓	✓	✓	✓	×	×	×	×	×	×
Striegel-Moore et al.(66, 67)	✓	×	✓	✓	✓	✓	×	✓	×	×
Sturt et al. (68)	✓	×	✓	✓	✓	×	✓	✓	✓	✓
Tiessen et al. (69, 70)	✓	×	✓	×	×	×	✓	✓	✓	×
van Dijk-de Vries et al. (71)	✓	✓	✓	✓	✓	×	×	×	×	×

Von Korff et al. (72)	✓	✓	×	×	✓	×	✓	×	×	×
Waite et al. (73)	✓	×	×	✓	×	✓	×	×	×	×
Watkins et al. (74)	✓	×	×	✓	×	✓	×	×	×	×
Watson et al. (75)	✓	✓	✓	×	×	×	✓	✓	✓	✓
Williams et al. (76)	✓	×	×	✓	✓	×	×	×	×	×
Wood-Baker et al. (77)	✓	✓	×	✓	×	✓	✓	✓	✓	✓
Yardley et al. (78)	✓	✓	✓	×	✓	×	✓	×	×	×
Zimmermann et al. (79)	✓	×	×	✓	✓	×	×	×	×	×
**Total**	57	26	25	25	24	13	27	24	13	21

Legend: (✓) component present; (×): component absent/ unclear/ not specified

References

1. Adachi M, Yamaoka K, Watanabe M, Nishikawa M, Kobayashi I, Hida E, et al. Effects of lifestyle education program for type 2 diabetes patients in clinics: a cluster randomized controlled trial. BMC public health. 2013;13:467.
2. Banasiak S, Paxton S, Hay P. Guided self-help for bulimia nervosa in primary care: a randomized controlled trial. Psychological medicine. 2005;35(9):1283-94.
3. Barbanel D, Eldridge S, Griffiths C. Can a self-management programme delivered by a community pharmacist improve asthma control? A randomised trial. Thorax. 2003;58(10):851-4.
4. Barley E, Walters P, Haddad M, Phillips R, Achilla E, McCrone P, et al. The UPBEAT nurse-delivered personalized care intervention for people with coronary heart disease who report current chest pain and depression: a randomised controlled pilot study. PloS one. 2014;9(6):e98704.
5. Bartels S, Pratt S, Mueser K, Naslund J, Wolfe R, Santos M, et al. Integrated IMR for psychiatric and general medical illness for adults aged 50 or older with serious mental illness. Psychiatric services (Washington, DC). 2014;65(3):330-7.
6. Bischoff E, Akkermans R, Bourbeau J, van Weel C, Vercoulen J, Schermer T. Comprehensive self management and routine monitoring in chronic obstructive pulmonary disease patients in general practice: randomised controlled trial. BMJ : British Medical Journal. 2012;345.
7. Broderick J, Keefe F, Bruckenthal P, Junghaenel D, Schneider S, Schwartz J, et al. Nurse practitioners can effectively deliver pain coping skills training to osteoarthritis patients with chronic pain: A randomized, controlled trial. Pain. 2014;155(9):1743-54.
8. Browning C, Chapman A, Yang H, Liu S, Zhang T, Enticott J, et al. Management of type 2 diabetes in China: the Happy Life Club, a pragmatic cluster randomised controlled trial using health coaches. BMJ open. 2016;6(3):e009319.
9. Browning C, Yang H, Zhang T, Chapman A, Liu S, Enticott J, et al. Implementing a Chronic Disease Self-Management Program into China: The Happy Life Club™. Frontiers in public health. 2014;2:181.
10. Chalder T, Wallace P, Wessely S. Self-help treatment of chronic fatigue in the community: A randomized controlled trial. British Journal of Health Psychology. 1997;2(3):189-97.
11. Cherkin D, Deyo R, Street J, Hunt M, Barlow W. Pitfalls of patient education. Limited success of a program for back pain in primary care. Spine. 1996;21(3):345-55.
12. Clark M, Hampson S. Implementing a psychological intervention to improve lifestyle self-management in patients with Type 2 diabetes. Patient education and counseling. 2001;42(3):247-56.
13. Clark M, Hampson S, Avery L, Simpson R. Effects of a tailored lifestyle self-management intervention in patients with Type 2 diabetes. British Journal of Health Psychology. 2004;9:365-79.
14. Blinkhorn A. Influencing patients' oral hygiene behaviour. Evidence-based dentistry. 2010;11(2):41.
15. Clarkson J, Young L, Ramsay C, Bonner B, Bonetti D. How to influence patient oral hygiene behavior effectively. Journal of dental research. 2009;88(10):933-7.
16. Doucette W, Witry M, Farris K, McDonough R. Community pharmacist-provided extended diabetes care. The Annals of pharmacotherapy. 2009;43(5):882-9.
17. Porcheret M, Grime J, Main C, Dziedzic K. Developing a model osteoarthritis consultation: a Delphi consensus exercise. BMC musculoskeletal disorders. 2013;14:25.
18. Dziedzic KS, Healey EL, Porcheret M, Ong BN, Main CJ, Jordan KP, et al. Implementing the NICE osteoarthritis guidelines: a mixed methods study and cluster randomised trial of a model osteoarthritis consultation in primary care--the Management of OsteoArthritis In Consultations (MOSAICS) study protocol. Implement Sci. 2014;9:95.
19. Dziedzic KS, Healey EL, Porcheret M, Afolabi EK, Lewis M, Morden A, et al. Implementing core NICE guidelines for osteoarthritis in primary care with a model consultation (MOSAICS): a cluster randomised controlled trial. Osteoarthritis and cartilage. 2018;26(1):43-53.
20. Efraimsson E, Hillervik C, Ehrenberg A. Effects of COPD self-care management education at a nurse-led primary health care clinic. Scandinavian journal of caring sciences. 2008;22(2):178-85.

21. Eikelenboom N, van Lieshout J, Jacobs A, Verhulst F, Lacroix J, van Halteren A, et al. Effectiveness of personalised support for self-management in primary care: a cluster randomised controlled trial. British Journal of General Practice. 2016;66(646):e354-61.
22. Eikelenboom N, van Lieshout J, Wensing M, Smeele I, Jacobs A. Implementation of personalized self-management support using the self-management screening questionnaire SeMaS; a study protocol for a cluster randomized trial. Trials. 2013;14:336.
23. Farmer A, Wade A, Goyder E, Yudkin P, French D, Craven A, et al. Impact of self monitoring of blood glucose in the management of patients with non-insulin treated diabetes: open parallel group randomised trial. BMJ (Clinical research ed). 2007;335(7611):132.
24. Simon J, Gray A, Clarke P, Wade A, Neil A, Farmer A. Cost effectiveness of self monitoring of blood glucose in patients with non-insulin treated type 2 diabetes: economic evaluation of data from the DiGEM trial. BMJ (Clinical research ed). 2008;336(7654):1177-80.
25. Farmer A, Wade A, French D, Simon J, Yudkin P, Gray A, et al. Blood glucose self-monitoring in type 2 diabetes: a randomised controlled trial. Health technology assessment (Winchester, England). 2009;13(15):iii-iv, ix-xi, 1-50.
26. Ferrone M, Masciantonio MG, Malus N, Stitt L, O'Callahan T, Roberts Z, et al. The impact of integrated disease management in high-risk COPD patients in primary care. npj Primary Care Respiratory Medicine. 2019;29(1):8.
27. Fortin M, Chouinard M-C, Dubois M-F, Bélanger M, Almirall J, Bouhali T, et al. Integration of chronic disease prevention and management services into primary care: a pragmatic randomized controlled trial (PR1MaC). CMAJ Open. 2016;4(4):E588-E98.
28. Contant É, Loignon C, Bouhali T, Almirall J, Fortin M. A multidisciplinary self-management intervention among patients with multimorbidity and the impact of socioeconomic factors on results. BMC Family Practice. 2019;20(1):53.
29. Freund K, Lous J. The effect of preventive consultations on young adults with psychosocial problems: a randomized trial. Health education research. 2012;27(5):927-45.
30. Friedberg F, Napoli A, Coronel J, Adamowicz J, Seva V, Caikauskaite I, et al. Chronic fatigue self-management in primary care: a randomized trial. Psychosomatic medicine. 2013;75(7):650-7.
31. Meng H, Friedberg F, Castora-Binkley M. Cost-effectiveness of chronic fatigue self-management versus usual care: a pilot randomized controlled trial. BMC family practice. 2014;15:184.
32. Gabbay R, Lendel I, Saleem T, Shaeffer G, Adelman A, Mauger D, et al. Nurse case management improves blood pressure, emotional distress and diabetes complication screening. Diabetes research and clinical practice. 2006;71(1):28-35.
33. Gabbay R, Anel-Tiangco R, Dellasega C, Mauger D, Adelman A, Van Horn D. Diabetes nurse case management and motivational interviewing for change (DYNAMIC): Results of a 2-year randomized controlled pragmatic trial. Journal of Diabetes. 2013;5(3):349-57.
34. Goudswaard A, Stolk R, Zuithoff N, de Valk H, Rutten G. Long-term effects of self-management education for patients with Type 2 diabetes taking maximal oral hypoglycaemic therapy: a randomized trial in primary care. Diabetic medicine : a journal of the British Diabetic Association. 2004;21(5):491-6.
35. Grilo C, White M, Gueorguieva R, Barnes R, Masheb R. Self-help for binge eating disorder in primary care: a randomized controlled trial with ethnically and racially diverse obese patients. Behaviour research and therapy. 2013;51(12):855-61.
36. Heitkemper M, Jarrett M, Levy R, Cain K, Burr R, Feld A, et al. Self-management for women with irritable bowel syndrome. Clinical Gastroenterology and Hepatology. 2004;2(7):585-96.
37. Hill K, Mangovski-Alzamora S, Blouin M, Guyatt G, Heels-Ansdell D, Bragaglia P, et al. Disease-specific education in the primary care setting increases the knowledge of people with chronic obstructive pulmonary disease: A randomized controlled trial. Patient education and counseling. 2010;81(1):14-8.

38. Hoffmann W, Herzog B, Muhlig S, Kayser H, Fabian R, Thomsen M, et al. Pharmaceutical care for migraine and headache patients: a community-based, randomized intervention. The Annals of pharmacotherapy. 2008;42(12):1804-13.
39. Huang M, Hsu C, Wang H, Shin S. Prospective randomized controlled trial to evaluate effectiveness of registered dietitian-led diabetes management on glycemic and diet control in a primary care setting in Taiwan. Diabetes care. 2010;33(2):233-9.
40. Hsu C, Tai T. Long-term glycemic control by a diabetes case-management program and the challenges of diabetes care in Taiwan. Diabetes research and clinical practice. 2014;106(S2):S328-S32.
41. Ismail M, Teng C, Omar M, Ho B, Kusiar Z, Hasim R. Usage of glucometer is associated with improved glycaemic control in type 2 diabetes mellitus patients in Malaysian public primary care clinics: an open-label, randomised controlled trial. Singapore Med J. 2013;54(7):391-5.
42. Jaipakdee J, Jiamjarasrangsi W, Lohsoonthorn V, Lertmaharit S. Effectiveness of a self-management support program for Thais with type 2 diabetes: Evaluation according to the RE-AIM framework. Nursing & Health Sciences. 2015;17(3):362-9.
43. Kennedy A, Bower P, Reeves D, Blakeman T, Bowen R, Chew-Graham C, et al. Implementation of self management support for long term conditions in routine primary care settings: cluster randomised controlled trial. BMJ : British Medical Journal. 2013;346:f2882.
44. Kennedy A, Chew-Graham C, Blakeman T, Bowen A, Gardner C, Protheroe J, et al. Delivering the WISE (Whole Systems Informing Self-Management Engagement) training package in primary care: learning from formative evaluation. Implement Sci. 2010;5:7-.
45. McGeoch G, Willsman K, Dowson C, Town G, Frampton C, F. M, et al. Self-management plans in the primary care of patients with chronic obstructive pulmonary disease. Respirology. 2006;11(5):611-8.
46. McLean W, Gillis J, Waller R. The BC Community Pharmacy Asthma Study: A study of clinical, economic and holistic outcomes influenced by an asthma care protocol provided by specially trained community pharmacists in British Columbia. Canadian respiratory journal. 2003;10(4):195-202.
47. Mehuys E, Van Bortel L, De Bolle L, Van Tongelen I, Annemans L, Remon J, et al. Effectiveness of pharmacist intervention for asthma control improvement. The European respiratory journal. 2008;31(4):790-9.
48. Mehuys E, Van Bortel L, De Bolle L, Van Tongelen I, Annemans L, Remon J, et al. Effectiveness of a community pharmacist intervention in diabetes care: a randomized controlled trial. Journal of clinical pharmacy and therapeutics. 2011;36(5):602-13.
49. Meland E, Laerum E, Ulvik R. Effectiveness of two preventive interventions for coronary heart disease in primary care. Scandinavian journal of primary health care. 1997;15(1):57-64.
50. Meland E, Maeland J, Laerum E. The importance of self-efficacy in cardiovascular risk factor change. Scandinavian journal of public health. 1999;27(1):11-7.
51. Mitchell K, Johnson-Warrington V, Apps L, Bankart J, Sewell L, Williams J, et al. A self-management programme for COPD: a randomised controlled trial. The European respiratory journal. 2014;44(6):1538-47.
52. Dritsaki M, Johnson-Warrington V, Mitchell K, Singh S, Rees K. An economic evaluation of a self-management programme of activity, coping and education for patients with chronic obstructive pulmonary disease. Chron Respir Dis. 2016;13(1):48-56.
53. Morgan M, Dunbar J, Reddy P, Coates M, Leahy R. The TrueBlue study: is practice nurse-led collaborative care effective in the management of depression for patients with heart disease or diabetes? BMC family practice. 2009;10:46.
54. Morgan M, Coates M, Dunbar J, Reddy P, Schlicht K, Fuller J. The TrueBlue model of collaborative care using practice nurses as case managers for depression alongside diabetes or heart disease: a randomised trial. BMJ open. 2013;3(1).
55. Moss-Morris R, McAlpine L, Didsbury L, Spence M. A randomized controlled trial of a cognitive behavioural therapy-based self-management intervention for irritable bowel syndrome in primary care. Psychological medicine. 2010;40(1):85-94.

56. Chilcot J, Moss-Morris R. Changes in illness-related cognitions rather than distress mediate improvements in irritable bowel syndrome (IBS) symptoms and disability following a brief cognitive behavioural therapy intervention. Behaviour research and therapy. 2013;51(10):690-5.
57. Murphy A, Cupples M, Smith S, Byrne M, Byrne M, Newell J. Effect of tailored practice and patient care plans on secondary prevention of heart disease in general practice: cluster randomised controlled trial. BMJ (Clinical research ed). 2009;339:b4220.
58. Murphy A, Cupples M, Smith S, Byrne M, Leathem C, Byrne M. The SPHERE Study. Secondary prevention of heart disease in general practice: protocol of a randomised controlled trial of tailored practice and patient care plans with parallel qualitative, economic and policy analyses. [ISRCTN24081411]. Current controlled trials in cardiovascular medicine. 2005;6:11.
59. Gillespie P, O'Shea E, Smith S, Cupples M, Murphy A. A comparison of medical records and patient questionnaires as sources for the estimation of costs within research studies and the implications for economic evaluation. Family practice. 2016.
60. Olry de Labry Lima A, Bermudez Tamayo C, Pastor Moreno G, Bolivar Munoz J, Ruiz Perez I, Johri M, et al. Effectiveness of an intervention to improve diabetes self-management on clinical outcomes in patients with low educational level. Gaceta sanitaria / SESPAS. 2016.
61. Partapsingh V, Maharaj R, Rawlins J. Applying the Stages of Change model to Type 2 diabetes care in Trinidad: A randomised trial. Journal of Negative Results in BioMedicine. 2011;10(1).
62. Richards A, Barkham M, Cahill J, Richards D, Williams C, Heywood P. PHASE: a randomised, controlled trial of supervised self-help cognitive behavioural therapy in primary care. The British journal of general practice : the journal of the Royal College of General Practitioners. 2003;53(495):764-70.
63. Rosemann T, Joos S, Laux G, Gensichen J, Szecsenyi J. Case management of arthritis patients in primary care: a cluster-randomized controlled trial. Arthritis and rheumatism. 2007;57(8):1390-7.
64. Smit A, Tiemens B, Ormel J, Kluiter H, Jenner J, van de Meer K, et al. Enhanced treatment for depression in primary care: first year results on compliance, self-efficacy, the use of antidepressants and contacts with the primary care physician. Primary Care & Community Psychiatry. 2005;10(2):39-49.
65. Stant A, TenVergert E, Kluiter H, Conradi H, Smit A, Ormel J. Cost-effectiveness of a psychoeducational relapse prevention program for depression in primary care. The journal of mental health policy and economics. 2009;12(4):195-204.
66. Striegel-Moore R, Wilson G, DeBar L, Perrin N, Lynch F, Rosselli F, et al. Cognitive Behavioral Guided Self-Help for the Treatment of Recurrent Binge Eating. Journal of consulting and clinical psychology. 2010;78(3):312-21.
67. DeBar L, Striegel-Moore R, Wilson G, Perrin N, Yarborough B, Dickerson J, et al. Guided Self-Help Treatment for Recurrent Binge Eating: Replication and Extension. Psychiatric Services. 2011;62(4):367-73.
68. Sturt J, Whitlock S, Fox C, Hearnshaw H, Farmer A, Wakelin M, et al. Effects of the Diabetes Manual 1:1 structured education in primary care. Diabetic medicine : a journal of the British Diabetic Association. 2008;25(6):722-31.
69. Tiessen A, Smit A, Broer J, Groenier K, van der Meer K. Randomized controlled trial on cardiovascular risk management by practice nurses supported by self-monitoring in primary care. BMC family practice. 2012;13:90.
70. Tiessen A, Vermeulen K, Broer J, Smit A, van der Meer K. Cost-effectiveness of cardiovascular risk management by practice nurses in primary care. BMC public health. 2013;13:148.
71. van Dijk-de Vries A, van Bokhoven M, Winkens B, Terluin B, Knottnerus J, van der Weijden T, et al. Lessons learnt from a cluster-randomised trial evaluating the effectiveness of Self-Management Support (SMS) delivered by practice nurses in routine diabetes care. BMJ open. 2015;5(6):e007014.
72. Von Korff M, Balderson B, Saunders K, Miglioretti D, Lin E, Berry S, et al. A trial of an activating intervention for chronic back pain in primary care and physical therapy settings. Pain. 2005;113(3):323-30.

73. Waite P, McManus F, Shafran R. Cognitive behaviour therapy for low self-esteem: a preliminary randomized controlled trial in a primary care setting. Journal of behavior therapy and experimental psychiatry. 2012;43(4):1049-57.
74. Watkins E, Taylor R, Byng R, Baeyens C, Read R, Pearson K, et al. Guided self-help concreteness training as an intervention for major depression in primary care: a Phase II randomized controlled trial. Psychological medicine. 2012;42(7):1359-71.
75. Watson PB, Town GI, Holbrook N, Dwan C, Toop LJ, Drennan CJ. Evaluation of a self-management plan for chronic obstructive pulmonary disease. The European respiratory journal. 1997;10(6):1267-71.
76. Williams C, Wilson P, Morrison J, McMahon A, Walker A, Allan L, et al. Guided self-help cognitive behavioural therapy for depression in primary care: a randomised controlled trial. PloS one. 2013;8(1):e52735.
77. Wood-Baker R, McGlone S, Venn A, Walters E. Written action plans in chronic obstructive pulmonary disease increase appropriate treatment for acute exacerbations. Respirology. 2006;11(5):619-26.
78. Yardley L, Donovan-Hall M, Smith H, Walsh B, Mullee M, Bronstein A. Effectiveness of primary care-based vestibular rehabilitation for chronic dizziness. Annals of internal medicine. 2004;141(8):598-605.
79. Zimmermann T, Puschmann E, van den Bussche H, Wiese B, Ernst A, Porzelt S, et al. Collaborative nurse-led self-management support for primary care patients with anxiety, depressive or somatic symptoms: Cluster-randomised controlled trial (findings of the SMADS study). International journal of nursing studies. 2016;63:101-11.
